# Separation-related rapid nuclear transport of DNA/RNA heteroduplex oligonucleotide: unveiling distinctive intracellular trafficking

**DOI:** 10.1016/j.omtn.2020.11.022

**Published:** 2020-12-03

**Authors:** Daisuke Ono, Ken Asada, Daishi Yui, Fumika Sakaue, Kotaro Yoshioka, Tetsuya Nagata, Takanori Yokota

**Affiliations:** 1 Department of Neurology and Neurological Science, Graduate School of Medical and Dental Sciences and Center for Brain Integration Research, Tokyo Medical and Dental University, 1-5-45 Yushima, Bunkyo-Ku, Tokyo 113-8519, Japan

**Keywords:** DNA:RNA hybrids, endosomal escape, cytosolic release

## Abstract

DNA/RNA heteroduplex oligonucleotide (HDO), composed of DNA/locked nucleic acid (LNA) antisense oligonucleotide (ASO) and complementary RNA, is a next-generation antisense therapeutic agent. HDO is superior to the parental ASO in delivering to target tissues, and it exerts a more potent gene-silencing effect. In this study, we aimed to elucidate the intracellular trafficking mechanism of HDO-dependent gene silencing. HDO was more preferably transferred to the nucleus after transfection compared to the parental ASO. To determine when and where HDO is separated into the antisense strand (AS) and complementary strand (CS), we performed live-cell time-lapse imaging and fluorescence resonance energy transfer (FRET) assays. These assays demonstrated that HDO had a different intracellular trafficking mechanism than ASO. After endocytosis, HDO was separated in the early endosomes, and both AS and CS were released into the cytosol. AS was more efficiently transported to the nucleus than CS. Separation, endosomal release, and initiation of nuclear transport were a series of time-locked events occurring at a median of 30 s. CS cleavage was associated with efficient nuclear distribution and gene silencing in the nucleus. Understanding the unique intracellular silencing mechanisms of HDO will help us design more efficient drugs and might also provide insight into innate DNA/RNA cellular biology.

## Graphical Abstract

**Figure:**
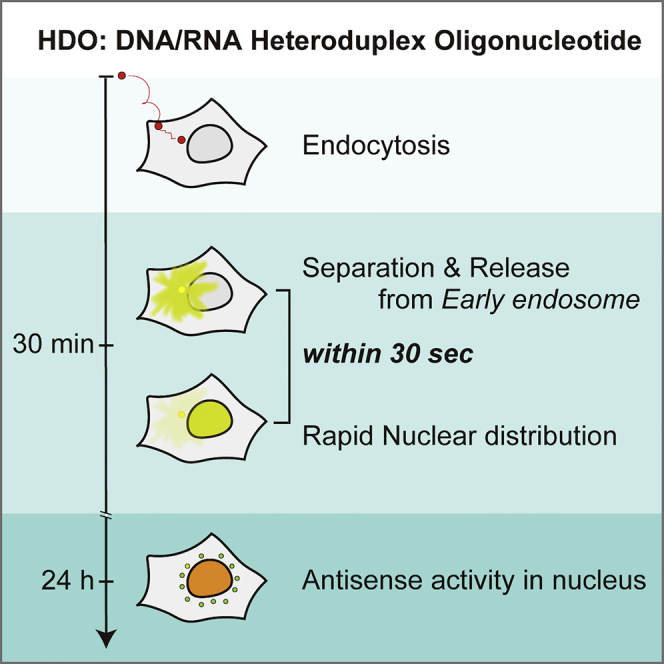

## Teaser

DNA/RNA heteroduplex oligonucleotide (HDO) is a newly developed antisense therapy. Here, Ono et al. demonstrated HDO was separated in and released from early endosomes and was efficiently transported into the nucleus. The double-stranded structure of HDO has dramatically changed the intracellular mechanism compared with its parent antisense oligonucleotide.

## Introduction

Antisense oligonucleotide (ASO) is a single-stranded therapeutic oligonucleotide that modulates RNA functions by binding to the targeted RNA through Watson-Crick base pairing.^1^^,^^2^ ASO is typically designed as a “gapmer” structure, where wings of chemically modified nucleotides flank both sides of a central portion of DNA. ASO gapmer with phosphorothioate (PS) backbone is highly potent and has recently been applied to clinical settings.^3^^,^^4^

We recently developed a novel highly efficient oligonucleotide, DNA/RNA heteroduplex oligonucleotide (HDO), which is composed of an antisense gapmer (DNA nucleotides flanked by LNAs) and complementary RNA (cRNA).^5^, ^6^, ^7^ HDO showed improved delivery to the target tissue by conjugating tocopherol to the cRNA strand. Additionally, Toc-HDO presented 4.8 times higher gene silencing effect than parental ASO, revealed after measuring the delivered oligonucleotide content.^5^ MicroRNA-targeting HDO (HDO-antimiR) displayed high potency in cleaving the target microRNA, whereas the parental ASO exerted its potency via a steric blocking (not cleaving) mechanism.^6^ Additionally, HDO-antimiR conjugated with GalNAc was more potent in the liver than the parent ASO conjugated with GalNAc, where delivery efficiency of HDO was comparable to that of ASO.^6^ These findings suggested that HDO may have a distinct intracellular trafficking pathway and processing machinery different from the single-stranded ASO.

Intracellular trafficking pathways of oligonucleotides are diverse and depend on their structures, such as chemical modifications, ligand conjugations, and association with nanocarriers.^8^, ^9^, ^10^, ^11^ For therapeutic applications, intracellular trafficking of ASO gapmers with PS backbones (referred to as PS-ASOs) has been intensely investigated.^8^^,^^11^ Cells internalize PS-ASOs by endocytosis; they are trafficked from early endosomes to late endosomes, and finally to lysosomes.^12^^,^^13^ PS-ASOs have to be released from those endocytic vesicles into the cytosol and nucleus, where they bind to the target mRNA and cleave it by RNase H1.^8^^,^^11^^,^^14^^,^^15^

In our previous study, we investigated the intracellular mechanism of HDO.^5^ From the imaging studies *in vivo*, we suspected that the antisense strand (AS) of HDO was more robustly distributed into the nucleus compared with the parental ASO. Therefore, we hypothesized that an effective gene silencing by HDO could be attributed not only to the efficiency of cellular uptake but also to the distinctive intracellular localization patterns. Unlike single-stranded ASO, double-stranded HDO has to be separated into AS and complementary strand (CS) in the cell in order for AS to bind to the target RNAs and exert antisense activity. For the elucidation of these intracellular trafficking mechanisms of HDO, there are technical limitations—a conventional snapshot imaging cannot detect the time and site of HDO separation. To tackle this problem, we performed live-cell time-lapse imaging utilizing fluorescence resonance energy transfer (FRET) assay and demonstrated a unique intracellular silencing mechanism by HDO.

## Results

### Efficient gene silencing of HDO in the nucleus after cytosolic delivery

**Figure 1:**
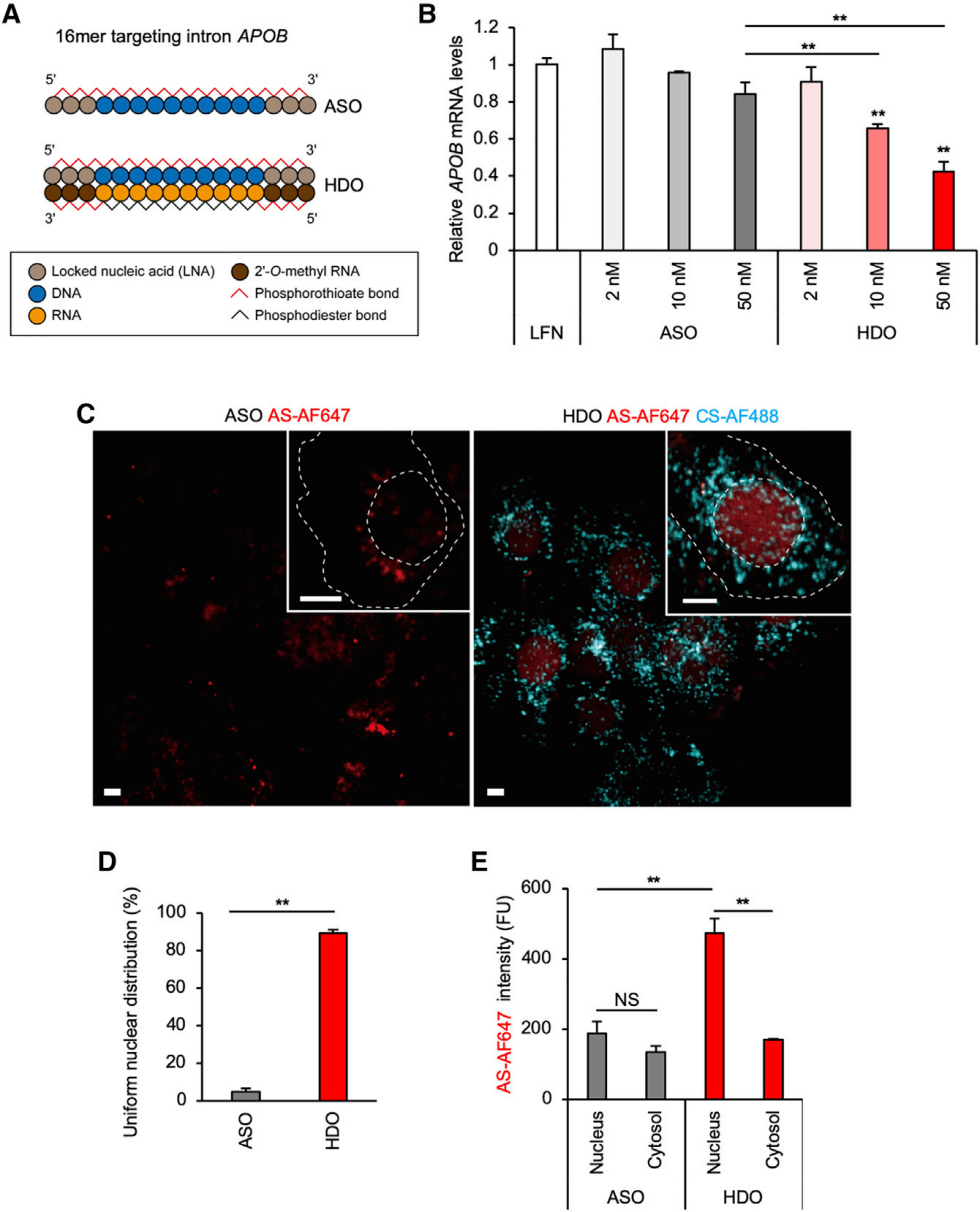
Efficient gene silencing of HDO in the nucleus after cytosolic delivery (A) Design of ASO and HDO, targeting intron region of *APOB* pre-mRNA. The ASO is the 16-mer gapmer in which 10 DNA oligonucleotides are flanked by 3 LNA oligonucleotides, and all internucleotide linkages were modified by phosphorothioate (PS) substitution. In HDO, the complementary RNA strand is flanked by PS-modified 2′-*O*-methyl RNAs, which are complementary to LNA.^5^ (B) Quantitative real-time PCR analysis of relative *APOB* mRNA levels was normalized to those of *GAPDH* mRNA 24 h after transfection of intron-targeting HDO or ASO (∗∗p < 0.01 versus Lipofectamine [LFN] control; n = 3; mean ± SEM). (C) Representative images 24 h after transfection of the cells with 50 nM ASO or HDO. AF647 labels the antisense strand (AS) of HDO, and AF488 labels the complementary strand (CS), where FRET does not occur. AF647 signals (red) were excited by a 646 nm laser and detected through a 700 (663–738) nm filter. AF488 signals (cyan) were excited by a 488 nm laser and detected through a 525 (500–550) nm filter. Dotted lines represent nuclear or plasma membrane outlined by differential interference contrast (DIC) images. Bar, 10 μm. (D) Percentage of cells with uniform nuclear distribution 24 h after transfection with 50 nM ASO or HDO. (E) Mean intensities of AS-AF647 in the nucleus or cytosol 24 h after transfection with 50 nM ASO or HDO, presented as absolute values normalized to background levels. (∗∗p < 0.01; NS, not significant; n = 3 images for each 50 cells).

To address our hypothesis that HDO is more efficiently transferred into the nucleus, we designed ASO and HDO molecules that targeted the intron region of *APOB* pre-mRNA (Figure 1A), which is localized in the nucleus.^15^ We evaluated how a spatially specifically introduced HDO or ASO can regulate nuclear expressing target pre-mRNA in human hepatocellular carcinoma Huh-7 cells transfected using Lipofectamine RNAiMAX, which effectively delivers small interfering RNAs (siRNAs) or single-stranded nucleotides into the cytosol.^16^, ^17^, ^18^ *APOB* expression was measured 24 h after transfection, using quantitative real-time PCR. Our results showed a dose-dependent nuclear silencing effect of HDO, whereas no significant effect was observed with the parent ASO (Figure 1B; Figure S1).

Next, to visualize the nuclear distribution of HDO, we labeled AS with the fluorophore AF647 (excitation/emission: 650/665 nm), and CS with AF488 (490/525 nm), where excitation wavelengths are so far that the FRET phenomenon seldom occurs. Imaging results 24 h after HDO transfection showed a completely different distribution pattern compared to ASO (Figure 1C). An intense and well-demarcated nuclear signal of AS was observed in almost all HDO-transfected cells. Additionally, a dotted signal of CS, accumulated dominantly in the cytosol, was detected. Meanwhile, the same dose of ASO presented just a diffused or partly dotted weak distribution throughout the cells.

**Figure 2:**
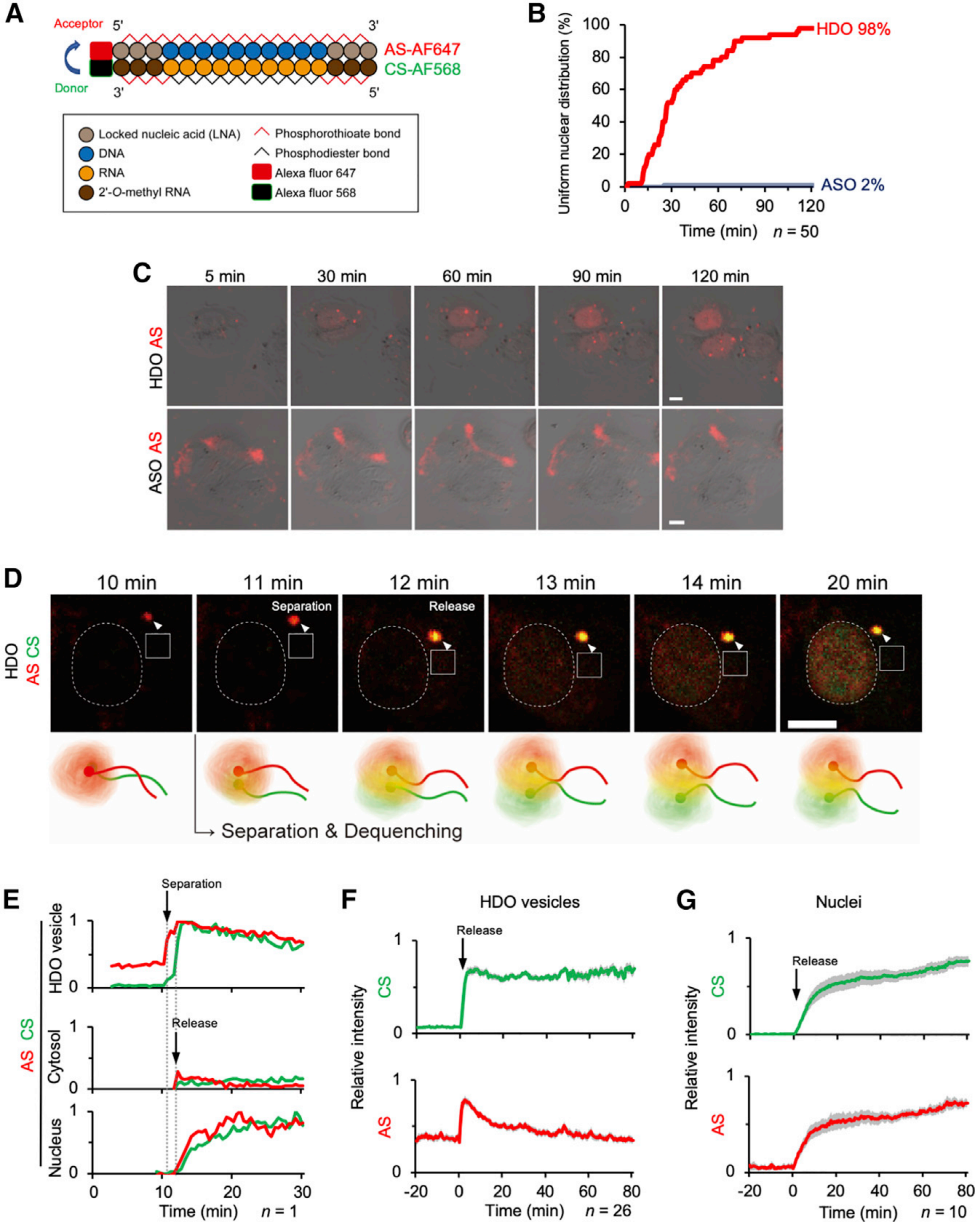
Separation and rapid transport of HDO into the nucleus (A) Design of dye-conjugated HDO targeting intron *APOB* with FRET system. AF647 labels the AS of HDO, and AF568 labels the CS, where CS-AF568 signals become quenched. (B) Sequential changes in the percentage of cells with uniform nuclear distribution after 50 nM transfection of HDO or ASO. (n = 50). (C) Representative live-cell images within 2 h after transfection with 50 nM HDO or ASO, presented as merged images of DIC and AS-AF647 (red). AF647 signals were excited by a 646 nm laser and detected through a 700 (663–738) nm filter. Bar, 10 μm. (D) Live-cell time-lapse images of the HDO-releasing vesicle (arrowhead). As shown in the bottom scheme, AF568 signal (green) represents separated CS, and AF647 signal (red) corresponds to the AS signal from the wound or isolated form. Therefore, newly observed yellow signal (colocalization of red and green) means separation of HDO. Dotted circles and squares represent nuclear and cytosolic areas, respectively, which are shown in (E). Images were acquired just after transfection with 50 nM HDO, every 30 s. AF647 signals were excited by a 646 nm laser and detected through a 700 (663–738) nm filter. AF568 signals were excited by a 560 nm laser and detected through a 595 (570–620) nm filter. Bar, 10 μm. (E) Sequential changes of AS-AF647 (red) and CS-AF568 (green) signals in the nucleus, cytosol, and the HDO-releasing vesicle of the cell shown in (D). Mean intensities of each region are presented as relative values (0–1), with 0 being the background intensity and 1 being the highest intensity value for each object (except for the cytosol using the highest intensity of the nucleus). (F and G) Mean relative intensity of AS-AF647 and CS-AF568 in HDO-releasing vesicles (F) and nuclei (G). *t* = 0 is set just before the releases started. (F, n = 26; G, n = 10 G; ± SEM, shaded areas). Results were pooled from three experiments per condition in (F).

Before the time-lapse imaging we will show in Figure 2, we defined the uniform and well-demarcated nuclear AS signals as “nuclear transport” and counted them (Figure 1D). HDO showed a high uniform nuclear distribution rate, which was concordant with its potency (Figure 1B). We then quantified the mean intensity in both the nucleus and cytosol (Figure 1E). HDO presented strong preference for the nucleus. On the other hand, ASO was distributed to the nucleus and cytosol without significant preference. Thus, we supposed that the uniform and well-demarcated nuclear localization was a distinctive character of HDO, associated with nuclear potency.

Additionally, in co-transfection experiments with HDO and ASO, we confirmed that HDO was also superiorly distributed compared to ASO (Figure S2). Fluorescent dyes transfected without the oligonucleotide conjugation were not detectable in the cells (Figure S3). To evaluate delivery methods other than lipid transfection, we performed gymnotic (free uptake) delivery of ASO or HDO, but it presented no significant nuclear distribution (Figure S4).

### Separation and rapid transport of HDO into the nucleus

To observe the separation of HDO, we designed an HDO composed of AF647-labeled AS and AF568-labeled CS and applied the FRET mechanism^19^, ^20^, ^21^, ^22^, ^23^ (Figure 2A). Excitation/emission wavelengths of these dyes are 650/665 and 578/603 nm, respectively, which are close enough for FRET to occur. In the double-stranded wound form, the energy absorbed by CS-AF568 after excitation with a 560 nm laser was transferred to AS-AF647. In other words, CS-AF568 signal became quenched, and AS-AF647 produced a signal through FRET. If HDO was separated, the isolated CS-AF568 could not transfer the energy to AS-AF647, and thus the CS-AF568 signal would increase and become dequenched. We confirmed the FRET and dequenching signals in our experimental system by measuring the fluorescence intensity of dye-conjugated HDO in solution without cell or transfection reagent (Figure S5).

Live-cell time-lapse imaging was performed every 30 s for 120 min (Figure 2). Uniform nuclear distribution of AS was observed in 98% of HDO- and only 2% of ASO-transfected cells (Figure 2B). We repeated the experiments and deduced the following reproducible results. Immediately after the transfection, ASO-containing liposomes were visualized in the background, and a few of them were endocytosed. Cloud-like weak ASO signals were observed without preference to the nucleus or cytosol, which presented no remarkable change within 2 h (Figure 2C).

HDO-containing liposomes were also visualized in the background, where the majority of CS-AF568 signal was quenched by FRET. A few minutes after the transfection, several liposomes were endocytosed into the cell and transported from the periphery toward the perinuclear region of the cytosol. Then, in one of the endocytosed HDO vesicles, a sudden appearance of the CS-AF568 signal was detected with a transient increase of the AS-AF647 signal. Almost simultaneously, both AS and CS were released into the cytosol and subsequent nuclear transport started (Figures 2D and 2E; Video S1).

Video S1. Separation, cytosolic release, and rapid nuclear transport of HDO Live cell time-lapse images of the HDO-releasing vesicle presented in Figure 2D, E. Images were taken, every 30 sec, just after transfection with 50 nM HDO targeting intron *APOB*, composed of antisense strand-AF647 (red) and complementary strand-AF568 (green). AF647 signals were excited by a 646 nm laser and detected through a 700 (663-738) nm filter. AF568 signals were excited by a 560 nm laser and detected through a 595 (570-620) nm filter. Bar = 10 μm.

We considered the transient increase of the AS-AF647 signal in the vesicle at the moment of the release as cancellation of self-quenching. Densely packed fluorophores, such as in liposomes, quench themselves, and this self-quenching is cancelled by the release from the vesicles.^24^^,^^25^ We subsequently defined the HDO release as a sudden increase of the AS signal in the cytosol and identified an HDO-releasing vesicle by the transient increase of the AF647 signal. We traced and measured subsequent changes of signal intensities in the HDO-releasing vesicles and nuclei, where *t* = 0 was reset just before the release, and the mean relative intensity was calculated with reference to the previous study (Figures 2F and 2G).^24^ The increase of the AS-AF647 signal was transient and corresponded to the cancellation of the self-quenching. The sudden and sharp increase of CS-AF568 signal just after the HDO release was reproducibly observed. Then, we confirmed that this newly generated CS-AF568 signal meant dequenching of the FRET signal, as a result of the HDO separation (Figure S6).

Cytosolic signal from the AS was weak and transient, which was swallowed up into the nucleus soon after and depleted within a few minutes (Figures 2D and 2E; Video S1). The nuclear distribution of both strands was almost homogeneous, which corresponded to the uniform nuclear distribution observed 24 h after the transfection, as mentioned above (Figures 1C and 1D). Further release from other vesicles followed, whereas the second release from the same vesicle was seldom observed. If we increased the sensitivity of fluorescence detection settings, the inflow signal from the releasing vesicles was observed (Videos S2 and S3; Figure S7). These sequential events—the separation, release, and nuclear transport of both strands—always occurred in that order. The separation occurred at a median of 27 min, and the median interval of separation to initiation of nuclear distribution was as short as 30 s (n = 50). The same phenomena were observed with 10 nM HDO, which was a comparable dose to 50 nM ASO (Figure S8). We also obtained similar results with other sequences of HDO, targeting the intron *SNCA* mRNA (Figures S9 and S10A–S10G) and a sequence without any target gene (Figures S11A–S11G).

Video S2. Inflow signals from HDO-releasing vesicles (A, B) Live cell time-lapse images of cells which showed inflow signals from HDO-releasing vesicles, Images were taken in a high sensitivity setting compared to experiments in Figure 2, every 30 sec, just after transfection with 50 nM HDO targeting intron *APOB*, composed of antisense strand-AF647 (red) and complementary strand-AF568 (green). AF647 signals were excited by a 646 nm laser and detected through a 700 (663-738) nm filter. AF568 signals were excited by a 560 nm laser and detected through a 595 (570-620) nm filter. Bar = 10 μm.

Video S3. Inflow signals from HDO-releasing vesicles (A, B) Live cell time-lapse images of cells which showed inflow signals from HDO-releasing vesicles, Images were taken in a high sensitivity setting compared to experiments in Figure 2, every 30 sec, just after transfection with 50 nM HDO targeting intron *APOB*, composed of antisense strand-AF647 (red) and complementary strand-AF568 (green). AF647 signals were excited by a 646 nm laser and detected through a 700 (663-738) nm filter. AF568 signals were excited by a 560 nm laser and detected through a 595 (570-620) nm filter. Bar = 10 μm.

### Release of HDO from early endosomes

**Figure 3:**
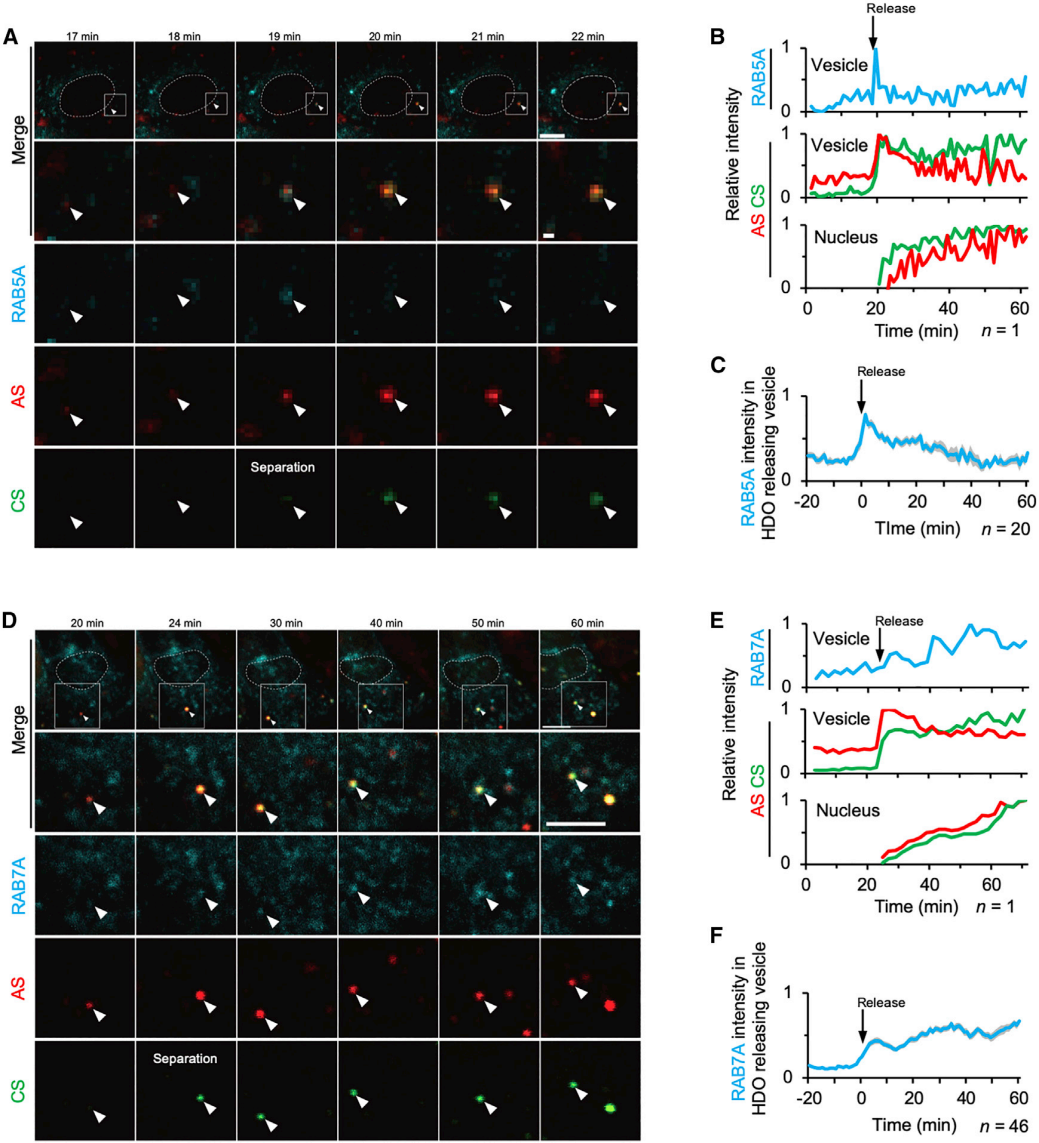
Release of HDO from early endosomes (A and D) Live-cell time-lapse images of the HDO-releasing vesicle (arrowheads). Cells expressing GFP-labeled RAB5A (A–C) or RAB7A (D–F) (cyan) were transfected with 50 nM HDO targeting intron *APOB* (AS-AF647, CS-AF568). Dotted circles represent nuclei outlined by DIC images. Images were taken just after transfection, every 1 min (A–C and F) or 2 min (D and E). GFP signals were excited by a 488 nm laser and detected through a 525 (500–550) nm filter. AF647 signals (red) were excited by a 646 nm laser and detected through a 700 (663–738) nm filter. AF568 signals (green) were excited by a 560 nm laser and detected through a 595 (570–620) nm filter. Bar, 10 μm, except the second row of (A), which is 1 μm. (B and E) Sequential changes of AF647 (red), AF568 (green), and RAB5A (B) or RAB7A (E) (cyan) signals in the HDO-releasing vesicles and the nucleus of the cell shown in (A) or (D), respectively. Mean intensities of each region were presented as relative values. (C and F) Mean relative intensity of RAB5A (C) or RAB7A (F) in HDO-releasing vesicles. *t* = 0 is set just before the release started. (C, n = 20; F, n = 46; ± SEM shown as shaded areas). Results were pooled from three experiments per condition.

Cells expressing GFP-labeled endosome markers were transfected with HDO, and subsequent live-cell imaging was performed to identify the nature of the HDO-releasing vesicle. At the time of the separation, strong but transient co-localization of the releasing vesicle with RAB5A, the early endosome marker, was observed (Figures 3A–3C; Video S4). In contrast, RAB7A, a late endosome marker, was gradually co-localized with the releasing vesicle several minutes after the separation (Figures 3D–3F; Video S5). These observations were reproducibly confirmed by two additional sequences and the other cell line (Figures S10H, S10I, S11H, and S11I). Therefore, we considered that HDO was separated in and released from early endosomes.

Video S4. Co-localization of HDO vesicle foci with early endosomes Live cell time-lapse images of the HDO releasing vesicle presented in Figures 3A and 3B. Cells expressing GFP-labeled *RAB5A* (cyan) were transfected with 50 nM HDO targeting intron *APOB* (antisense strand-AF647 (red), complementary strand-AF568 (green)). GFP signals were excited by a 488 nm laser and detected through a 525 (500-550) nm filter. AF647 signals were excited by a 646 nm laser and detected through a 700 (663-738) nm filter. AF568 signals were excited by a 560 nm laser and detected through a 595 (570-620) nm filter. Images were taken just after transfection, every 1 min. Bar = 10 μm.

Video S5. Co-localization of HDO vesicle foci with late endosomes Live cell time-lapse images of the HDO releasing vesicle presented in Figure 3D and 3E. Cells expressing GFP-labeled *RAB7A* (cyan) were transfected with 50 nM HDO targeting intron *APOB* (antisense strand-AF647 (red), complementary strand-AF568 (green)). GFP signals were excited by a 488 nm laser and detected through a 525 (500-550) nm filter. AF647 signals were excited by a 646 nm laser and detected through a 700 (663-738) nm filter. AF568 signals were excited by a 560 nm laser and detected through a 595 (570-620) nm filter. Images were taken just after transfection, every 2 min. Bar = 10 μm.

### Accumulation of AS in the nucleus and CS in lysosomes

**Figure 4:**
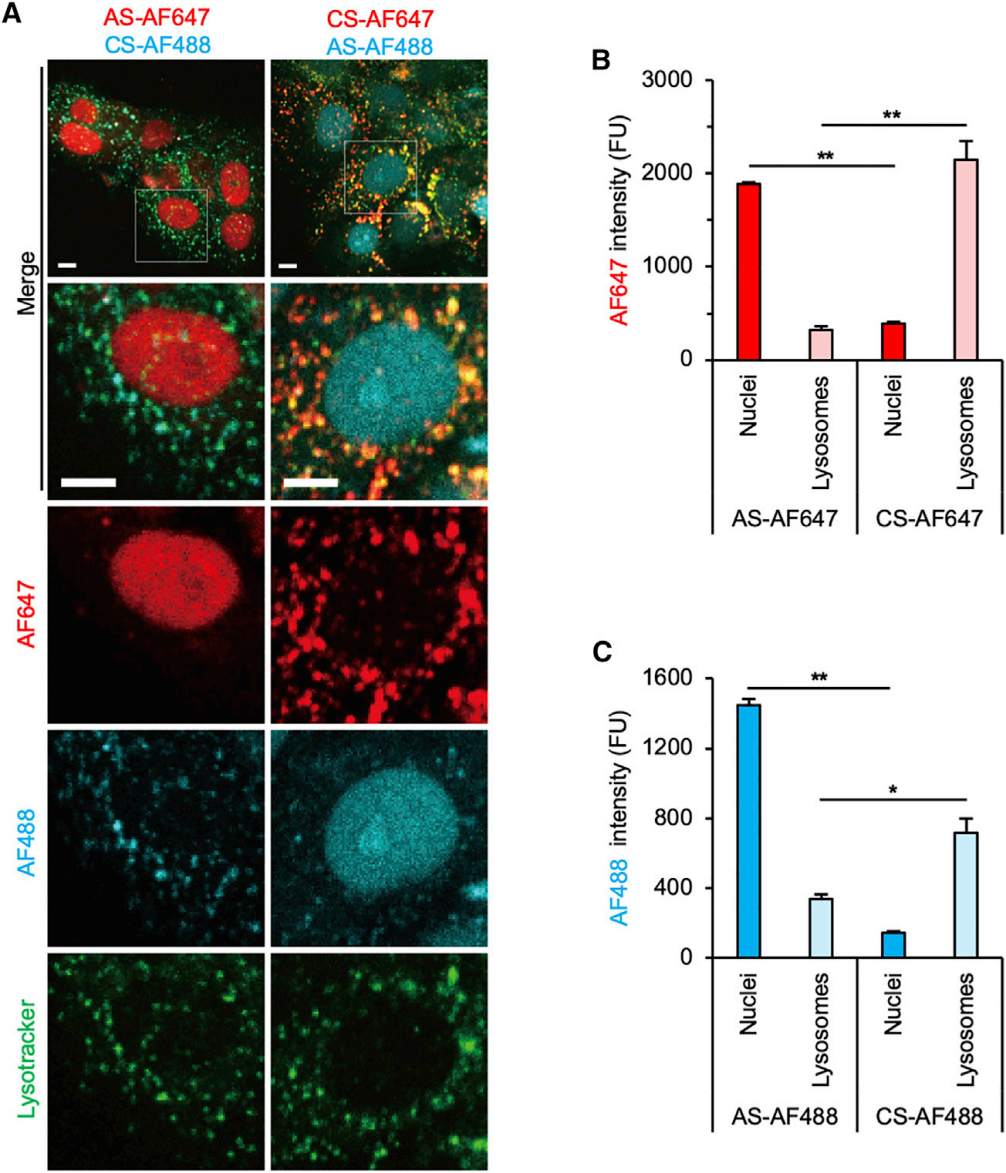
Accumulation of AS in the nucleus and CS in lysosomes (A) Representative images 24 h after transfection with 50 nM HDO targeting intron *APOB*, composed of AS-AF647 and CS-AF488 (left), or CS-AF647 and AS-AF488 (right). Lysosomes were labeled with lysotracker-RFP (green), which were excited by a 560 nm laser and detected through a 595 (570–620) nm filter. AF647 signals (red) were excited by a 646 nm laser and detected through a 700 (663–738) nm filter. AF488 signals (cyan) were excited by a 488 nm laser and detected through a 525 (500–550) nm filter. Bar, 10 μm. (B and C) Mean signal intensities of AF647 (B) or AF488 (C) in nuclei and lysosomes. Measurements from the experiment (A) presented as absolute values normalized to background levels. (∗p < 0.05, ∗∗p < 0.01; n = 3 images for each 50 cells, or 150 lysosomes; mean ± SEM).

To evaluate HDO trafficking after the endosomal release, we labeled lysosomes with RFP-lysotracker. HDO composed of AS-AF647/CS-AF488 or reverse pair AS-AF488/CS-AF647 was imaged at 24 h (Figure 4A). Signal intensities in nuclei or lysosomes were measured and presented as the mean of absolute values so that we could compare AS and CS by the same fluorescent dye (Figures 4B and 4C). Both dyes presented the same tendency that more AS accumulated in nuclei than in lysosomes and more CS accumulated in lysosomes than in nuclei.

### CS cleavage and efficient gene silencing in the nucleus

**Figure 5:**
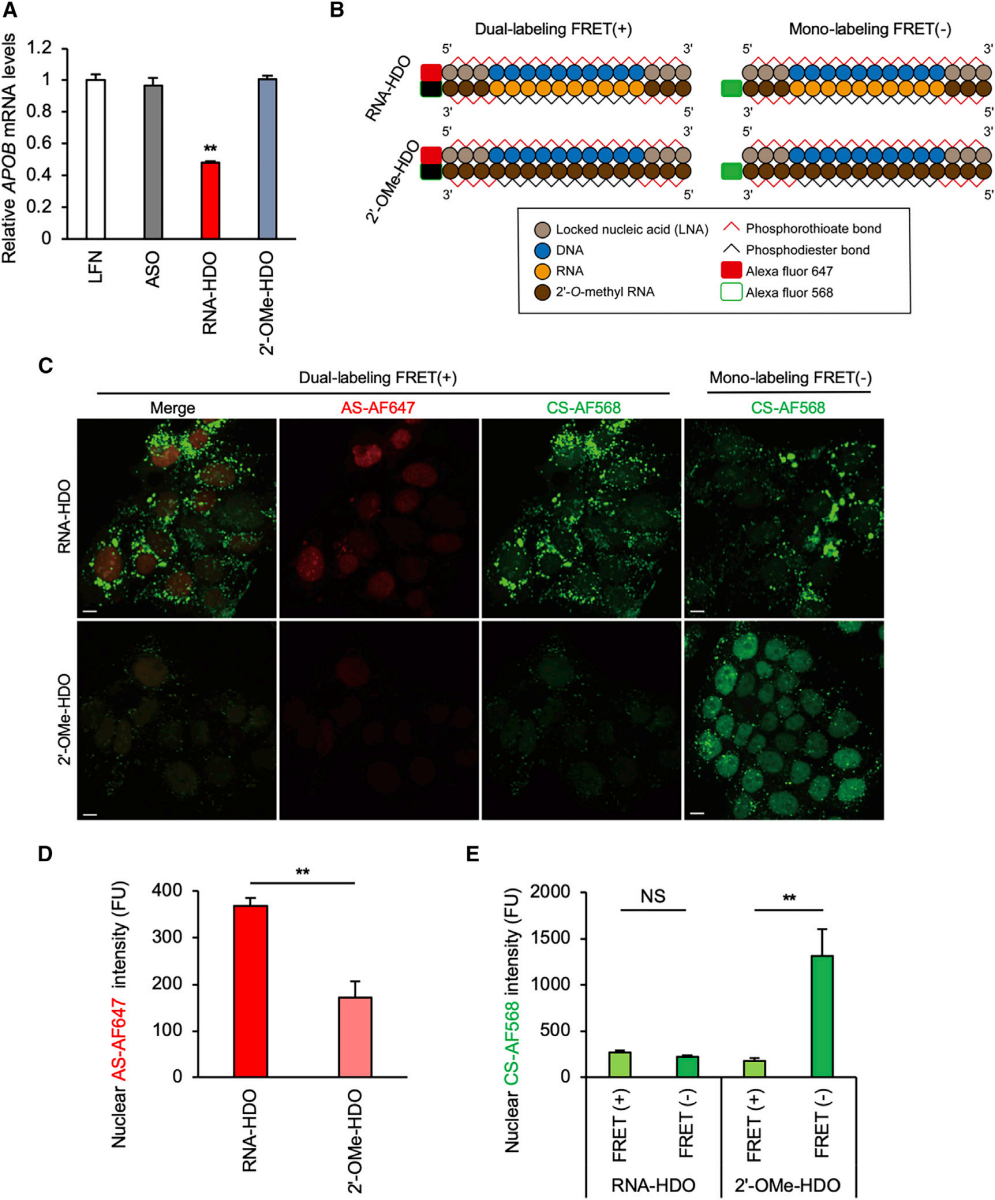
Cleavage of CS and efficient gene silencing in the nucleus To evaluate the effect of CS degradation, we used RNase-resistant full 2′-OMe-strand as CS. (A) Quantitative real-time PCR analysis of relative *APOB* mRNA levels normalized to *GAPDH* mRNA 24 h after transfection with 50 nM ASO, RNA-HDO, or 2′-OMe-HDO targeting intron *APOB* (∗∗p < 0.01 versus LFN control; n = 4; mean ± SEM). (B) Designs of dye-conjugated HDOs, where RNA or full 2′-OMe was labeled with AF568, and AS was labeled with AF647 (dual-labeling with FRET) or not labeled (mono-labeling without FRET). (C) Images 24 h after transfection with 50 nM HDOs. AF647 signals (red) were excited by a 646 nm laser and detected through a 700 (663–738) nm filter. AF568 signals (green) were excited by a 560 nm laser and detected through a 595 (570–620) nm filter. Images were obtained by weaker sensitivity settings than those of Figure 2 to maximize a slight difference of nuclear signals between RNA-HDO and 2′-OMe-HDO. Bar, 10 μm. (D and E) Mean nuclear intensities of AS-AF647 (D) or CS-AF568 (E) presented as absolute values normalized to background levels (∗∗p < 0.01; NS, not significant; n = 3 images for each 50 cells; mean ± SEM).

To evaluate the significance of CS cleavage for gene silencing in the nucleus, we applied the RNase-resistant 2′-*O*-methyl sugar modification (2′-OMe). We confirmed that CS composed of full 2′-OMe was not cleaved *in vitro* (Figure S12A), which is in accordance with a previous *in vivo* study.^5^ In the present study, HDO with a modified full 2′-OMe CS (2′-OMe-HDO) showed a much lower gene silencing effect in the nucleus than the default HDO (RNA-HDO) (Figure 5A; Figures S12B and S13). To discriminate whether HDO was wound or separated, we generated mono-labeled-HDO (CS-AF568) without FRET, as well as dual-labeled HDO (AS-AF647/CS-AF568) with FRET (Figure 5B). Mono-labeled AF568 signals reflected the amount of CS in both wound and separated forms, whereas in dual-labeled HDO, AF568 signal was quenched in the wound form but observed in the separated form.

Imaging and subsequent measurements of nuclear intensity were performed (Figures 5C–5E). 2′-OMe-HDO presented a less intense distribution of AS into the nucleus 24 h after transfection (Figure 5D) than RNA-HDO. Nuclear distribution of CS from RNA-HDO was relatively less than that of AS (Figure 4), and most of the CS existed in the separated form, because nuclear intensities of CS from RNA-HDO showed no difference between mono- and dual-labeled groups (Figure 5E). On the contrary, the difference in the CS from 2′-OMe-HDO signals between mono- and dual-labeled groups was significantly large (Figure 5E). Therefore, we considered that most of the AS from 2′-OMe-HDO in the nucleus was in a wound form and then exerted much less potency there (Figure 5A; Figure S12B). At 24 h post-transfection, more AS from 2′-OMe-HDO accumulated in the nucleus than 2′-OM-CS and both strands did in lysosomes (Figure S14). This observation suggested a distinctive nature of HDO, in which AS was more efficiently transported to the nucleus than CS, even if CS was not cleaved.

### Decreased separation and nuclear transport in cleavage-resistant 2′-OMe-HDO

**Figure 6:**
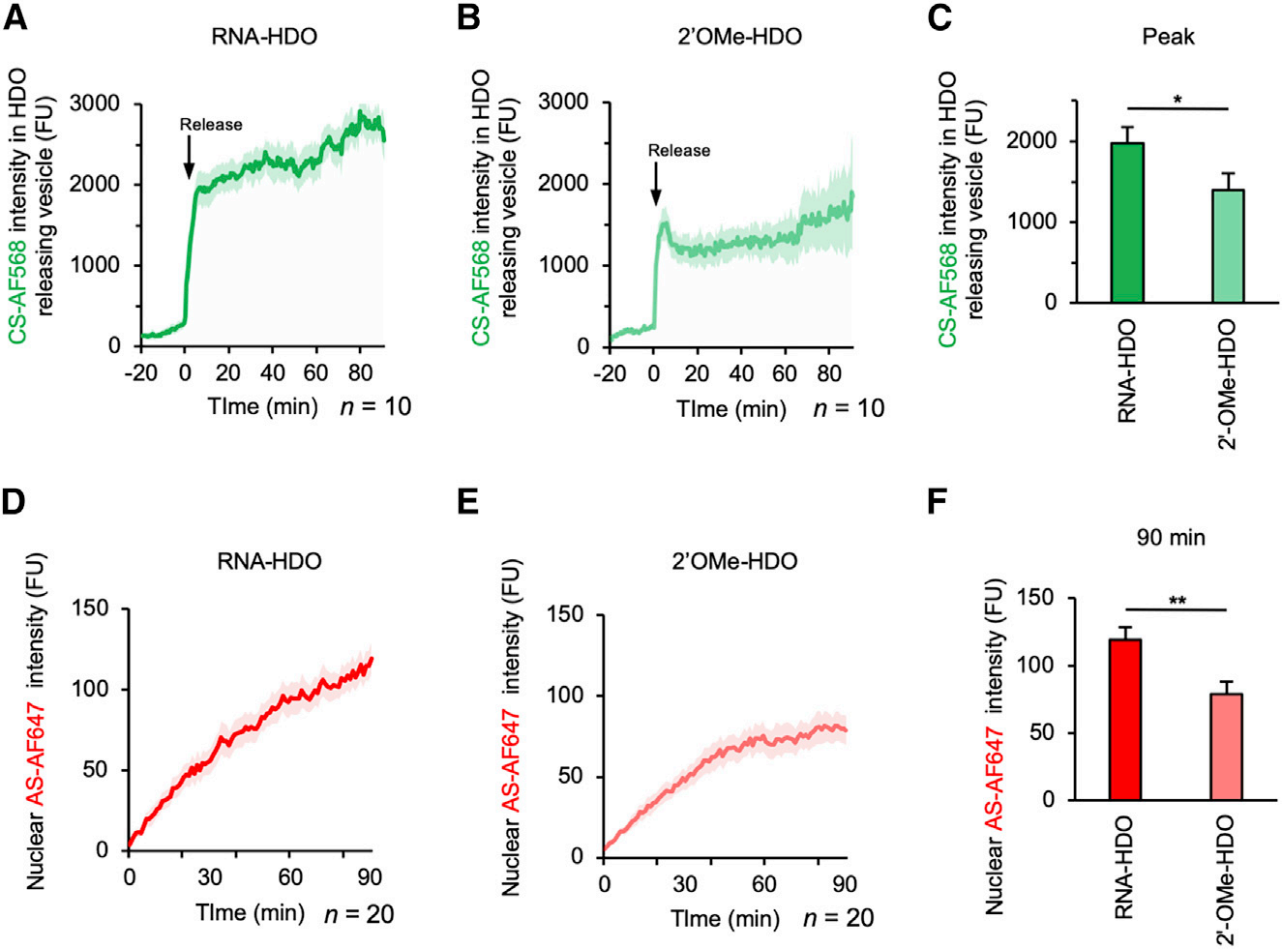
Decreased separation and nuclear transport in cleavage-resistant 2′-OMe-HDO Time-lapse imaging was performed after transfection with 50 nM RNA-HDO or 2′-OMe-HDO targeting intron *APOB*, where RNA or full 2′-OMe was labeled with AF568, and AS was labeled with AF647 to quantify the effects of CS cleavability in the early stage of HDO intracellular trafficking. Images were acquired just after transfection for 120 min, every 1 min. *t* = 0 was set just before the releases started. AF647 signals (red) were excited by a 646 nm laser and detected through a 700 (663–738) nm filter. AF568 signals (green) were excited by a 560 nm laser and detected through a 595 (570–620) nm filter. Images were obtained by weaker sensitivity settings than those of Figure 2 to maximize a slight difference of nuclear signals between RNA-HDO and 2′-OMe-HDO. (A and B) Sequential changes of CS-AF568 in HDO-releasing vesicles presented as absolute values normalized to background levels. (n = 10; ± SEM shown as shaded areas). (C) Mean intensities of CS-AF568 in HDO-releasing vesicles at peak levels (5.5 min after the releases) presented as absolute values normalized to background levels (∗p < 0.05; n = 10; mean ± SEM). (D and E) Sequential changes of AS-AF647 signals in nuclei presented as absolute values normalized to background levels. (n = 20; ± SEM shown as shaded areas). (F) Mean intensities of AS-AF647 in nuclei at 90 min after the releases presented as absolute values normalized to background levels (∗∗p < 0.01; n = 20; mean ± SEM).

Time-lapse imaging of RNA-HDO and 2′-OMe-HDO was performed (Figure 6) to quantify the effects of CS cleavage in the early stage of HDO intracellular trafficking. Similar to RNA-HDO (Figures 6A and 6D), HDO separation just after the cytosolic release and subsequent nuclear distribution were also observed in 2′-OMe-HDO (Figures 6B and 6E). However, comparison of their peak dequenching levels showed that cleavage-resistant 2′-OMe-HDO presented a lesser CS dequenching than RNA-HDO (Figure 6C). 2′-OMe-HDO also showed a lesser nuclear AS signal than RNA-HDO 90 min after the release (Figure 6F). We therefore derived that cleavage-independent separation and subsequent nuclear distribution existed with 50 nM 2′-OMe-HDO but was not sufficient for nuclear antisense activity.

## Discussion

### Distinctive intracellular trafficking of HDO

Here, we found a distinctive intracellular trafficking and processing mechanism of HDO, which is different from those of ASO or siRNA especially in two viewpoints: (1) separation-related rapid nuclear transport, and (2) cytosolic release from early endosomes. The results were reproduced using three other sequences and two cell lines. We also demonstrated the difference of intracellular trafficking between HDO and ASO by effective HDO versus non- or less-effective ASO (Figure 1, Figure 2, Figure 3; Figure S9), both highly effective HDO versus ASO (Figure S10), and non-target sequence (Figure S11). Furthermore, the equivalently transfected oligos presented the different effects and distributional patterns between HDO and AS (Figures S1, S2, and S8). Therefore, we concluded that HDO and ASO showed different intracellular behaviors, and these were not attributed to ineffective transfection or differences in the transfection efficiencies.

#### Separation-related rapid nuclear distribution

In this study, separation, release into the cytosol, and initiation of nuclear transport always occurred in this order and within a short period (median, 30 s). These strong relationships suggested that separation of HDO triggered its endosomal release and subsequent nuclear transport. Rapid release from endocytic vesicles has been previously visualized with other oligonucleotides.^21^^,^^24^^,^^26^ Lipid-formulated siRNA released from late endosomes into the cytosol has been demonstrated; however, the release is not triggered by the separation of double-strand RNA, and its nuclear distribution is either unobservable or transient.^21^^,^^24^ Therefore, separation-related rapid nuclear transport is the distinctive character of HDO. We additionally observed inflow signals from the HDO releasing endosomes in this study (Videos S2 and S3; Figure S7). A similar phenomenon has not been documented. Although the biological implication is unclear, this phenomenon is indicative of an unveiled intracellular structure, perhaps a “molecular tunnel” for DNA/RNA heteroduplex.

#### Cytosolic release from early endosomes

Independent of the delivery method (free uptake, ligand conjugation, or lipid formulation), oligonucleotides are mostly internalized by endocytosis and then trafficked through endosomal compartments,^9^^,^^10^ where their cytosolic release is an essential step to exert their gene silencing activity in the cytosol or nucleus.

The current study showed HDO was released from the vesicles which transiently expressed the early endosomal marker at the moment of release. Thereafter, the vesicles gradually expressed the late endosomal marker. In a previous study, Annexin 2 facilitates PS-ASO trafficking from early to late endosomes, where it may also contribute to PS-ASO release.^14^ Lipid-formulated siRNAs are released from the late endosome, which is functionally related to the cholesterol transport protein NPC1.^27^ HDO-binding protein has not been identified, but it might exist in the early endosome and mediate cytosolic release.

### Cleavage of CS and efficient gene silencing in the nucleus

Our experiments with 2′-OMe-HDO revealed that cleavage-independent separation existed (Figure 6), but it resulted in less nuclear distribution, where a larger amount of AS was in the wound form (Figure 5), and more HDO was needed for gene silencing (Figure S12B). Therefore, the cleavability of CS was associated with the efficient gene silencing in the nucleus.

In a previous study, we examined the cleavage site of CS *in vivo* using northern blotting and concluded that CS is cleaved by unidentified enzymes, most unlikely by RNase H1.^5^ As we discussed above, HDO separation in early endosomes triggered the cytosolic release and subsequent nuclear transport. Once CS was cleaved, melting temperature dramatically decreased, and separation would have been accelerated. Therefore, it is reasonable to consider that CS was cleaved in early endosomes by unidentified RNases. On the contrary, we cannot exclude the possibility that cleavage-independent separation of default RNA-HDO could also occur in early endosomes. A relatively low melting temperature of RNA-HDO compared to 2′-OMe-HDO might support this idea. The identification of binding and cleaving proteins of HDO and elucidation of its cleaving mechanism are needed, which would aid in designing more effective drugs, by balancing the extracellular RNase-resistance and intracellular cleavability.

### Innate DNA/RNA biology

We showed that the exogenously administered short DNA/RNA heteroduplex had a distinctive trafficking pathway from early endosomes into the nucleus via cytosol. Regarding the exogenous origin, pathogen-derived RNA/DNA heteroduplex has been well investigated, and it induces interferon-related immune response via nucleotide sensor proteins like Toll-like receptor 9 (TLR9).^28^^,^^29^ On the contrary, endogenous DNA/RNA heteroduplex has been mainly described in nuclear functions such as RNA primer of Okazaki fragments,^30^^,^^31^ ribonucleotide incorporation^32^ during DNA replication, R-loops,^33^ and G-quadruplexes^34^ during transcription. Recently, several studies proposed its extra-nuclear function. DNA/RNA heteroduplex of R-loop structure in the nucleus is cleaved and exported to cytosol,^35^^,^^36^ where the heteroduplex binds to Ago2 and regulates miRNA,^35^ or single-stranded DNA functions as natural antisense after RNA cleavage by RNase H and TREX1.^36^ Formulation of DNA and RNA into extracellular vesicles was also reported in cancer cells.^37^ Although we must be cautious not to mix our findings obtained using the modified oligonucleotides, with innate biology, our findings of intracellular trafficking of HDO might help to understand innate biology of the DNA/RNA heteroduplex.

### Limitations

The results of our study are primarily based on lipid transfection experiments. We observed that gymnosis (free uptake) did not induce an effective delivery in our experimental system (Figure S4). The observations of our study need to be generalized and applied to *in vivo* studies in the future for advancing to therapeutic application.

### Conclusion

Here, we presented the intracellular trafficking of HDO. Understanding this unique mechanism will help us design more efficient drugs and also provide more insight into innate DNA/RNA cellular biology.

## Materials and methods

### Design and synthesis of oligonucleotides

A series of oligonucleotides were synthesized by Gene Design (Osaka, Japan). The sequences targeting intron *APOB* mRNA were as follows: ASO, 5′-c∗a∗t∗c∗c∗c∗a∗c∗c∗a∗c∗a∗t∗a∗g∗c-3′; cRNA, 5′-G∗C∗U∗AUGUGGUGGG∗A∗U∗G-3′; full 2′-OMe CS, 5′-G∗C∗U∗AUGUGGUGGG∗A∗U∗G-3′. Lower case letters represent DNA, lower case underlined letters represent LNA (c denotes LNA 5-methylcytosine), upper case letters represent RNA, upper case underlined letters represent 2′-*O*-methyl sugar modification, and ∗ indicates phosphorothioate linkage. The other sequences used were as follows: exon *APOB*: AS, 5′-g∗c∗a∗t∗t∗g∗g∗t∗a∗t∗t∗c∗a-3′; CS, 5′-U∗G∗A∗AUACCAAU∗G∗C-3′.^38^ *SNCA-1*: AS, 5′-c∗c∗a∗t∗t∗c∗c∗c∗a∗a∗g∗a∗g∗a∗c∗c∗c∗a∗g∗a-3′; CS, 5′-U∗C∗U∗G∗G∗GUCUCUUGGG∗A∗A∗U∗G∗G-3′. *SNCA-2*: AS, 5′-a∗g∗a∗a∗g∗a∗a∗t∗c∗a∗a∗t∗t∗g∗c∗t∗t∗t∗a∗c-3′; CS, 5′-G∗U∗A∗A∗A∗GCAAUUGAUU∗C∗U∗U∗C∗U-3′.^39^ Scramble sequence (no target): AS, 5′-g∗g∗c∗c∗a∗a∗t∗a∗c∗g∗c∗c∗g∗t∗c∗a-3′; CS, 5′-U∗G∗A∗ CGGCGUAUUG∗G∗C∗C-3′.^40^ siRNA targeting *GAPDH*: sense, GUAUGACAACAGCCUCAAGtt; antisense, CUUGAGGCUGUUGUCAUACtt.^41^ Full 2′-OMe CS targeting mouse *Malat*: G∗C∗A∗UUCAGUGAAC∗U∗A∗G. Alexa Fluor 488, Alexa Fluor 568, or Alexa Fluor 647 was covalently bound to the 5′ end of antisense or 3′ end of the CS, as mentioned in the text. Equimolar concentrations of antisense and complementary strands in nuclease-free water (Life Technologies, Carlsbad, CA, USA) were heated at 95°C for 5 min and annealed at room temperature (20°C–25°C) for over 1 h to generate HDO.

### Cell Transfection

Huh-7 cells, cultured in DMEM containing 10% fetal bovine serum (FBS) and 1% penicillin/streptomycin (P/S) at 37°C and 5% CO_2_, were seeded in 24-well plates at 25,000–50,000 cells/well (depending on the time frame of the assay) 12 h before transfection. Oligonucleotides in 100 μL Opti-MEM (Life Technologies) and 1 μL Lipofectamine RNAiMAX (Life Technologies) were mixed at room temperature for 20 min and then added to cells in 400 μL DMEM (FBS+ P/S−) and incubated for 4 h. Transfection medium was then replaced with fresh DMEM (FBS+ P/S+) for subsequent experiments.

In co-transfection experiments, two kinds of oligonucleotides were mixed initially in Opti-MEM, to which 1 μL Lipofectamine RNAiMAX was added. In gymnotic delivery experiments, oligonucleotides in 100 μL Opti-MEM were added to cells in 400 μL DMEM (FBS+ P/S−) and incubated for 24 h. We could not evaluate the gymnotic delivery longer than 24 h because cells died. Additionally, *ACTB* was used as the internal control in this experiment because we were afraid that a large number of oligos affected the expression of *GAPDH*. HEK293T cells were utilized only for the experiments presented in Figures S9 and S10.

### Quantitative real-time PCR assay

Total RNA was extracted with Isogen II (Nippon Gene, Tokyo, Japan) and was reverse-transcribed with PrimeScript RT Master Mix (Takara Bio, Kusatsu, Japan). The cDNAs were amplified using the LightCycler 480 II (Roche Diagnostics, Rotkreuz, Switzerland). Gene expression values were calculated using the comparative delta *C*t method, normalized by the expression of the housekeeping gene, *GAPDH* or *ACTB*, as previously described.^5^ TaqMan primers (Applied Biosystems, Life Technologies) used in this study were as follows: *APOB* (Hs00181142_m1), *GAPDH* (Hs99999905_m1), *SNCA* Hs01103383_m1), and *ACTB* (forward: 5′-CGGACTATGACTTAGTTGCGTTACA-3′; reverse: 5′-GCCATGCCAATCTCATCTTGT-3′; probe: 5′-FAM-CCTTTCTTGACAAAACCTAACTTGCGCAGA-TAMRA-3′).

### Live-cell imaging

For live-cell imaging, Huh-7 cells were seeded in 35 mm Cell Imaging Dishes (Eppendorf, Hamburg, Germany) at 12,500 cells/well 12 h before transfection or transduction of labeled endosomes. In endosomal co-localization experiments, cells were transduced with GFP-labeled early or late endosomes 24 h before their transfection with oligonucleotides, using BacMam 2.0 CellLight Early endosome-GFP or Late endosome-GFP (Life Technologies), respectively, as previously described.^42^ Cells were treated with LysoTracker Red DND-99 (Life Technologies) 1 h before the imaging, according to the manufacturer’s instructions, to visualize lysosomes.

Transfection mixture containing dye-conjugated oligonucleotides and 1 μL Lipofectamine RNAiMAX in 100 μL Opti-MEM was prepared and stored at room temperature for 20 min. One minute after culture medium was replaced with the transfection mixture and 400 μL phenol red-free DMEM (FBS+ P/S−) (FluoroBrite, Life Technologies) on the stage of the microscope, time-lapse imaging was initiated. The medium was replaced with phenol red-free DMEM (FBS+ P/S+) 4 h after transfection, depending on the time frame of experiments.

### Microscope settings

All images in this study were obtained using a live-cell imaging system (Nikon A1R laser scanning confocal microscopy, Nikon NIS elements AR software ver. 4.5, Tokyo, Japan) and stage top incubator (INUG2H-TIZSH, Tokai Hit, Fujinomiya, Japan). Instruments were warmed up, and cells and medium were incubated in the chamber for more than 1 h before imaging to stabilize the conditions in the chamber (37°C, 5% CO_2_, and 100% humidity).

A preliminary experiment determined that the thickness of Huh-7 cells was less than 7 μm by *z*-section imaging at high magnitude. Therefore, the microscopic settings were adjusted accordingly to enable the detection of all intracellular signals. In this setting (10× objective and 10 ocular lenses; pinhole 28 μm), the calculated thickness of optical sections was 11 μm, and time-lapse imaging was obtained at a single plane, which was focused using differential interference contrast (DIC) imaging at initiation and autoregulated by Nikon Perfect Focus System. Depending on each experiment, 3 × 3 to 4 × 4 tailing images of each fluoresce and DIC were acquired every 30–120 s for 120 min. Imaging parameters, like laser intensity and exposure time, were uniformly set in the same experiments.

### Measuring imaging data

The fluorescence intensity of HDO in releasing vesicles, nucleus, and cytosol were analyzed using Nikon NIS elements AR software ver. 4.5 (Nikon). As mentioned earlier, HDO release was defined as the sudden increase of cytosolic AS signal, and a releasing vesicle was identified by a transient increase of AF647 signal in the vesicle (except AF488 signal in Figure S6C), which meant the cancellation of self-quenching.^24^ We also defined the uniform and well-demarcated nuclear signals as nuclear transport and counted them accordingly.

Nuclear and plasma membranes were identified with DIC imaging and manually traced, blinded by other fluorescent signals. Lysosomes were identified when lysotracker signals localized. Intensity values were presented as absolute values, differences between actual signals and background levels, to compare the signals from the same dye.

In time-lapse imaging, the vesicles that released HDO first in individual cells were traced and mean signal intensity of each fluorophore was obtained. Relative values were calculated as previously described.^24^ Namely, *t* = 0 was set just before the nuclear distribution started. Each fluorescence intensity was normalized to 0–1, with 0 being the background intensity and 1 being the highest intensity value for each object, in order to adjust various expression levels of each object. Here, we defined the background level as the mean intensity of an arbitrary no-cell area in the same image at the given time,^21^^,^^22^ instead of the mean intensity of untransfected cells,^24^ because we needed to eliminate the fluorescence caused by the dye-containing medium (note that the optical section was as thick as 11 μm in our settings). Exceptionally, in GFP-labeled early or late endosome experiments, cytosolic regions without the target protein expression were used as background.

### RNase treatment assay

Various oligonucleotides (40 pmol), of which AS and CS were labeled with AF647 and AF488, respectively, were incubated with 1 ng of RNase A (QIAGEN, Hilden, Germany) or 10 units of RNase H (Takara Bio) at 37°C for 1 h and then loaded on 20% polyacrylamide gel. After electrophoresis in TBE, fluorescence of each dye was imaged using ChemiDoc Touch MP (Bio-Rad, Hercules, CA, USA).

### Statistical Analysis

All experiments were performed at least three times. All data represent mean ± SEM, unless otherwise mentioned. Student’s t tests were used to compare results obtained from different groups and/or conditions using Microsoft Excel. A p value of less than 0.05 was considered significantly significant.

## Supplemental information

Document S1. Figures S1–S14

Document S2. Article plus supplemental information
