# Supplementary material for: Separation-related rapid nuclear transport of DNA/RNA heteroduplex oligonucleotide: unveiling distinctive intracellular trafficking
Source: Mol Ther Nucleic Acids. 2020 Dec 3;23:1360–70. doi: 10.1016/j.omtn.2020.11.022 (PMC7933600; doi:10.1016/j.omtn.2020.11.022)
Supplement: Document S1. Figures S1–S14 [file mmc1.pdf]

OMTN, Volume *23*

Supplemental information

Separation-related rapid nuclear transport of DNA/RNA heteroduplex oligonucleotide: unveiling distinctive intracellular trafficking

Daisuke Ono, Ken Asada, Daishi Yui, Fumika Sakaue, Kotaro Yoshioka, Tetsuya Nagata, and Takanori Yokota

Supplemental figures and legends

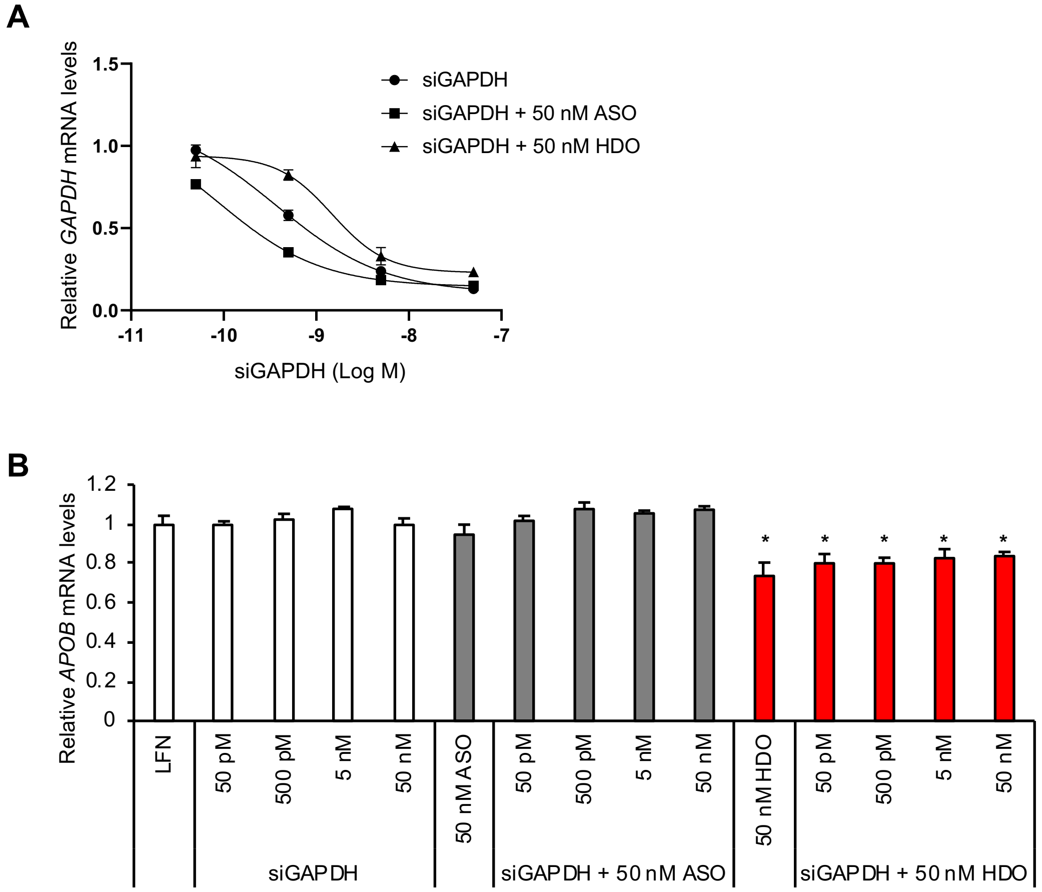

Figure S1. Co-Transfection of ASO/HDO with siGAPDH to Control the Transfection Efficiency of ASO and HDO
To evaluate and control transfection efficiency of different oligonucleotides, 50 nM ASO or HDO targeting intron *APOB* were co-transfected with various doses of control siRNA targeting a housekeeping gene; *GAPDH*. 24 h after transfection, *APOB* and *GAPDH* mRNA levels were quantified using RT-qPCR, normalized to those of *ACTB* and "no siGAPDH" control in each group. In the experiments, we aimed to calculate 50% inhibitory concentration (IC_{50}) of siRNA, at which, we could compare the intracellular efficacy of co-transfected ASO and HDO by equalizing co-transfected siGAPDH activity. (A) The inhibitory dose-responsive curve was fitted with the *GAPDH* mRNA levels at various doses of siGAPDH. IC_{50} of single transfection with siGAPDH, co-transfection of siGAPDH with 50 nM ASO, and 50 nM HDO were calculated as 370 pM, 88 pM, and 1.4 nM, respectively. (B) RT-qPCR analysis of *APOB* levels 24 h after co-transfection. At around IC_{50} of siGAPDH (50 pM ~ 5 nM), comparable doses of HDO significantly downregulated target *APOB* mRNA, whereas same doses of ASO did not. Note that Figures S1A and S1B were based on data from the simultaneous experiment (* $p < 0.05$, vs lipofectamine (LFN) control; n = 3; mean ± SEM).

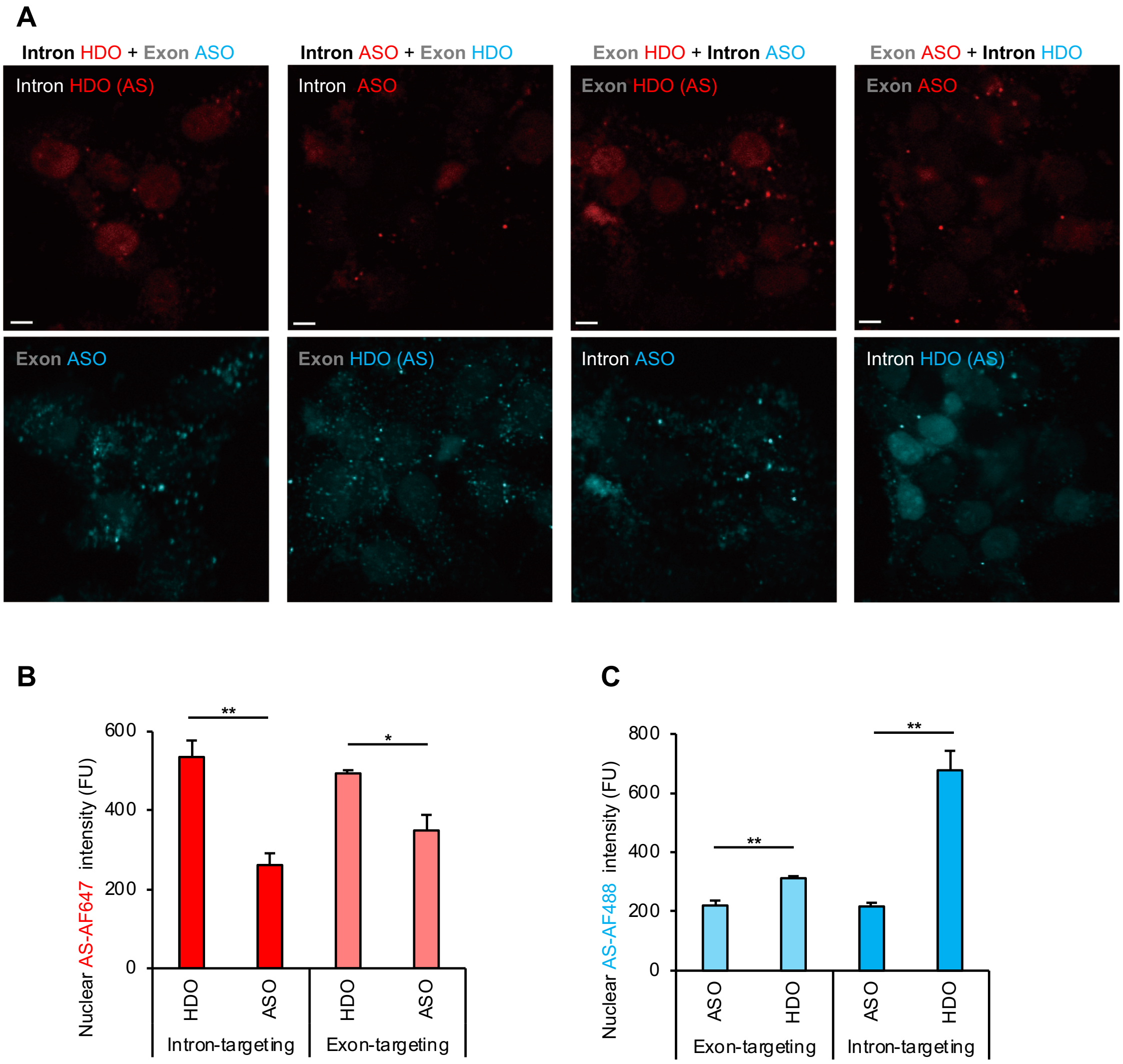

Figure S2. Co-Transfection with ASO and HDO

To compare the nuclear distribution equalizing transfection amount, we performed co-transfection with dye-labeled ASO and HDO, and imaged them 24 h after transfection, where we used two antisense sequence (16-mer targeting intron *APOB* and 13-mer targeting exon *APOB*) so that complementary strand (CS) of HDO did not hybridize with the other ASO. We labeled antisense strand (AS) of HDO and ASO with AF647 and AF488. These two sequences for HDO or ASO, and their labels were switched to control the effects of sequences and dyes. (A) Representative images 24 h after co-transfection of 50 nM ASO and HDO. AF-647 signals (red) were excited by a 646 nm laser and detected through a 700 (663-738) nm filter. AF-488 signals (cyan) were excited by a 488 nm laser and detected through a 525 (500-550) nm filter. Bar = 10 µm. (B) Mean nuclear intensities presented as absolute values normalized to background levels. More signals of HDO were detected in nuclei than those of ASO after controlling effects of sequences and dyes (* $p < 0.05$, ** $p < 0.01$; n = 3 images for each 50 cells; mean ± SEM).

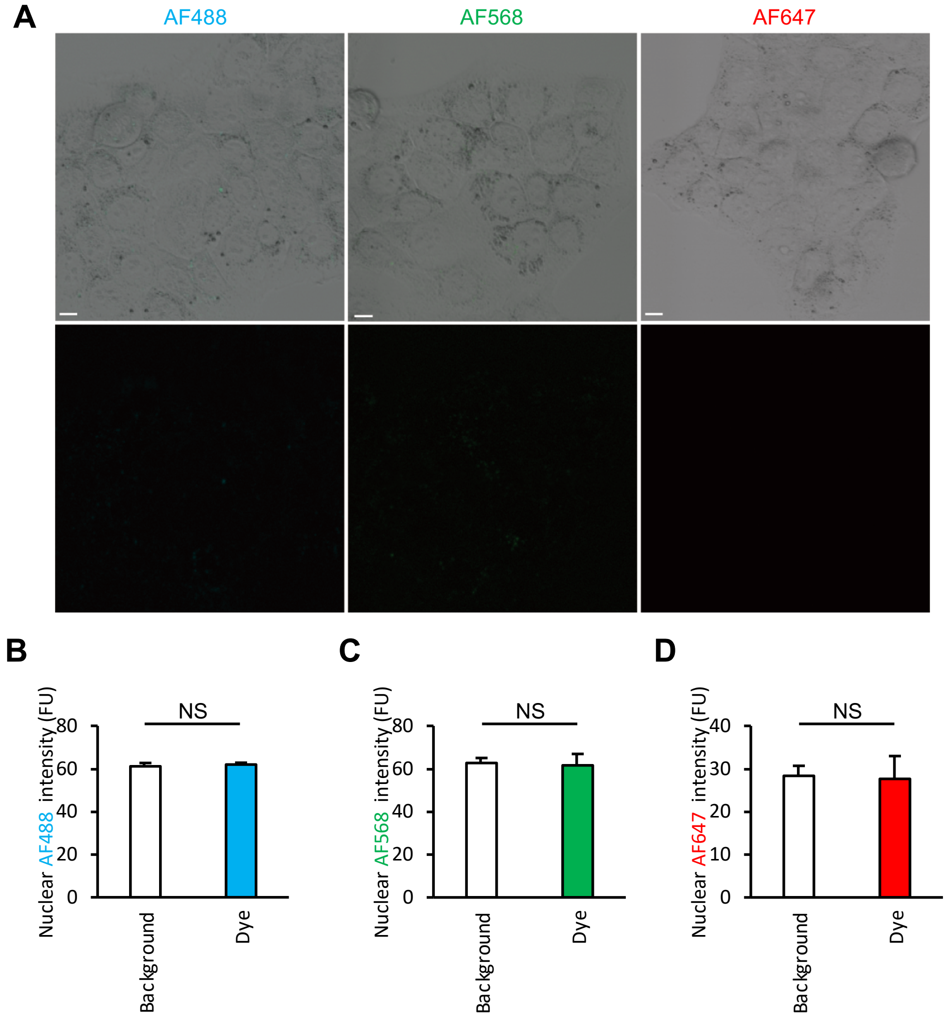

Figure S3. Transfection of Dyes

(A) Representative images 24 h after transfection with 50 nM of each dye. The upper row shows merged images of differential interference contrast (DIC) and each fluorescent signal (left, AF488; center, AF568; right, AF647). The lower row shows each fluorescence image. AF488 signals (cyan) were excited by a 488 nm laser and detected through a 525 (500-550) nm filter. AF568 signals (green) were excited by a 560 nm laser and detected through a 595 (570-620) nm filter. AF647 signals (red) were excited by a 646 nm laser and detected through a 700 (663-738) nm filter. Bar = 10 µm. (B) Mean nuclear intensities of dyes and background levels presented as absolute values (NS, not significant; n = 3 images for every 50 cells; mean ± SEM).

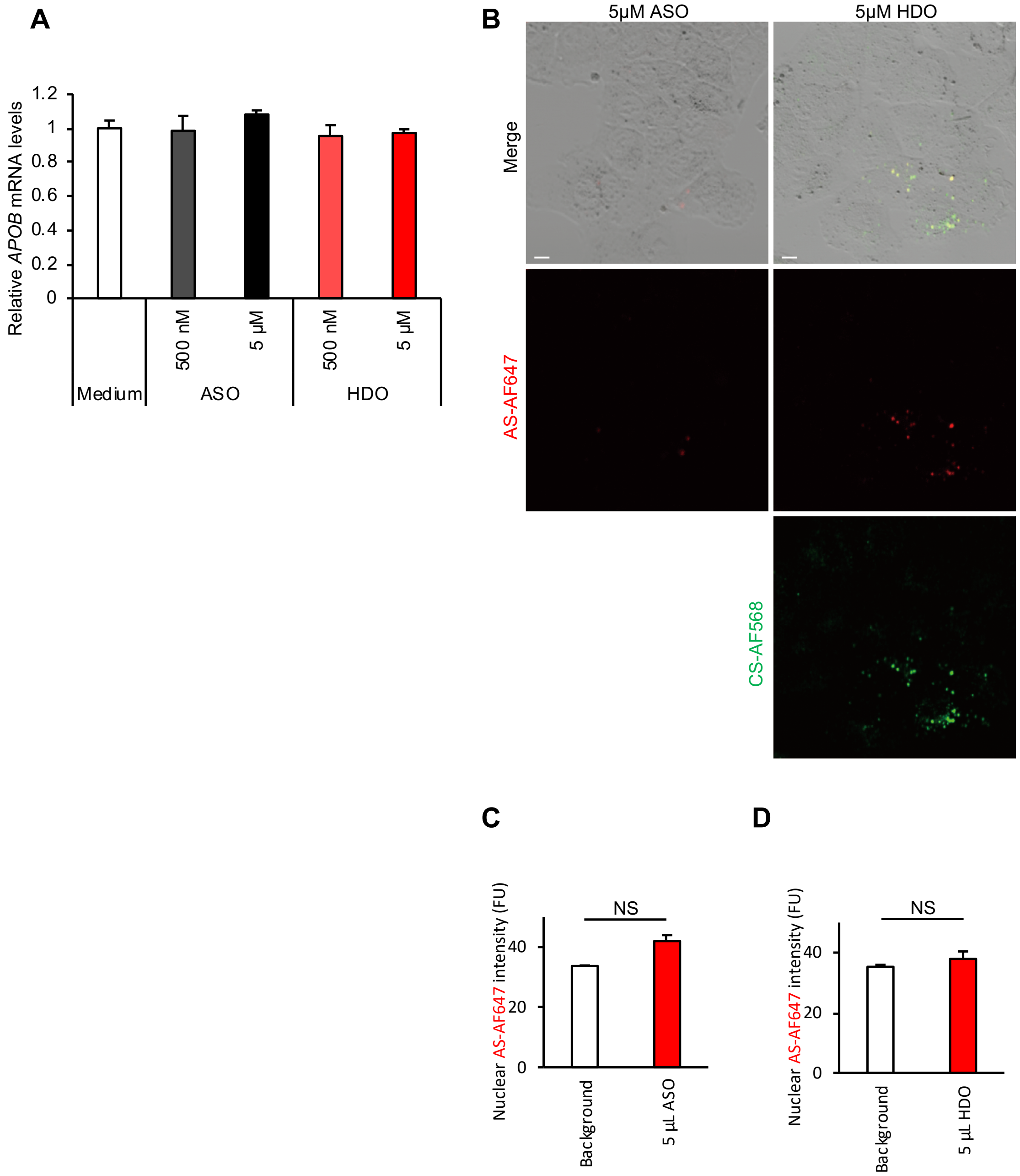

Figure S4. Gymnotic Delivery of ASO and HDO
To evaluate other delivery method than lipid transfection, we performed gymnotic (free uptake) delivery. (A) RT-qPCR analysis of *APOB* levels, normalized to *ACTB* levels 24 h after gymnotic delivery with ASO or HDO targeting intron *APOB* (no significance was found vs control medium; n = 3; mean ± SEM). (B) Representative images 24 h after transfection with 5 µM of ASO or HDO targeting intron *APOB*. Only 1% of oligos were labeled to avoid the non-specific hyperintensity of the background signal. AF647 signals (red) were excited by a 646 nm laser and detected through a 700 (663-738) nm filter. AF568 signals (green) were excited by a 560 nm laser and detected through a 595 (570-620) nm filter. Bar = 10 µm. (C, D) Mean nuclear intensities of AS-AF647 with 5 µM of ASO (C) and HDO (D) presented as absolute values (no significance (NS) was found vs background levels; n = 3 images for every 50 cells; mean ± SEM).

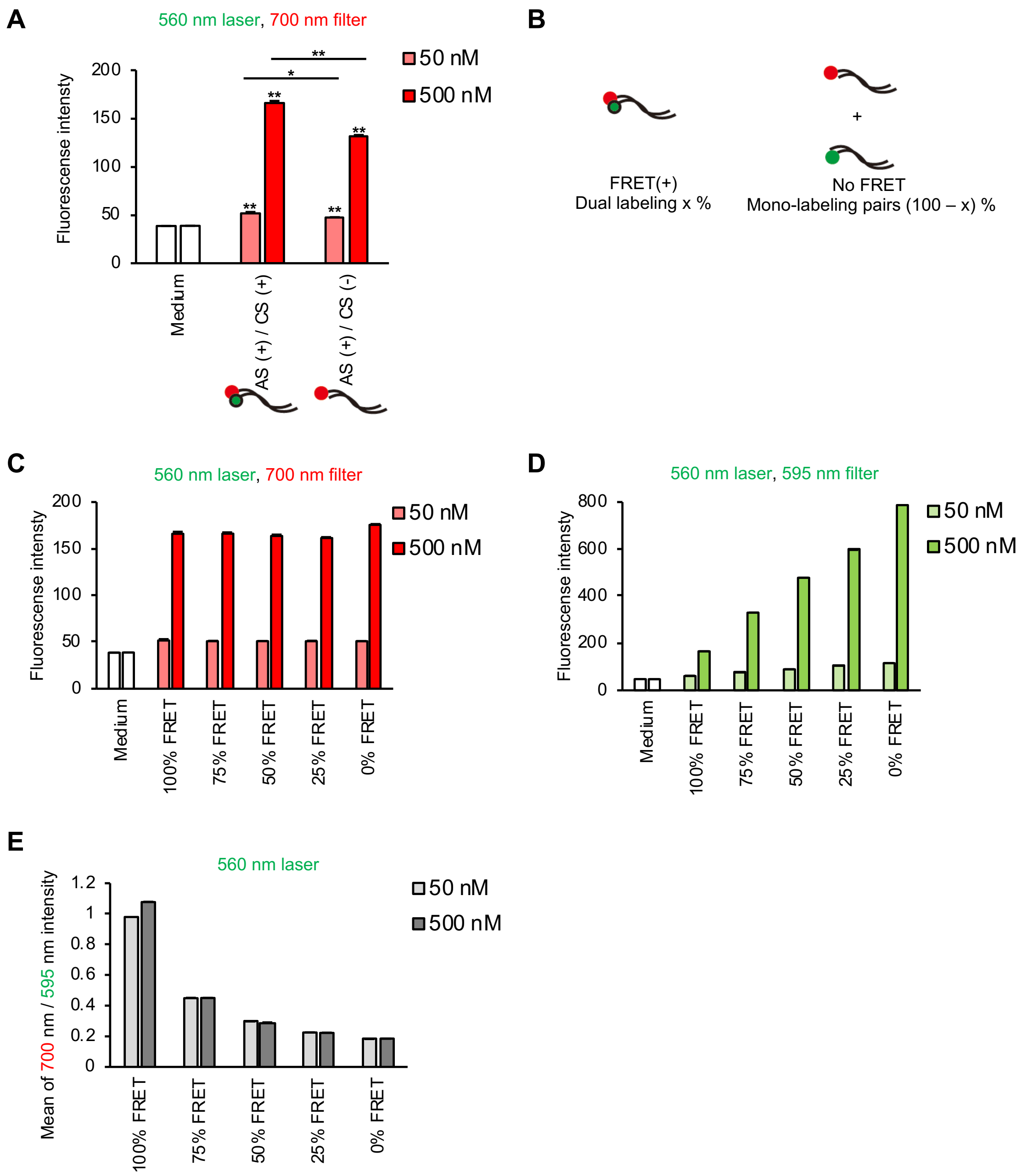

Figure S5. Confirmation of FRET System

To confirm the FRET system which we utilized in the experiments, we measured the fluorescence intensity of dye-conjugated HDO in solution without cell or transfection reagent. (A) 50 nM or 500 nM HDO targeting intron *APOB*, composed of AF647-labeled antisense strand (AS)/AF568-labeled complementary strand (CS) [AS (+) / CS(+)] or AF647-labeled AS/CS without dye [AS (+) / CS(-)], were excited by a 560 nm laser, detected through a 700 (663-738) nm filter (* $p < 0.05$, ** $p < 0.01$ vs medium control if not indicated by bars; n = 3; mean absolute value ± SEM). In this setting, the difference between AS (+)/CS(+) and AS (+)/CS(-) means FRET signal. The difference between AS (+)/CS(-) and medium means non-specific AF-647 signal excited by a 560 nm laser, which was more intense than FRET signals, and made it difficult to isolate FRET signals. (B-E) In the HDO separation site, where winding HDO and separated AS-AF647 exist densely, we could not directly identify acceptor signal of FRET from non-specific signals of isolated AF647 excited by 560 nm laser. To solve this problem, we mixed FRET (+) dual labeled HDO and no FRET single-labeled pairs of HDO at various rates, and measured solution intensity, which models the winding and/or separated form of double-stranded oligos[21] (n = 3; mean absolute value ± SEM). Signals excited by a 560 nm laser, through a 700 (663-738) nm filter were not remarkably different between FRET (+) model and no FRET model (C). On the contrary, a remarkable increase of the signal excited by a 560 nm laser and detected through a 595 (570-620) nm filter was observed with an increase of no FRET mono-labeling pairs, which corresponded to the dequenching signal of the donor dyes (D). To control the difference in concentration, we also calculated the ratio[21]—the signal intensity from the 700 nm filter divided by that from 595 nm filter—both of which were excited by a 560 nm laser and normalized to medium intensity (E). This ratio was correlated to FRET regardless of HDO concentration.

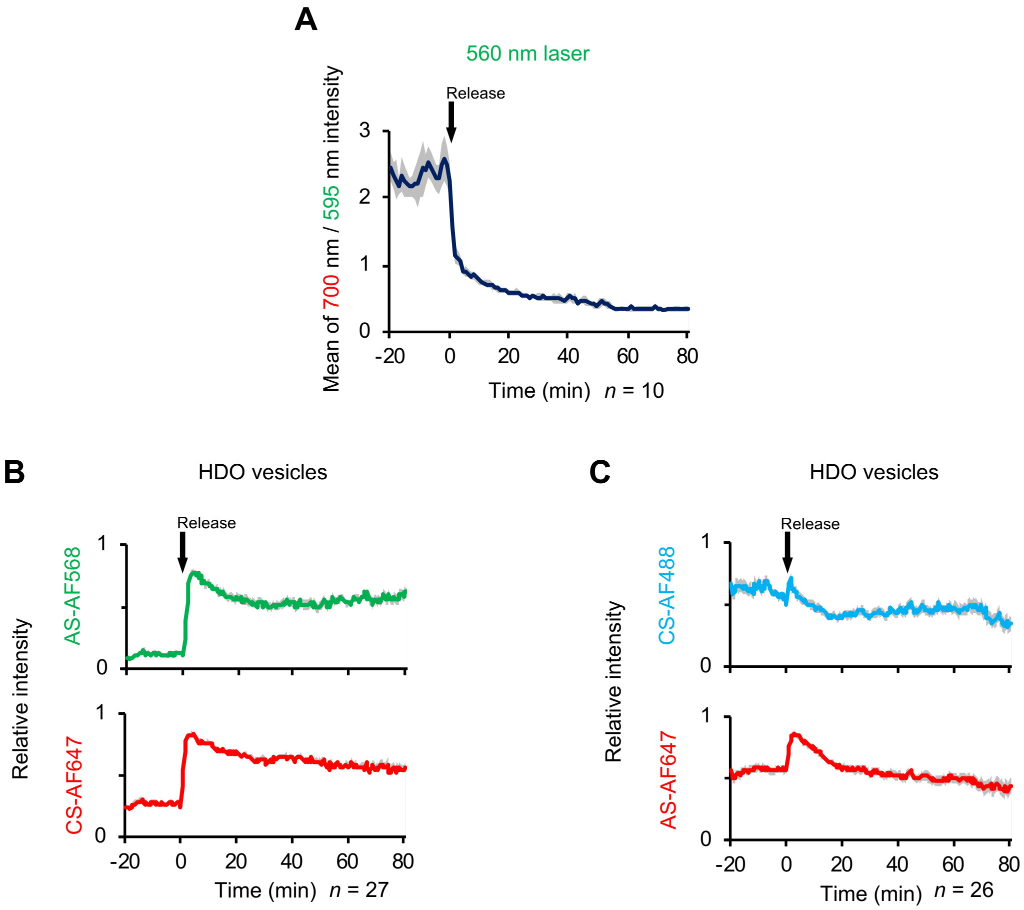

Figure S6. Confirmation of HDO Separation in HDO-Releasing Vesicles

(A) To detect FRET, signals through 595 (570-620) nm and 700 (663-738) nm filters excited by a 560 nm laser in HDO-releasing vesicles were measured after transfection with 50 nM HDO targeting intron *APOB*, composed of antisense strand (AS)-AF647 and complementary strand (CS)-AF568. The ratio—the mean signal intensity from the 700 nm filter divided by that from 595 nm filter—was sequentially calculated, as presented in Figure S5. Sudden decrease of the ratio at the time of the release was confirmed to be the cancellation of the FRET signals. (B) To exclude the possibility that these signal changes were not as a result of a particular dye-nucleotide interaction, AS was reversely labeled with AF568 and CS with AF647. AF647 signals (red) were excited by a 646 nm laser and detected through a 700 (663-738) nm filter. AF568 signals (green) were excited by a 560 nm laser and detected through a 595 (570-620) nm filter. Dequenching signals of AS-AF568 and cancellation of self-quenching of CS-AF647 were observed just after the time of the release. (C) To confirm that FRET does not occur in AF647 and AF488 pairs and evaluate cancellation of self-quenching, AS was labeled with AF647 and CS with AF488. AF647 signals (red) were excited by a 646 nm laser and detected through a 700 (663-738) nm filter. AF488 signals (in cyan) were excited by a 488 nm laser and detected through a 525 (500-550) nm filter. Sequential signal changes of HDO-releasing vesicles are presented. Images were taken just after transfection with 50 nM HDO, every 1 min (A), or 30 sec (B, C). $t = 0$ is set just before the release started. (n = 10 (B), 27 (B), 26 (C); ± SEM, shaded areas). Results were pooled from three experiments per condition (B, C).

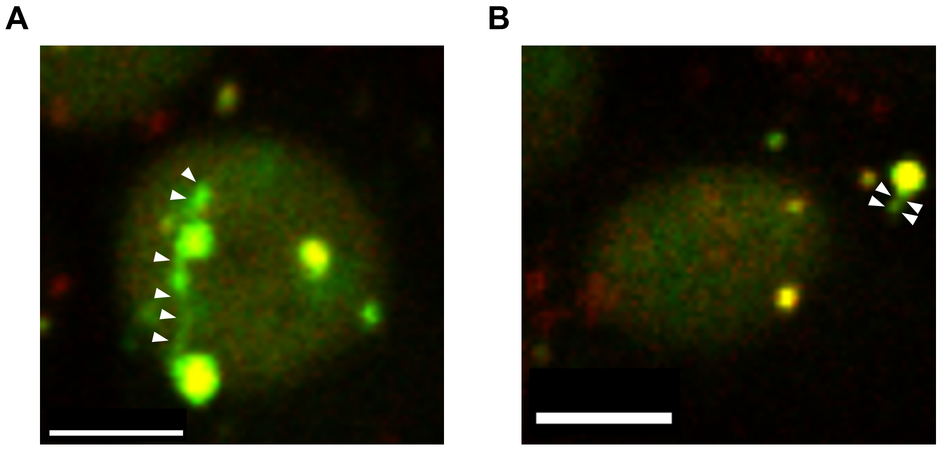

Figure S7. Inflow Signals from HDO-Releasing Vesicles

(A, B) Representative figures of inflow signals (arrowheads) from HDO-releasing vesicles in Movie 2A, B, respectively. Images were taken in a high sensitivity setting compared to the experiments in Figure 2, just after transfection with 50 nM HDO targeting intron *APOB*, composed of antisense strand-AF647 (red) and complementary strand-AF568 (green). AF647 signals were excited by a 646 nm laser and detected through a 700 (663-738) nm filter. AF568 signals were excited by a 560 nm laser and detected through a 595 (570-620) nm filter. Bar = 10 µm.

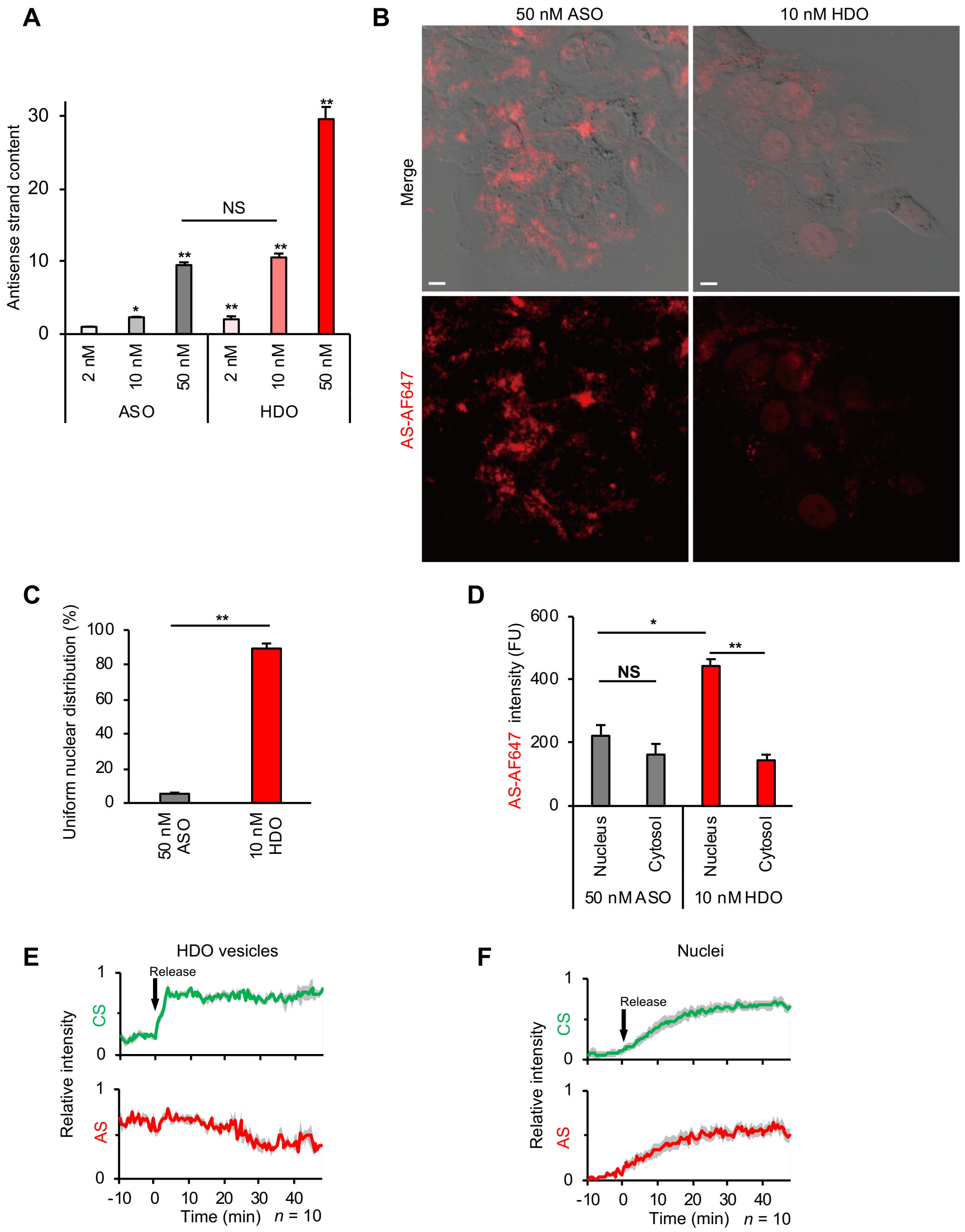

Figure S8. Evaluation and Equalization of Transfection Efficiency of HDO and ASO
To evaluate transfection efficiency of ASO and HDO, we measured antisense strand levels in cells, 24 h after transfection with various doses of ASO or HDO targeting intron *APOB*, and then determined the comparable dose of HDO to that of ASO as shown in Figures 1, 2. (A) RT-qPCR analysis of antisense strand content normalized to the levels of *U6* RNA 24 h after transfection with various doses of ASO or HDO, targeting intron *APOB* mRNA (* p < 0.05, ** p < 0.01 vs 2nM ASO; n = 3; mean ± SEM). The results showed that 10 nM HDO was comparable to 50 nM ASO. At these doses, 10 nM HDO significantly downregulated *APOB* mRNA but 50 nM ASO did not (Figure 1B). (B) Representative images 24 h after transfection with 50 nM ASO and 10 nM HDO. The upper row shows merged images of differential interference contrast (DIC) and antisense strand (AS)-AF647 (red). The lower row shows AS-AF64. AF647 signals were excited by a 646 nm laser and detected through a 700 (663-738) nm filter. Bar = 10 µm. (C) Percentage of cells with uniform nuclear distribution 24 h after transfection with 50 nM ASO or 10 nM HDO (** p < 0.01; n = 3 images for every 50 cells). (D) Mean intensities of AS-AF647 in the nucleus or cytosol 24 h after transfection with 50 nM ASO or 10 nM HDO, presented as absolute values normalized to the background signal. (* p < 0.05, ** p < 0.01; NS, not significant; n = 3 images for every 50 cells). (E, F) Time-lapse images were taken every 30 seconds after transfection with 10 nM HDO, and sequential signal changes of AS-AF647 (red) and complementary strand (CS)-AF568 (green) in HDO-releasing vesicles (E) and nuclei (F) were measured. Separation-related nuclear distribution of both strands just after cytosolic release was observed in 10 nM HDO similar to 50 nM. AF647 signals were excited by a 646 nm laser and detected through a 700 (663-738) nm filter. AF568 signals were excited by a 560 nm laser and detected through a 595 (570-620) nm filter. *t* = 0 is set just before the releases started. Mean intensities of each region were presented as relative values (0-1), with 0 being the background intensity, and 1 being the highest intensity value for each object (n = 10; ± SEM, shaded areas).

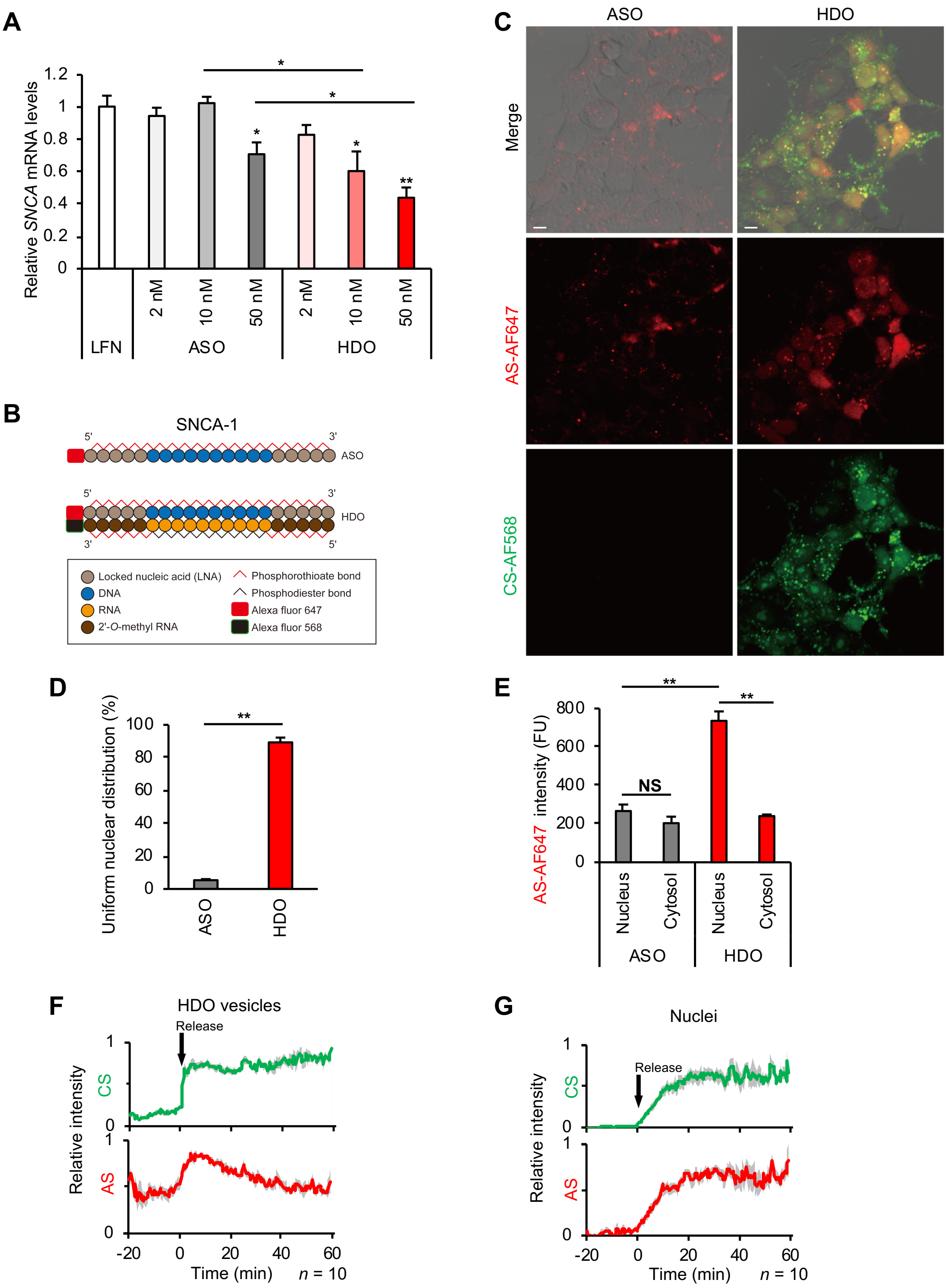

Figure S9. Separation-Related Nuclear Distribution of HDO by SNCA-1 Sequence in HEK 293T Cells

To confirm the separation-related nuclear distribution of HDO by another target, we performed the same experiments as shown in Figures 1, 2, by transfecting with ASO or HDO targeting intron region of *SNCA* (Alpha-Synuclein). Because *SNCA* was not expressed in Huh 7 cells, HEK 293T cells were transfected with Lipofectamine RNAiMAX. (A) RT-qPCR analysis of relative *SNCA* mRNA levels 24 h after transfection, normalized to *GAPDH* (* $p < 0.05$, ** $p < 0.01$ vs. lipofectamine (LFN) control; n = 3; mean ± SEM). (B) Designs of dye-conjugated ASO and HDO, targeting intron region of *SNCA* (SNCA-1 sequence), where complementary strand (CS) was labeled with AF568, and antisense strand (AS) was labeled with AF647. (C) Representative images 24 h after transfection with 50 nM ASO and HDO. The upper row shows merged images of differential interference contrast (DIC), AS-AF647 (red) and CS-AF568 (green). AF647 signals were excited by a 646 nm laser and detected through a 700 (663-738) nm filter. AF568 signals were excited by a 560 nm laser and detected through a 595 (570-620) nm filter. Bar = 10 µm. (D) Percentage of cells with uniform nuclear distribution 24 h after transfection with 50 nM ASO or HDO (** $p < 0.01$; n = 3 images for every 50 cells). (E) Mean intensities of AS-AF647 in the nucleus or cytosol 24 h after transfection with 50 nM ASO or HDO, presented as absolute values normalized to the background signal. (** $p < 0.01$; NS, not significant; n = 3 images for every 50 cells). (F, G) Time-lapse images were taken every 30 seconds after transfection with 50 nM HDO, and sequential changes of AS-AF647 and CS-AF568 signals in HDO-releasing vesicles (F) and nuclei (G) were measured. Separation-related nuclear distribution of both strands just after cytosolic release was observed. AF647 signals were excited by a 646 nm laser and detected through a 700 (663-738) nm filter. AF568 signals were excited by a 560 nm laser and detected through a 595 (570-620) nm filter. *t* = 0 is set just before the releases started. Mean intensities of each region were presented as relative values (0-1), with 0 being the background intensity, and 1 being the highest intensity value for each object (n = 10; ± SEM, shaded areas).

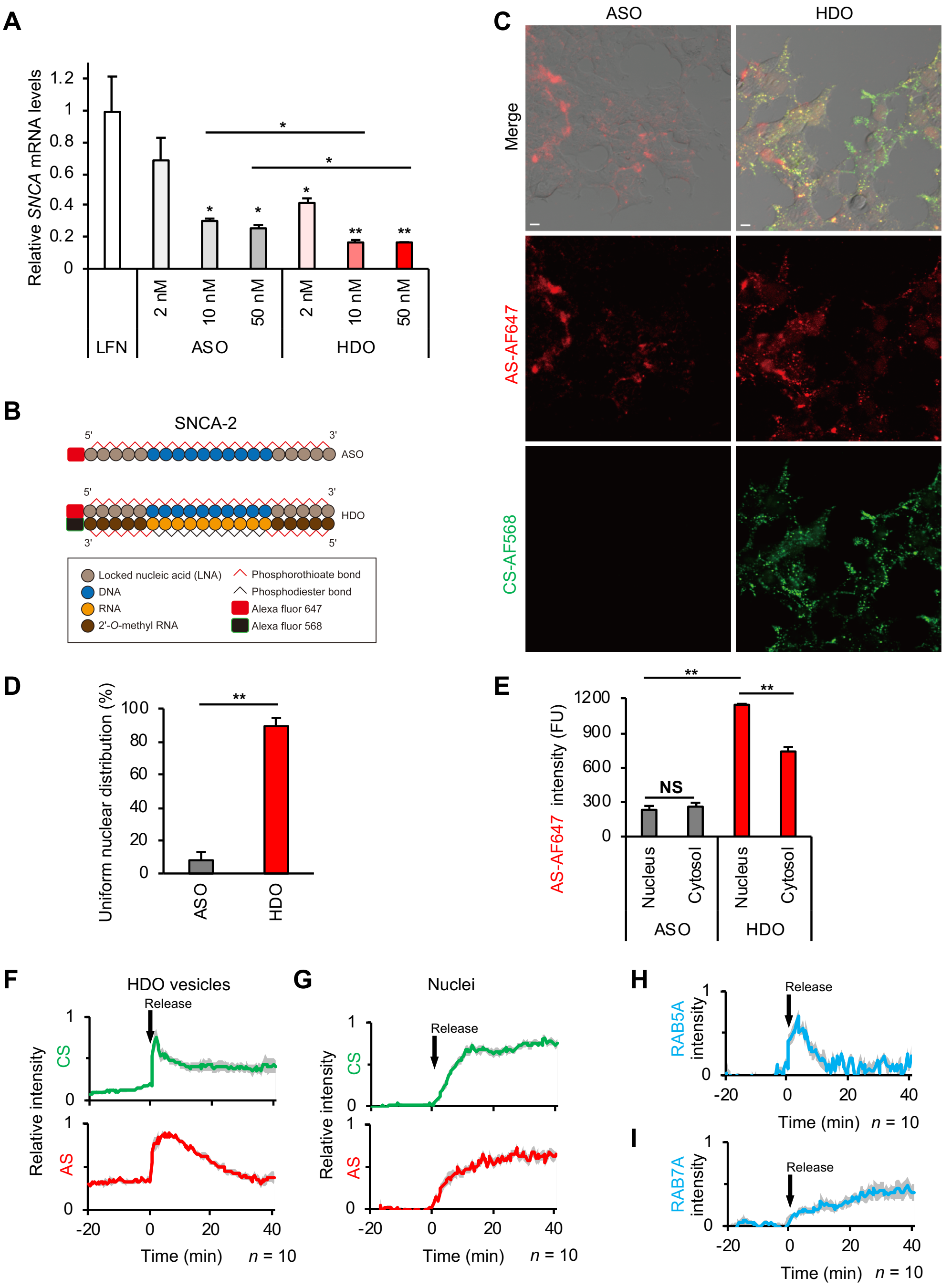

Figure S10. Intracellular Mechanism of HDO by Highly Efficient SNCA-2 Sequence
To evaluate the intracellular mechanism of HDO by a highly efficient sequence, we designed another SNCA-2 sequence targeting intron region of *SNCA* (Alpha-Synuclein). HEK 293T cells were transfected with Lipofectamine RNAiMAX. (A) RT-qPCR analysis of relative *SNCA* mRNA levels 24 h after transfection, normalized to *GAPDH* (* $p < 0.05$, ** $p < 0.01$ vs. lipofectamine (LFN) control; n = 3; mean ± SEM). 50% inhibitory concentration (IC_{50}) of ASO and HDO of SNCA-2 sequence were calculated as 3.7 nM and 2.2 nM, respectively. (B) Designs of dye-conjugated ASO and HDO, targeting intron region of *SNCA* , where complementary strand (CS) was labeled with AF568, and antisense strand (AS) was labeled with AF647. (C) Representative images 24 h after transfection with 50 nM ASO and HDO. The upper row shows merged images of differential interference contrast (DIC), AS-AF647 (red) and CS-AF568 (green). AF647 signals were excited by a 646 nm laser and detected through a 700 (663-738) nm filter. AF568 signals were excited by a 560 nm laser and detected through a 595 (570-620) nm filter. Bar = 10 µm. (D) Percentage of cells with uniform nuclear distribution 24 h after transfection with 50 nM ASO or HDO (** $p < 0.01$; n = 3 images for every 50 cells). (E) Mean intensities of AS-AF647 in the nucleus or cytosol 24 h after transfection with 50 nM ASO or HDO, presented as absolute values normalized to the background signal. (** $p < 0.01$; NS, not significant; n = 3 images for every 50 cells). (F, G) Time-lapse images were taken every 30 seconds after transfection with 50 nM HDO, and sequential changes of AS-AF647 and CS-AF568 signals in HDO-releasing vesicles (F) and nuclei (G) were measured. Separation-related nuclear distribution of both strands just after cytosolic release was observed. (H, I) Cells expressing GFP-labeled RAB5A (H) or RAB7A (I) were transfected with 50 nM HDO, and imaged every 30 seconds. Sequential changes of RAB5A (H) or RAB7A (I) signals in the HDO-releasing vesicles were measured. GFP signals (cyan)were excited by a 488 nm laser and detected through a 525 (500-550) nm filter. AF647 signals (red) were excited by a 646 nm laser and detected through a 700 (663-738) nm filter. AF568 signals (green) were excited by a 560 nm laser and detected through a 595 (570-620) nm filter. $t = 0$ is set just before the releases started. Mean intensities of each region were presented as relative values (0-1), with 0 being the background intensity, and 1 being the highest intensity value for each object (n = 10; ± SEM, shaded areas).

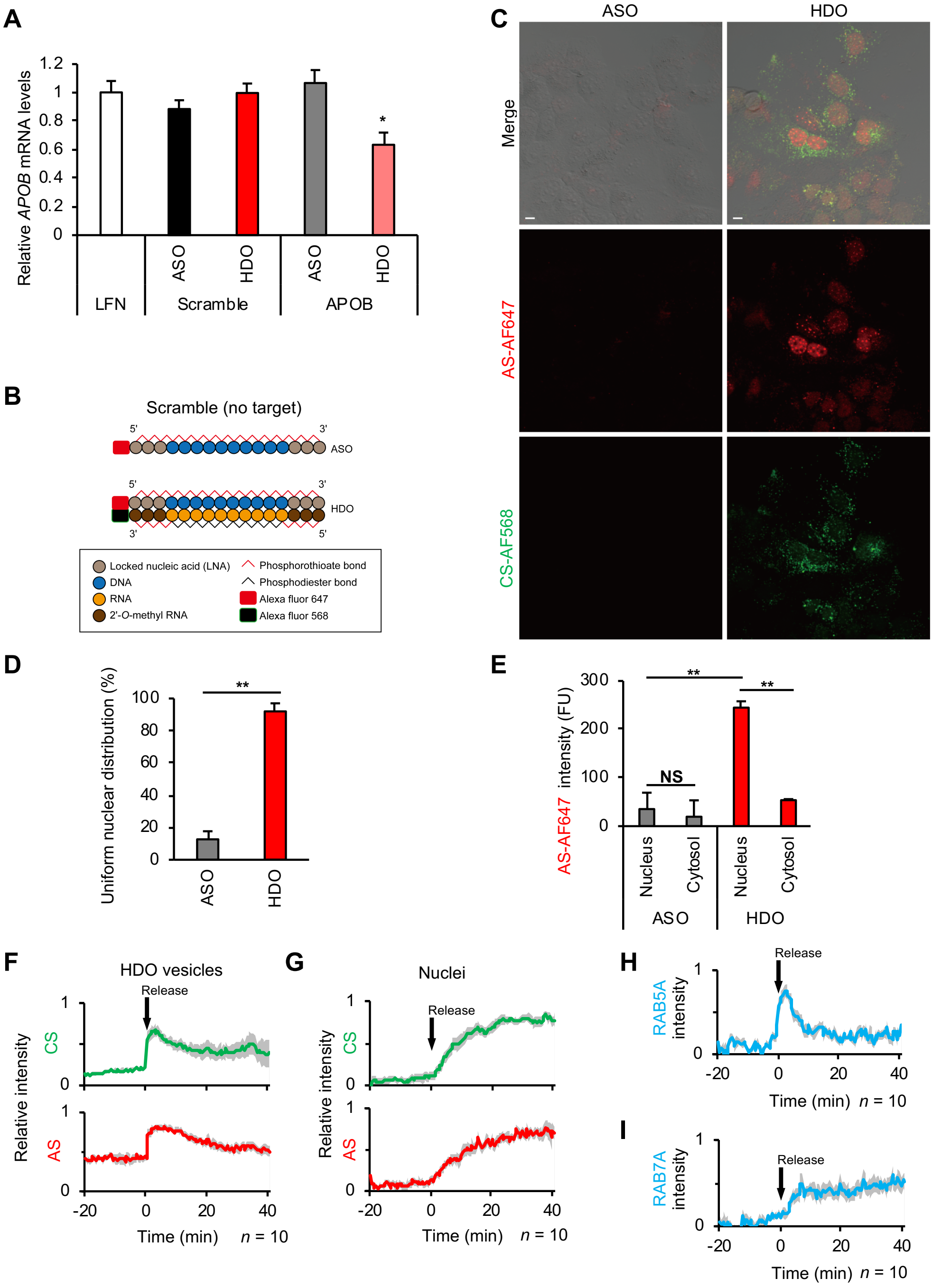

Figure S11. Intracellular Mechanism of HDO by a Scramble Sequence without any Target Gene

To confirm that our observation about intracellular mechanism of HDO are applicable indifferently to efficiency of certain sequence, we designed a scramble sequence without any target gene. Huh7 cells were transfected with Lipofectamine RNAiMAX. (A) RT-qPCR analysis of relative *APOB* mRNA levels 24 h after transfection, normalized to *GAPDH* (* $p < 0.05$ vs. lipofectamine (LFN) control; n = 3; mean ± SEM). (B) Designs of dye-conjugated ASO and HDO without any target gene, where complementary strand (CS) was labeled with AF568, and antisense strand (AS) was labeled with AF647. (C) Representative images 24 h after transfection with 50 nM ASO and HDO. The upper row shows merged images of differential interference contrast (DIC), AS-AF647 (red) and CS-AF568 (green). AF647 signals were excited by a 646 nm laser and detected through a 700 (663-738) nm filter. AF568 signals were excited by a 560 nm laser and detected through a 595 (570-620) nm filter. Bar = 10 µm. (D) Percentage of cells with uniform nuclear distribution 24 h after transfection with 50 nM ASO or HDO (** $p < 0.01$; n = 3 images for every 50 cells). (E) Mean intensities of AS-AF647 in the nucleus or cytosol 24 h after transfection with 50 nM ASO or HDO, presented as absolute values normalized to the background signal. (** $p < 0.01$; NS, not significant; n = 3 images for every 50 cells). (F, G) Time-lapse images were taken every 30 seconds after transfection with 50 nM HDO, and sequential changes of AS-AF647 and CS-AF568 signals in HDO-releasing vesicles (F) and nuclei (G) were measured. Separation-related nuclear distribution of both strands just after cytosolic release was observed. (H, I) Cells expressing GFP-labeled RAB5A (H) or RAB7A (I) were transfected with 50 nM HDO, and imaged every 30 seconds. Sequential changes of RAB5A (H) or RAB7A (I) signals in the HDO-releasing vesicles were measured. GFP signals (cyan)were excited by a 488 nm laser and detected through a 525 (500-550) nm filter. AF647 signals (red) were excited by a 646 nm laser and detected through a 700 (663-738) nm filter. AF568 signals (green) were excited by a 560 nm laser and detected through a 595 (570-620) nm filter. $t = 0$ is set just before the releases started. Mean intensities of each region were presented as relative values (0-1), with 0 being the background intensity, and 1 being the highest intensity value for each object (n = 10; ± SEM, shaded areas).

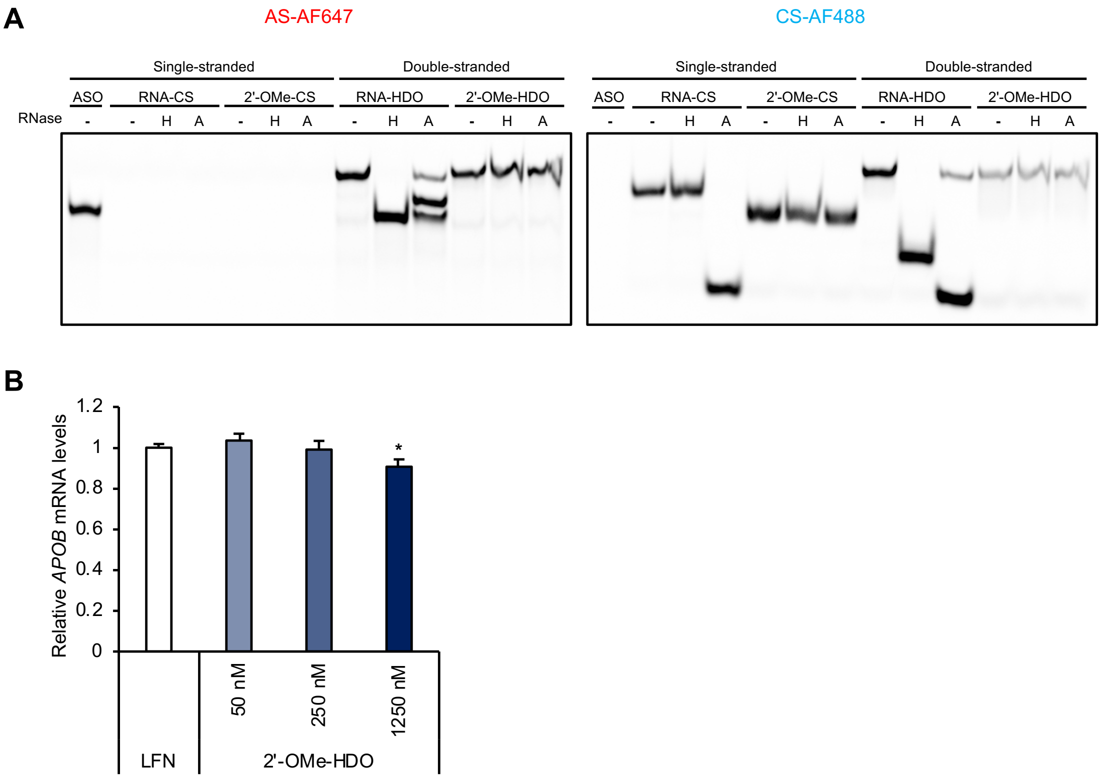

Figure S12. Cleavage-Independent Separation of 2'-OMe-HDO

(A) To evaluate the resistance of 2‘OMe-RNA for RNases, oligonucleotides targeting intron region of *APOB* mRNA, where antisense strand (AS) and complementary strand (CS) were labeled with AF647 and with AF488, respectively, were treated with RNase A or H. After electrophoresis in 20% polyacrylamide gel, fluorescence of each dye was imaged. The result showed that 2'-OMe-HDO was resistant to RNase H and A. (B) To see if cleavage-independent separation of 2'-OMe-HDO could induce antisense activity, dose-escalation study was performed. RT-qPCR analysis 24 h after transfection with 1250 nM 2'-OMe-HDO targeting intron region of *APOB* showed slight significant nuclear activity (normalized to *GAPDH*; * $p < 0.05$ vs lipofectamine (LFN) control; n = 3; mean ± SEM).

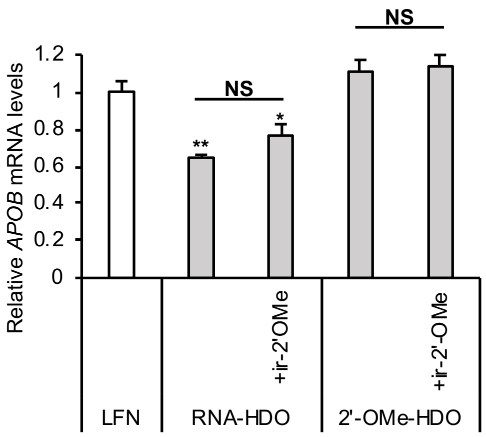

Figure S13. Co-Transfection with Irrelevant 2'-OMe Strand

To exclude a possibility that non-cleavable 2'-OMe strand competed with antisense strand (AS) or inhibited its activity in a sequence-independent manner, 50 nM RNA-HDO or 2'-OMe-HDO targeting intron *APOB* was co-transfected with 50 nM irrelevant full 2'-OMe strands targeting mouse *Malat* (ir-2'-OMe). RT-qPCR analysis 24 h after transfection showed no significant change between single transfection and co-transfection groups (normalized to *GAPDH*; * $p < 0.05$, ** $p < 0.01$ vs lipofectamine (LFN) control; NS, not significant; n = 3; mean ± SEM).

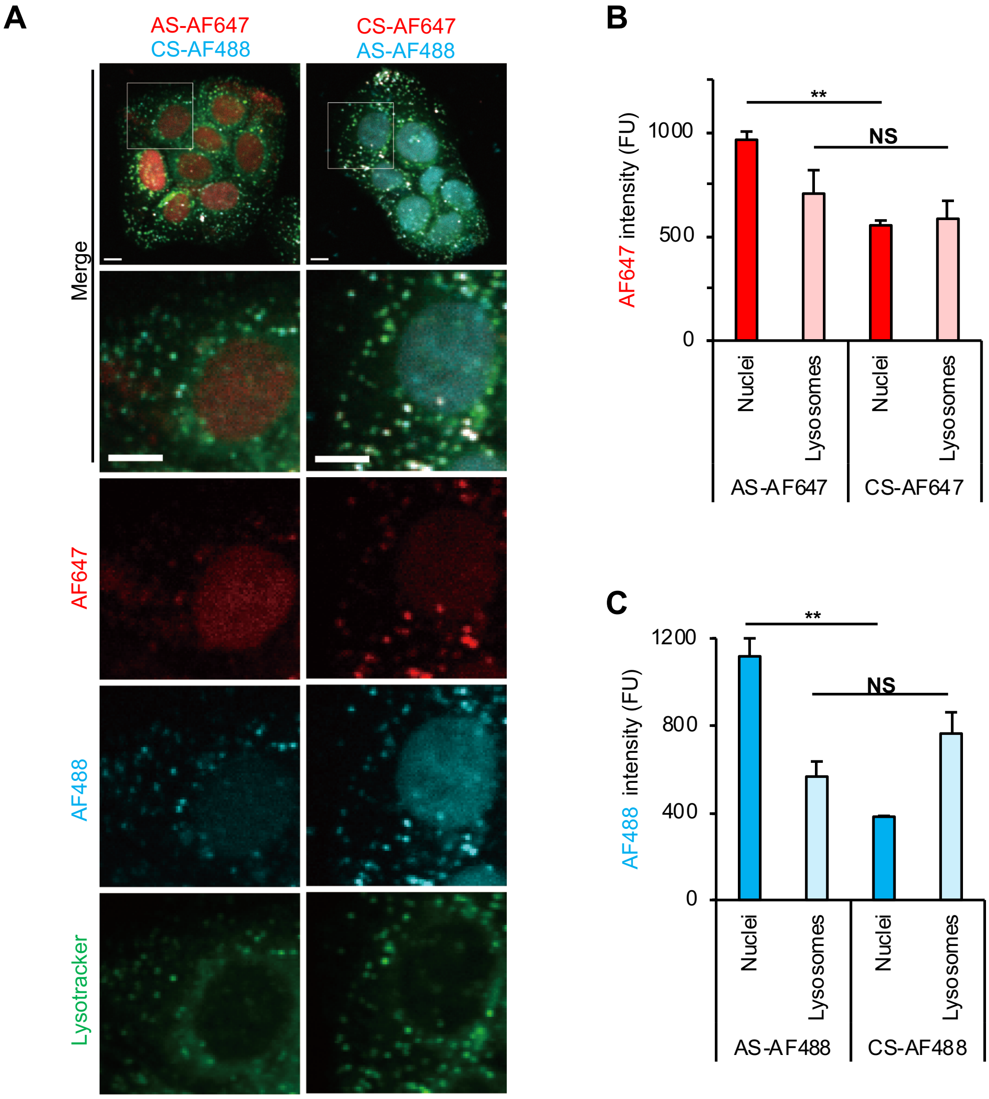

Figure S14. Accumulation of 2'-OMe-RNA Complementary Strand in Nuclei and Lysosomes
To evaluate localization of non-cleavable 2'-OMe-RNA complementary strand (CS), we imaged and evaluated its co-localization with nuclei or lysosomes. (A) Representative images 24 h after transfection with 50 nM 2'-OMe -HDO targeting intron *APOB*, composed of antisense strand (AS)-AF647 and CS-AF488 (left), or CS-AF647 and AS-AF488 (right). Lysosomes were labeled with lysotracker-RFP (green) , which was excited by a 560 nm laser and detected through a 595 (570-620) nm filter. AF647 signals (red) were excited by a 646 nm laser and detected through a 700 (663-738) nm filter. AF488 signals (cyan) were excited by a 488 nm laser and detected through a 525 (500-550) nm filter. Bar = 10 µm. (B, C) Mean signal intensities of AF647 (B), or AF488 (C) in nuclei and in lysosomes. Measurements from the experiment (A) presented as absolute values normalized to background levels. (** $p < 0.01$; NS, not significant; n = 3 images for every 50 cells, or 150 lysosomes; mean ± SEM).

Movie Legends

Movie 1. Separation, Cytosolic Release, and Rapid Nuclear Transport of HDO

Live cell time-lapse images of the HDO-releasing vesicle presented in Figure 2D, E. Images were taken, every 30 sec, just after transfection with 50 nM HDO targeting intron *APOB*, composed of antisense strand-AF647 (red) and complementary strand-AF568 (green). AF647 signals were excited by a 646 nm laser and detected through a 700 (663-738) nm filter. AF568 signals were excited by a 560 nm laser and detected through a 595 (570-620) nm filter. Bar = 10 µm.

Movie 2. Inflow Signals from HDO-Releasing Vesicles

(A, B) Live cell time-lapse images of cells which showed inflow signals from HDO-releasing vesicles, Images were taken in a high sensitivity setting compared to experiments in Figure 2, every 30 sec, just after transfection with 50 nM HDO targeting intron *APOB*, composed of antisense strand-AF647 (red) and complementary strand-AF568 (green). AF647 signals were excited by a 646 nm laser and detected through a 700 (663-738) nm filter. AF568 signals were excited by a 560 nm laser and detected through a 595 (570-620) nm filter. Bar = 10 µm.

Movie 3. Co-Localization of HDO Vesicles Foci with Early Endosomes

Live cell time-lapse images of the HDO releasing vesicle presented in Figures 3A and 3B. Cells expressing GFP-labeled *RAB5A* (cyan) were transfected with 50 nM HDO targeting intron *APOB* (antisense strand-AF647 (red), complementary strand-AF568 (green)). GFP signals were excited by a 488 nm laser and detected through a 525 (500-550) nm filter. AF647 signals were excited by a 646 nm laser and detected through a 700 (663-738) nm filter. AF568 signals were excited by a 560 nm laser and detected through a 595 (570-620) nm filter. Images were taken just after transfection, every 1 min. Bar = 10 µm.

Movie 4. Co-Localization of HDO Vesicles Foci with Late Endosomes

Live cell time-lapse images of the HDO releasing vesicle presented in Figure 3D and 3E. Cells expressing GFP-labeled *RAB7A* (cyan) were transfected with 50 nM HDO targeting intron *APOB* (antisense strand-AF647 (red), complementary strand-AF568 (green)). GFP signals were excited by a 488 nm laser and detected through a 525 (500-550) nm filter. AF647 signals were excited by a 646 nm laser and detected through a 700 (663-738) nm filter. AF568 signals were excited by a 560 nm laser and detected through a 595 (570-620) nm filter. Images were taken just after transfection, every 2 min. Bar = 10 µm.
