# Supplementary material for: Separation-related rapid nuclear transport of DNA/RNA heteroduplex oligonucleotide: unveiling distinctive intracellular trafficking
Source: Mol Ther Nucleic Acids. 2020 Dec 3;23:1360–70. doi: 10.1016/j.omtn.2020.11.022 (PMC7933600; doi:10.1016/j.omtn.2020.11.022)
Supplement: Document S2. Article plus supplemental information [file mmc7.pdf]

Molecular Therapy
Nucleic Acids
Original Article

Separation-related rapid nuclear transport of DNA/RNA heteroduplex oligonucleotide: unveiling distinctive intracellular trafficking

Daisuke Ono,[1] Ken Asada,[1] Daishi Yui,[1] Fumika Sakaue,[1] Kotaro Yoshioka,[1] Tetsuya Nagata,[1] and Takanori Yokota[1]

[1]Department of Neurology and Neurological Science, Graduate School of Medical and Dental Sciences and Center for Brain Integration Research, Tokyo Medical and Dental University, 1-5-45 Yushima, Bunkyo-Ku, Tokyo 113-8519, Japan

DNA/RNA heteroduplex oligonucleotide (HDO), composed of DNA/locked nucleic acid (LNA) antisense oligonucleotide (ASO) and complementary RNA, is a next-generation antisense therapeutic agent. HDO is superior to the parental ASO in delivering to target tissues, and it exerts a more potent gene-silencing effect. In this study, we aimed to elucidate the intracellular trafficking mechanism of HDO-dependent gene silencing. HDO was more preferably transferred to the nucleus after transfection compared to the parental ASO. To determine when and where HDO is separated into the antisense strand (AS) and complementary strand (CS), we performed live-cell time-lapse imaging and fluorescence resonance energy transfer (FRET) assays. These assays demonstrated that HDO had a different intracellular trafficking mechanism than ASO. After endocytosis, HDO was separated in the early endosomes, and both AS and CS were released into the cytosol. AS was more efficiently transported to the nucleus than CS. Separation, endosomal release, and initiation of nuclear transport were a series of time-locked events occurring at a median of 30 s. CS cleavage was associated with efficient nuclear distribution and gene silencing in the nucleus. Understanding the unique intracellular silencing mechanisms of HDO will help us design more efficient drugs and might also provide insight into innate DNA/RNA cellular biology.

INTRODUCTION

Antisense oligonucleotide (ASO) is a single-stranded therapeutic oligonucleotide that modulates RNA functions by binding to the targeted RNA through Watson-Crick base pairing.[1,2] ASO is typically designed as a "gapmer" structure, where wings of chemically modified nucleotides flank both sides of a central portion of DNA. ASO gapmer with phosphorothioate (PS) backbone is highly potent and has recently been applied to clinical settings.[3,4]

We recently developed a novel highly efficient oligonucleotide, DNA/RNA heteroduplex oligonucleotide (HDO), which is composed of an antisense gapmer (DNA nucleotides flanked by LNAs) and complementary RNA (cRNA).[5–7] HDO showed improved delivery to the target tissue by conjugating tocopherol to the cRNA strand. Additionally, Toc-HDO presented 4.8 times higher gene silencing effect than parental ASO, revealed after measuring the delivered oligonucleotide content.[5] MicroRNA-targeting HDO (HDO-antimiR) displayed high potency in cleaving the target microRNA, whereas the parental ASO exerted its potency via a steric blocking (not cleaving) mechanism.[6] Additionally, HDO-antimiR conjugated with GalNAc was more potent in the liver than the parent ASO conjugated with GalNAc, where delivery efficiency of HDO was comparable to that of ASO.[6] These findings suggested that HDO may have a distinct intracellular trafficking pathway and processing machinery different from the single-stranded ASO.

Intracellular trafficking pathways of oligonucleotides are diverse and depend on their structures, such as chemical modifications, ligand conjugations, and association with nanocarriers.[8–11] For therapeutic applications, intracellular trafficking of ASO gapmers with PS backbones (referred to as PS-ASOs) has been intensely investigated.[8,11] Cells internalize PS-ASOs by endocytosis; they are trafficked from early endosomes to late endosomes, and finally to lysosomes.[12,13] PS-ASOs have to be released from those endocytic vesicles into the cytosol and nucleus, where they bind to the target mRNA and cleave it by RNase H1.[8,11,14,15]

In our previous study, we investigated the intracellular mechanism of HDO.[5] From the imaging studies *in vivo*, we suspected that the antisense strand (AS) of HDO was more robustly distributed into the nucleus compared with the parental ASO. Therefore, we hypothesized that an effective gene silencing by HDO could be attributed not only to the efficiency of cellular uptake but also to the distinctive intracellular localization patterns. Unlike single-stranded ASO, double-stranded HDO has to be separated into AS and complementary strand (CS) in the cell in order for AS to bind to the target RNAs and exert antisense activity. For the elucidation of these intracellular trafficking mechanisms of HDO, there are technical limitations—a

Received 11 July 2020; accepted 28 November 2020;
https://doi.org/10.1016/j.omtn.2020.11.022.

Correspondence: Takanori Yokota, Department of Neurology and Neurological Science, Graduate School of Medical and Dental Sciences, Tokyo Medical and Dental University, 1-5-45 Yushima, Bunkyo-Ku, Tokyo 113-8519, Japan.
E-mail: tak-yokota.nuro@tmd.ac.jp

 © 2020 The Authors.
This is an open access article under the CC BY license (http://creativecommons.org/licenses/by/4.0/).

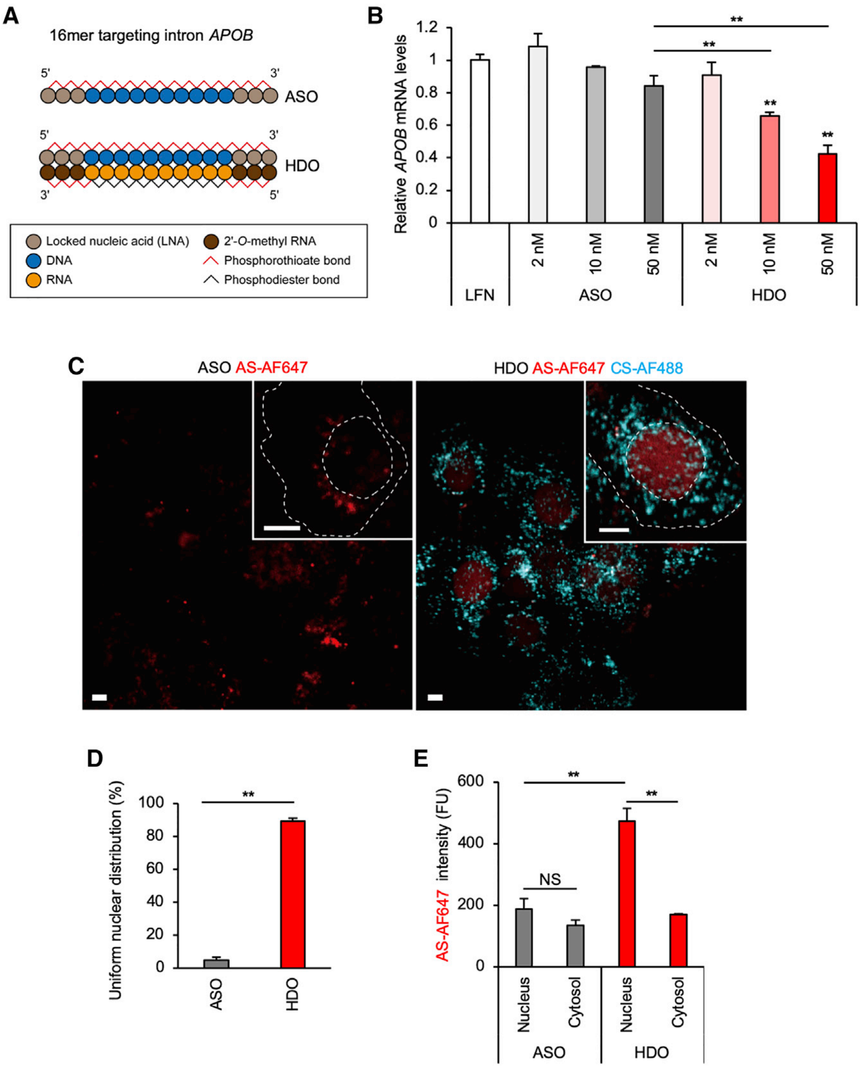

Figure 1. Efficient gene silencing of HDO in the nucleus after cytosolic delivery

(A) Design of ASO and HDO, targeting intron region of *APOB* pre-mRNA. The ASO is the 16-mer gapmer in which 10 DNA oligonucleotides are flanked by 3 LNA oligonucleotides, and all internucleotide linkages were modified by phosphorothioate (PS) substitution. In HDO, the complementary RNA strand is flanked by PS-modified 2′-*O*-methyl RNAs, which are complementary to LNA.[5] (B) Quantitative real-time PCR analysis of relative *APOB* mRNA levels was normalized to those of *GAPDH* mRNA 24 h after transfection of intron-targeting HDO or ASO (**p < 0.01 versus Lipofectamine [LFN] control; n = 3; mean ± SEM). (C) Representative images 24 h after transfection of the cells with 50 nM ASO or HDO. AF647 labels the antisense strand (AS) of HDO, and AF488 labels the complementary strand (CS), where FRET does not occur. AF647 signals (red) were excited by a 646 nm laser and detected through a 700 (663–738) nm filter. AF488 signals (cyan) were excited by a 488 nm laser and detected through a 525 (500–550) nm filter. Dotted lines represent nuclear or plasma membrane outlined by differential interference contrast (DIC) images. Bar, 10 μm. (D) Percentage of cells with uniform nuclear distribution 24 h after transfection with 50 nM ASO or HDO. (E) Mean intensities of AS-AF647 in the nucleus or cytosol 24 h after transfection with 50 nM ASO or HDO, presented as absolute values normalized to background levels. (**p < 0.01; NS, not significant; n = 3 images for each 50 cells).

conventional snapshot imaging cannot detect the time and site of HDO separation. To tackle this problem, we performed live-cell time-lapse imaging utilizing fluorescence resonance energy transfer (FRET) assay and demonstrated a unique intracellular silencing mechanism by HDO.

RESULTS

Efficient gene silencing of HDO in the nucleus after cytosolic delivery

To address our hypothesis that HDO is more efficiently transferred into the nucleus, we designed ASO and HDO molecules that targeted the intron region of *APOB* pre-mRNA (Figure 1A), which is localized in the nucleus.[15] We evaluated how a spatially specifically introduced HDO or ASO can regulate nuclear expressing target pre-mRNA in human hepatocellular carcinoma Huh-7 cells transfected using Lipofectamine RNAiMAX, which effectively delivers small interfering RNAs (siRNAs) or single-stranded nucleotides into the cytosol.[16–18] *APOB* expression was measured 24 h after transfection, using quantitative real-time PCR. Our results showed a dose-dependent nuclear silencing effect of HDO, whereas no significant effect was observed with the parent ASO (Figure 1B; Figure S1).

Next, to visualize the nuclear distribution of HDO, we labeled AS with the fluorophore AF647 (excitation/emission: 650/665 nm), and CS with AF488 (490/525 nm), where excitation wavelengths are so far that the FRET phenomenon seldom occurs. Imaging results 24 h after HDO transfection showed a completely different distribution pattern compared to ASO (Figure 1C). An intense and well-demarcated nuclear signal of AS was observed in almost all HDO-transfected cells. Additionally, a dotted signal of CS, accumulated dominantly in the cytosol, was detected. Meanwhile, the same dose of ASO presented just a diffused or partly dotted weak distribution throughout the cells.

Before the time-lapse imaging we will show in Figure 2, we defined the uniform and well-demarcated nuclear AS signals as "nuclear transport" and counted them (Figure 1D). HDO showed a high uniform nuclear distribution rate, which was concordant with its potency (Figure 1B). We then quantified the mean intensity in both the nucleus and cytosol (Figure 1E). HDO presented strong preference for the nucleus. On the other hand, ASO was distributed to the nucleus and

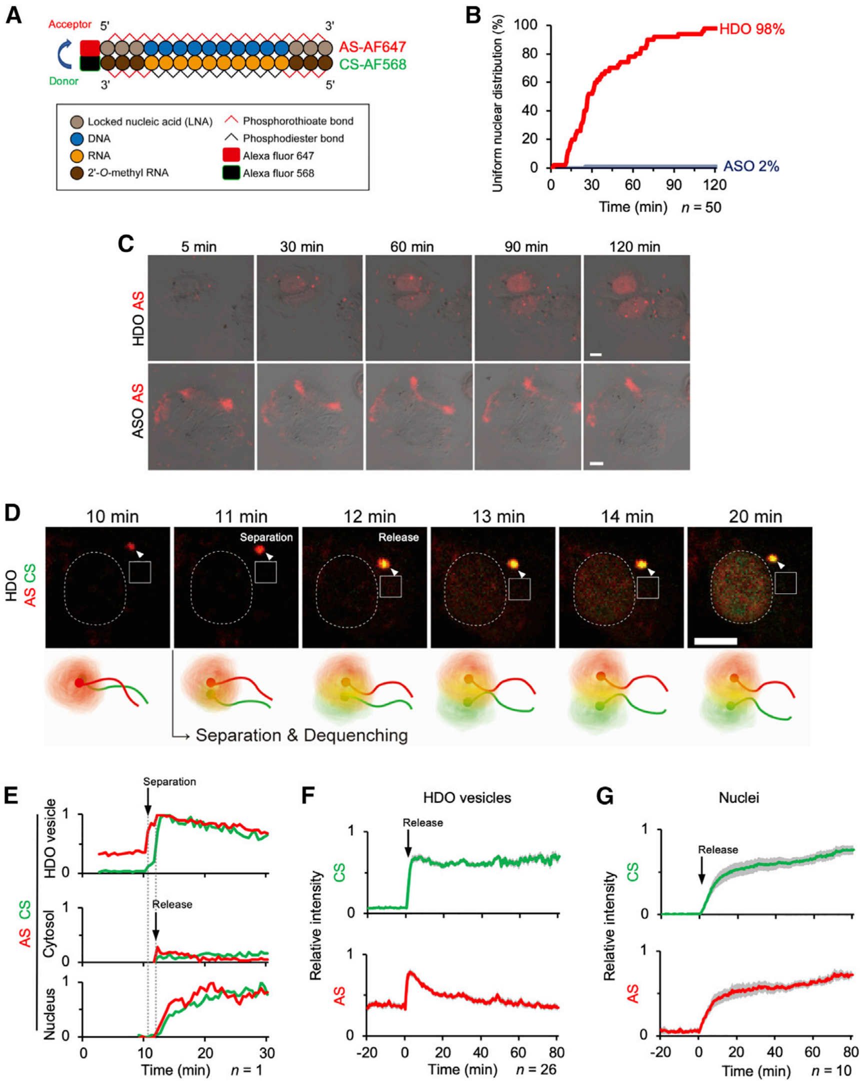

Figure 2. Separation and rapid transport of HDO into the nucleus

(A) Design of dye-conjugated HDO targeting intron *APOB* with FRET system. AF647 labels the AS of HDO, and AF568 labels the CS, where CS-AF568 signals become quenched. (B) Sequential changes in the percentage of cells with uniform nuclear distribution after 50 nM transfection of HDO or ASO. (n = 50). (C) Representative live-cell images within 2 h after transfection with 50 nM HDO or ASO, presented as merged images of DIC and AS-AF647 (red). AF647 signals were excited by a 646 nm laser and detected through a 700 (663–738) nm filter. Bar, 10 μm. (D) Live-cell time-lapse images of the HDO-releasing vesicle (arrowhead). As shown in the bottom scheme, AF568 signal (green) represents separated CS, and AF647 signal (red) corresponds to the AS signal from the wound or isolated form. Therefore, newly observed yellow signal (colocalization of red and green) means separation of HDO. Dotted circles and squares represent nuclear and cytosolic areas, respectively, which are shown in (E). Images were acquired just after transfection with 50 nM HDO, every 30 s. AF647 signals were excited by a 646 nm laser and detected through a 700 (663–738) nm filter. AF568 signals were excited by a 560 nm laser and detected through a 595 (570–620) nm filter. Bar, 10 μm. (E) Sequential changes of AS-AF647 (red) and CS-AF568 (green) signals in the nucleus, cytosol, and the HDO-releasing vesicle of the cell shown in (D). Mean intensities of each region are presented as relative values (0–1), with 0 being the background intensity and 1 being the highest intensity value for each object (except for the cytosol using the highest intensity of the nucleus). (F and G) Mean relative intensity of AS-AF647 and CS-AF568 in HDO-releasing vesicles (F) and nuclei (G). *t* = 0 is set just before the releases started. (F, n = 26; G, n = 10 G; ± SEM, shaded areas). Results were pooled from three experiments per condition in (F).

cytosol without significant preference. Thus, we supposed that the uniform and well-demarcated nuclear localization was a distinctive character of HDO, associated with nuclear potency.

Additionally, in co-transfection experiments with HDO and ASO, we confirmed that HDO was also superiorly distributed compared to ASO (Figure S2). Fluorescent dyes transfected without the oligonucleotide conjugation were not detectable in the cells (Figure S3). To evaluate delivery methods other than lipid transfection, we performed gymnotic (free uptake) delivery of ASO or HDO, but it presented no significant nuclear distribution (Figure S4).

Separation and rapid transport of HDO into the nucleus

To observe the separation of HDO, we designed an HDO composed of AF647-labeled AS and AF568-labeled CS and applied the FRET mechanism[19–23] (Figure 2A). Excitation/emission wavelengths of these dyes are 650/665 and 578/603 nm, respectively, which are close enough for FRET to occur. In the double-stranded wound form, the energy absorbed by CS-AF568 after excitation with a 560 nm laser was transferred to AS-AF647. In other words, CS-AF568 signal became quenched, and AS-AF647 produced a signal through FRET. If HDO was separated, the isolated CS-AF568 could not transfer the energy to AS-AF647, and thus the CS-AF568 signal would increase and become dequenched. We confirmed the FRET and dequenching signals in our experimental system by measuring the fluorescence intensity of dye-conjugated HDO in solution without cell or transfection reagent (Figure S5).

Live-cell time-lapse imaging was performed every 30 s for 120 min (Figure 2). Uniform nuclear distribution of AS was observed in 98% of HDO- and only 2% of ASO-transfected cells (Figure 2B). We repeated the experiments and deduced the following reproducible results. Immediately after the transfection, ASO-containing liposomes were visualized in the background, and a few of them were

endocytosed. Cloud-like weak ASO signals were observed without preference to the nucleus or cytosol, which presented no remarkable change within 2 h (Figure 2C).

HDO-containing liposomes were also visualized in the background, where the majority of CS-AF568 signal was quenched by FRET. A few minutes after the transfection, several liposomes were endocytosed into the cell and transported from the periphery toward the perinuclear region of the cytosol. Then, in one of the endocytosed HDO vesicles, a sudden appearance of the CS-AF568 signal was detected with a transient increase of the AS-AF647 signal. Almost simultaneously, both AS and CS were released into the cytosol and subsequent nuclear transport started (Figures 2D and 2E; Video S1).

We considered the transient increase of the AS-AF647 signal in the vesicle at the moment of the release as cancellation of self-quenching. Densely packed fluorophores, such as in liposomes, quench themselves, and this self-quenching is cancelled by the release from the vesicles.[24,25] We subsequently defined the HDO release as a sudden increase of the AS signal in the cytosol and identified an HDO-releasing vesicle by the transient increase of the AF647 signal. We traced and measured subsequent changes of signal intensities in the HDO-releasing vesicles and nuclei, where $t = 0$ was reset just before the release, and the mean relative intensity was calculated with reference to the previous study (Figures 2F and 2G).[24] The increase of the AS-AF647 signal was transient and corresponded to the cancellation of the self-quenching. The sudden and sharp increase of CS-AF568 signal just after the HDO release was reproducibly observed. Then, we confirmed that this newly generated CS-AF568 signal meant dequenching of the FRET signal, as a result of the HDO separation (Figure S6).

Cytosolic signal from the AS was weak and transient, which was swallowed up into the nucleus soon after and depleted within a few minutes (Figures 2D and 2E; Video S1). The nuclear distribution of both strands was almost homogeneous, which corresponded to the uniform nuclear distribution observed 24 h after the transfection, as mentioned above (Figures 1C and 1D). Further release from other vesicles followed, whereas the second release from the same vesicle was seldom observed. If we increased the sensitivity of fluorescence detection settings, the inflow signal from the releasing vesicles was observed (Videos S2 and S3; Figure S7). These sequential events—the separation, release, and nuclear transport of both strands—always occurred in that order. The separation occurred at a median of 27 min, and the median interval of separation to initiation of nuclear distribution was as short as 30 s (n = 50). The same phenomena were observed with 10 nM HDO, which was a comparable dose to 50 nM ASO (Figure S8). We also obtained similar results with other sequences of HDO, targeting the intron *SNCA* mRNA (Figures S9 and S10A–S10G) and a sequence without any target gene (Figures S11A–S11G).

Release of HDO from early endosomes

Cells expressing GFP-labeled endosome markers were transfected with HDO, and subsequent live-cell imaging was performed to identify the nature of the HDO-releasing vesicle. At the time of the separation, strong but transient co-localization of the releasing vesicle with RAB5A, the early endosome marker, was observed (Figures 3A–3C; Video S4). In contrast, RAB7A, a late endosome marker, was gradually co-localized with the releasing vesicle several minutes after the separation (Figures 3D–3F; Video S5). These observations were reproducibly confirmed by two additional sequences and the other cell line (Figures S10H, S10I, S11H, and S11I). Therefore, we considered that HDO was separated in and released from early endosomes.

Accumulation of AS in the nucleus and CS in lysosomes

To evaluate HDO trafficking after the endosomal release, we labeled lysosomes with RFP-lysotracker. HDO composed of AS-AF647/CS-AF488 or reverse pair AS-AF488/CS-AF647 was imaged at 24 h (Figure 4A). Signal intensities in nuclei or lysosomes were measured and presented as the mean of absolute values so that we could compare AS and CS by the same fluorescent dye (Figures 4B and 4C). Both dyes presented the same tendency that more AS accumulated in nuclei than in lysosomes and more CS accumulated in lysosomes than in nuclei.

CS cleavage and efficient gene silencing in the nucleus

To evaluate the significance of CS cleavage for gene silencing in the nucleus, we applied the RNase-resistant 2′-*O*-methyl sugar modification (2′-OMe). We confirmed that CS composed of full 2′-OMe was not cleaved *in vitro* (Figure S12A), which is in accordance with a previous *in vivo* study.[5] In the present study, HDO with a modified full 2′-OMe CS (2′-OMe-HDO) showed a much lower gene silencing effect in the nucleus than the default HDO (RNA-HDO) (Figure 5A; Figures S12B and S13). To discriminate whether HDO was wound or separated, we generated mono-labeled-HDO (CS-AF568) without FRET, as well as dual-labeled HDO (AS-AF647/CS-AF568) with FRET (Figure 5B). Mono-labeled AF568 signals reflected the amount of CS in both wound and separated forms, whereas in dual-labeled HDO, AF568 signal was quenched in the wound form but observed in the separated form.

Imaging and subsequent measurements of nuclear intensity were performed (Figures 5C–5E). 2′-OMe-HDO presented a less intense distribution of AS into the nucleus 24 h after transfection (Figure 5D) than RNA-HDO. Nuclear distribution of CS from RNA-HDO was relatively less than that of AS (Figure 4), and most of the CS existed in the separated form, because nuclear intensities of CS from RNA-HDO showed no difference between mono- and dual-labeled groups (Figure 5E). On the contrary, the difference in the CS from 2′-OMe-HDO signals between mono- and dual-labeled groups was significantly large (Figure 5E). Therefore, we considered that most of the AS from 2′-OMe-HDO in the nucleus was in a wound form and then exerted much less potency there (Figure 5A; Figure S12B). At 24 h post-transfection, more AS from 2′-OMe-HDO accumulated in the nucleus than 2′-OM-CS and both strands did in lysosomes (Figure S14). This observation suggested a distinctive nature of HDO, in which AS was more efficiently transported to the nucleus than CS, even if CS was not cleaved.

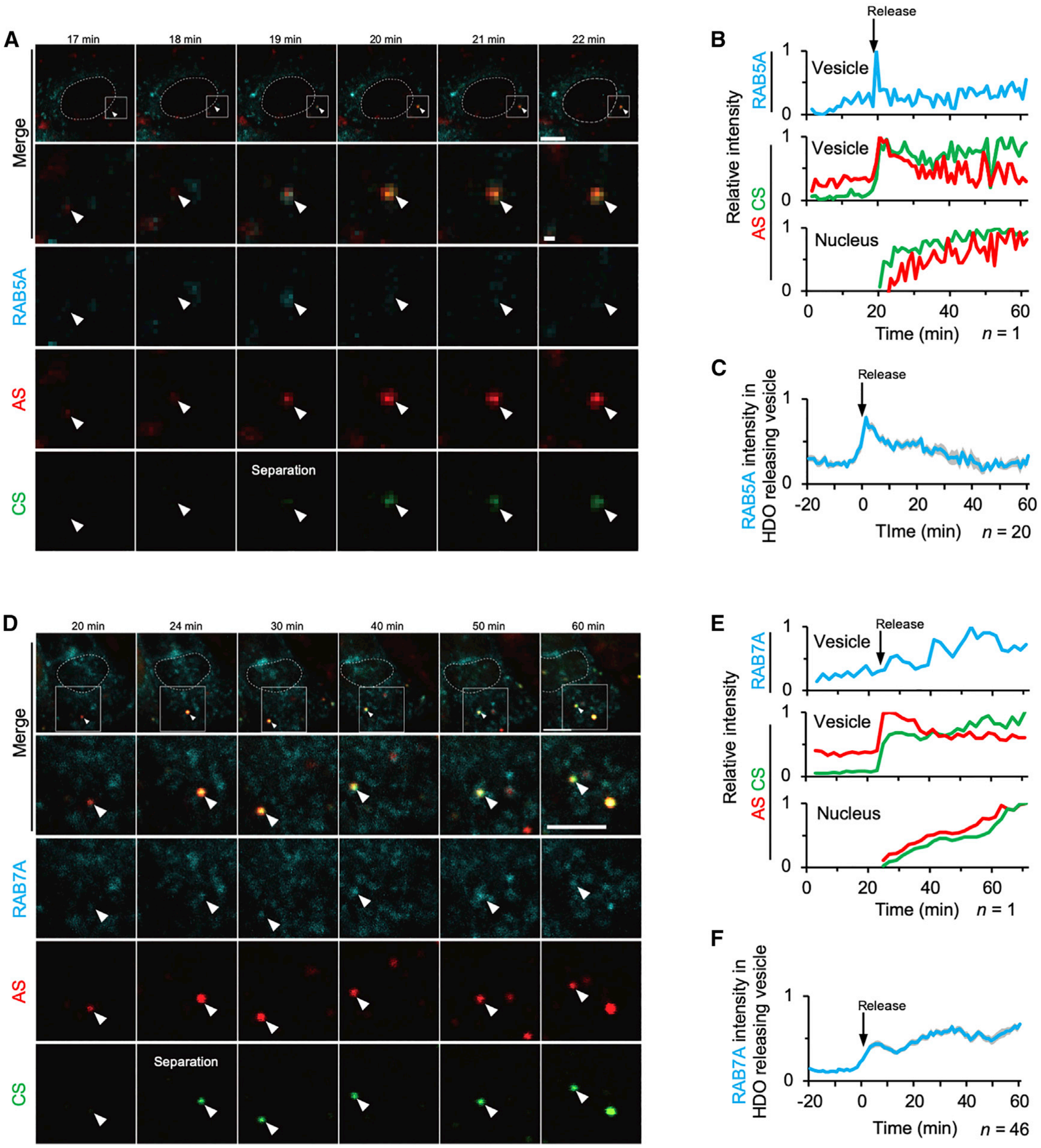

Figure 3. Release of HDO from early endosomes

(A and D) Live-cell time-lapse images of the HDO-releasing vesicle (arrowheads). Cells expressing GFP-labeled RAB5A (A–C) or RAB7A (D–F) (cyan) were transfected with 50 nM HDO targeting intron *APOB* (AS-AF647, CS-AF568). Dotted circles represent nuclei outlined by DIC images. Images were taken just after transfection, every 1 min (A–C and F) or 2 min (D and E). GFP signals were excited by a 488 nm laser and detected through a 525 (500–550) nm filter. AF647 signals (red) were excited by a 646 nm laser and detected through a 700 (663–738) nm filter. AF568 signals (green) were excited by a 560 nm laser and detected through a 595 (570–620) nm filter. Bar, 10 μm, except the second row of (A), which is 1 μm. (B and E) Sequential changes of AF647 (red), AF568 (green), and RAB5A (B) or RAB7A (E) (cyan) signals in the HDO-releasing vesicles and the nucleus of the cell shown in (A) or (D), respectively. Mean intensities of each region were presented as relative values. (C and F) Mean relative intensity of RAB5A (C) or RAB7A (F) in HDO-releasing vesicles. $t = 0$ is set just before the release started. (C, n = 20; F, n = 46; ± SEM shown as shaded areas). Results were pooled from three experiments per condition.

Decreased separation and nuclear transport in cleavage-resistant 2′-OMe-HDO

Time-lapse imaging of RNA-HDO and 2′-OMe-HDO was performed (Figure 6) to quantify the effects of CS cleavage in the early stage of HDO intracellular trafficking. Similar to RNA-HDO (Figures 6A and 6D), HDO separation just after the cytosolic release and subsequent nuclear distribution were also observed in 2′-OMe-HDO (Figures 6B and 6E). However, comparison of their peak dequenching

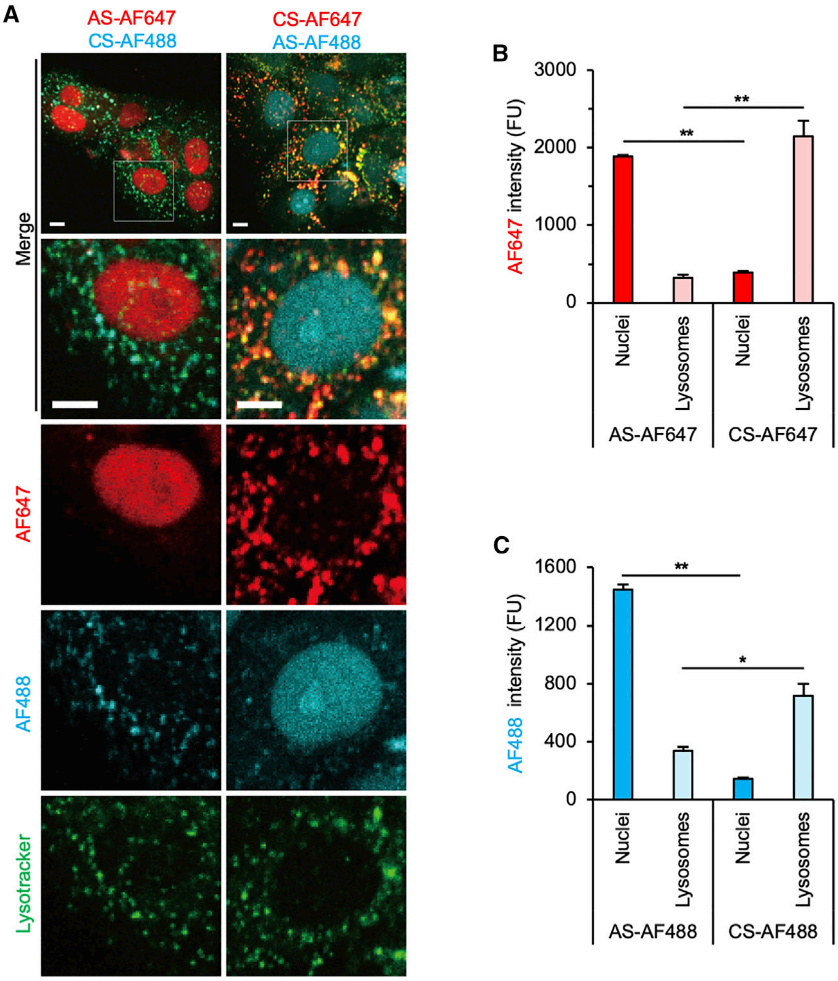

Figure 4. Accumulation of AS in the nucleus and CS in lysosomes

(A) Representative images 24 h after transfection with 50 nM HDO targeting intron *APOB*, composed of AS-AF647 and CS-AF488 (left), or CS-AF647 and AS-AF488 (right). Lysosomes were labeled with lysotracker-RFP (green), which were excited by a 560 nm laser and detected through a 595 (570–620) nm filter. AF647 signals (red) were excited by a 646 nm laser and detected through a 700 (663–738) nm filter. AF488 signals (cyan) were excited by a 488 nm laser and detected through a 525 (500–550) nm filter. Bar, 10 μm. (B and C) Mean signal intensities of AF647 (B) or AF488 (C) in nuclei and lysosomes. Measurements from the experiment (A) presented as absolute values normalized to background levels. (*p < 0.05, **p < 0.01; n = 3 images for each 50 cells, or 150 lysosomes; mean ± SEM).

levels showed that cleavage-resistant 2′-OMe-HDO presented a lesser CS dequenching than RNA-HDO (Figure 6C). 2′-OMe-HDO also showed a lesser nuclear AS signal than RNA-HDO 90 min after the release (Figure 6F). We therefore derived that cleavage-independent separation and subsequent nuclear distribution existed with 50 nM 2′-OMe-HDO but was not sufficient for nuclear antisense activity.

DISCUSSION

Distinctive intracellular trafficking of HDO

Here, we found a distinctive intracellular trafficking and processing mechanism of HDO, which is different from those of ASO or siRNA especially in two viewpoints: (1) separation-related rapid nuclear transport, and (2) cytosolic release from early endosomes. The results were reproduced using three other sequences and two cell lines. We also demonstrated the difference of intracellular trafficking between HDO and ASO by effective HDO versus non- or less-effective ASO (Figures 1–3; Figure S9), both highly effective HDO versus ASO (Figure S10), and non-target sequence (Figure S11). Furthermore, the equivalently transfected oligos presented the different effects and distributional patterns between HDO and AS (Figures S1, S2, and S8). Therefore, we concluded that HDO and ASO showed different intracellular behaviors, and these were not attributed to ineffective transfection or differences in the transfection efficiencies.

Separation-related rapid nuclear distribution

In this study, separation, release into the cytosol, and initiation of nuclear transport always occurred in this order and within a short period (median, 30 s). These strong relationships suggested that separation of HDO triggered its endosomal release and subsequent nuclear transport. Rapid release from endocytic vesicles has been previously visualized with other oligonucleotides.[21,24,26] Lipid-formulated siRNA released from late endosomes into the cytosol has been demonstrated; however, the release is not triggered by the separation of double-strand RNA, and its nuclear distribution is either unobservable or transient.[21,24] Therefore, separation-related rapid nuclear transport is the distinctive character of HDO. We additionally observed inflow signals from the HDO releasing endosomes in this study (Videos S2 and S3; Figure S7). A similar phenomenon has not been documented. Although the biological implication is unclear, this phenomenon is indicative of an unveiled intracellular structure, perhaps a "molecular tunnel" for DNA/RNA heteroduplex.

Cytosolic release from early endosomes

Independent of the delivery method (free uptake, ligand conjugation, or lipid formulation), oligonucleotides are mostly internalized by endocytosis and then trafficked through endosomal compartments,[9,10] where their cytosolic release is an essential step to exert their gene silencing activity in the cytosol or nucleus.

The current study showed HDO was released from the vesicles which transiently expressed the early endosomal marker at the moment of release. Thereafter, the vesicles gradually expressed the late

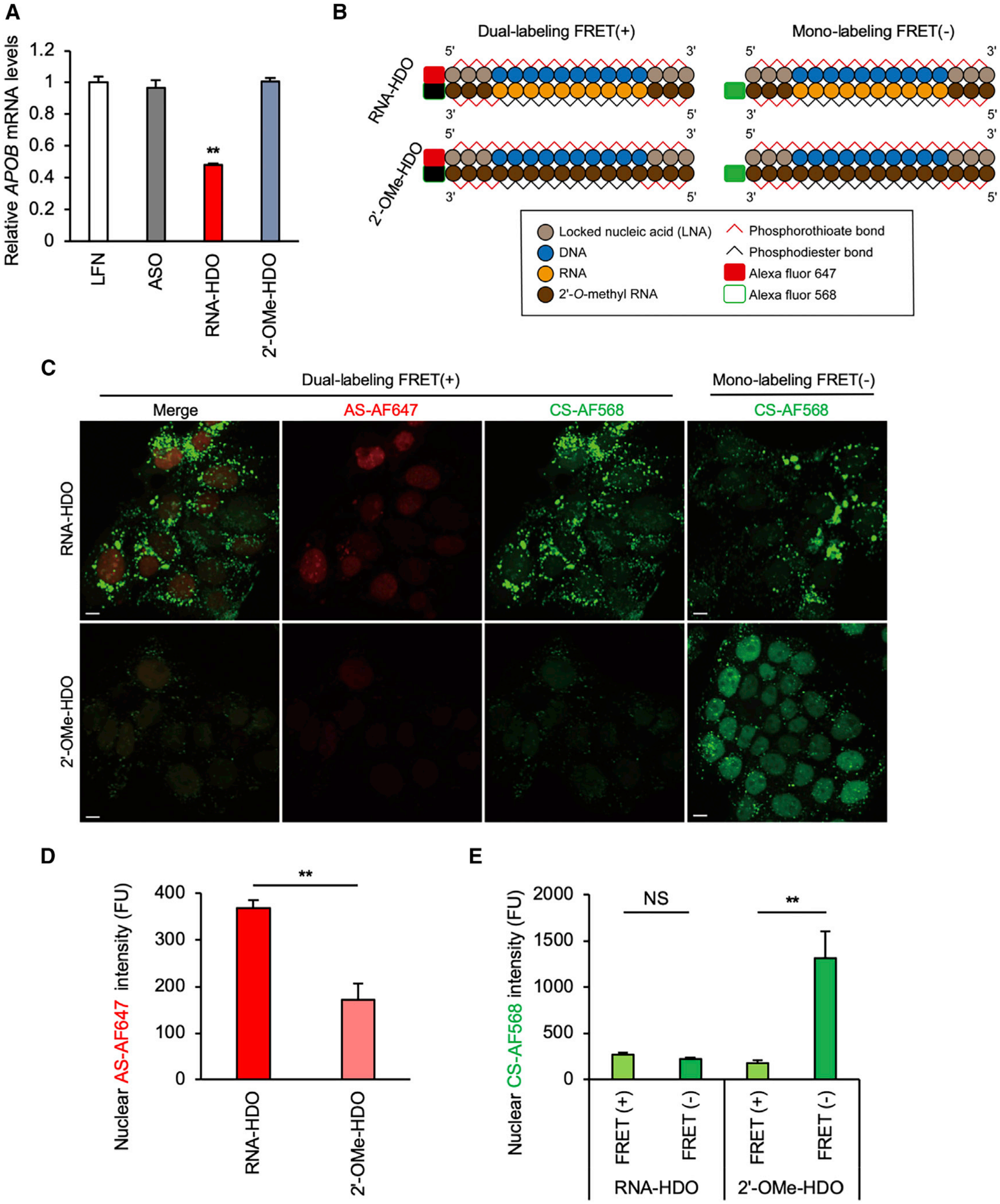

Figure 5. Cleavage of CS and efficient gene silencing in the nucleus

To evaluate the effect of CS degradation, we used RNase-resistant full 2′-OMe-strand as CS. (A) Quantitative real-time PCR analysis of relative *APOB* mRNA levels normalized to *GAPDH* mRNA 24 h after transfection with 50 nM ASO, RNA-HDO, or 2′-OMe-HDO targeting intron *APOB* (**p < 0.01 versus LFN control; n = 4; mean ± SEM). (B) Designs of dye-conjugated HDOs, where RNA or full 2′-OMe was labeled with AF568, and AS was labeled with AF647 (dual-labeling with FRET) or not labeled (mono-labeling without FRET). (C) Images 24 h after transfection with 50 nM HDOs. AF647 signals (red) were excited by a 646 nm laser and detected through a 700 (663–738) nm filter. AF568 signals (green) were excited by a 560 nm laser and detected through a 595 (570–620) nm filter. Images were obtained by weaker sensitivity settings than those of Figure 2 to maximize a slight difference of nuclear signals between RNA-HDO and 2′-OMe-HDO. Bar, 10 μm. (D and E) Mean nuclear intensities of AS-AF647 (D) or CS-AF568 (E) presented as absolute values normalized to background levels (**p < 0.01; NS, not significant; n = 3 images for each 50 cells; mean ± SEM).

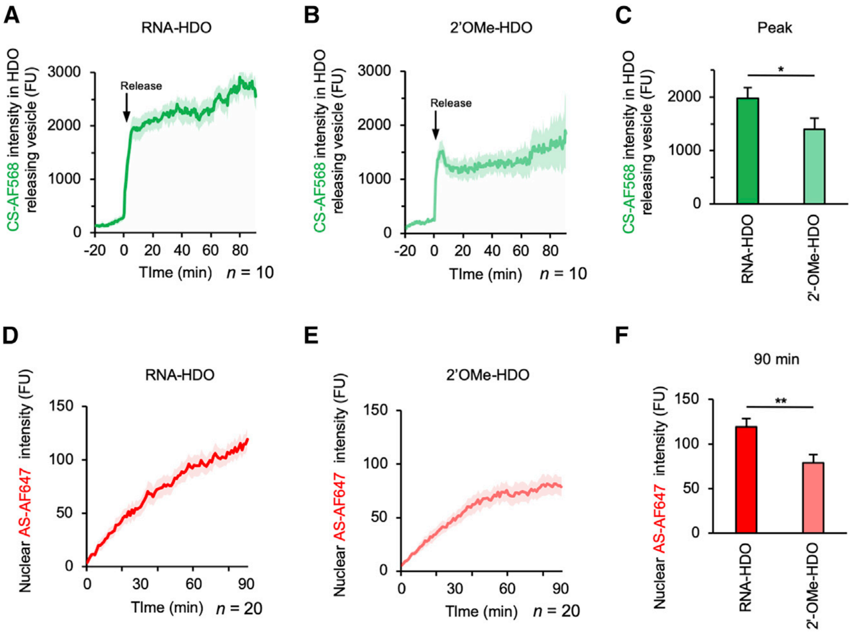

Figure 6. Decreased separation and nuclear transport in cleavage-resistant 2′-OMe-HDO

Time-lapse imaging was performed after transfection with 50 nM RNA-HDO or 2′-OMe-HDO targeting intron *APOB*, where RNA or full 2′-OMe was labeled with AF568, and AS was labeled with AF647 to quantify the effects of CS cleavability in the early stage of HDO intracellular trafficking. Images were acquired just after transfection for 120 min, every 1 min. $t = 0$ was set just before the releases started. AF647 signals (red) were excited by a 646 nm laser and detected through a 700 (663–738) nm filter. AF568 signals (green) were excited by a 560 nm laser and detected through a 595 (570–620) nm filter. Images were obtained by weaker sensitivity settings than those of Figure 2 to maximize a slight difference of nuclear signals between RNA-HDO and 2′-OMe-HDO. (A and B) Sequential changes of CS-AF568 in HDO-releasing vesicles presented as absolute values normalized to background levels. (n = 10; ± SEM shown as shaded areas). (C) Mean intensities of CS-AF568 in HDO-releasing vesicles at peak levels (5.5 min after the releases) presented as absolute values normalized to background levels (*$p < 0.05$; n = 10; mean ± SEM). (D and E) Sequential changes of AS-AF647 signals in nuclei presented as absolute values normalized to background levels. (n = 20; ± SEM shown as shaded areas). (F) Mean intensities of AS-AF647 in nuclei at 90 min after the releases presented as absolute values normalized to background levels (**$p < 0.01$; n = 20; mean ± SEM).

endosomal marker. In a previous study, Annexin 2 facilitates PS-ASO trafficking from early to late endosomes, where it may also contribute to PS-ASO release.[14] Lipid-formulated siRNAs are released from the late endosome, which is functionally related to the cholesterol transport protein NPC1.[27] HDO-binding protein has not been identified, but it might exist in the early endosome and mediate cytosolic release.

Cleavage of CS and efficient gene silencing in the nucleus

Our experiments with 2′-OMe-HDO revealed that cleavage-independent separation existed (Figure 6), but it resulted in less nuclear distribution, where a larger amount of AS was in the wound form (Figure 5), and more HDO was needed for gene silencing (Figure S12B). Therefore, the cleavability of CS was associated with the efficient gene silencing in the nucleus.

In a previous study, we examined the cleavage site of CS *in vivo* using northern blotting and concluded that CS is cleaved by unidentified enzymes, most unlikely by RNase H1.[5] As we discussed above, HDO separation in early endosomes triggered the cytosolic release and subsequent nuclear transport. Once CS was cleaved, melting temperature dramatically decreased, and separation would have been accelerated. Therefore, it is reasonable to consider that CS was cleaved in early endosomes by unidentified RNases. On the contrary, we cannot exclude the possibility that cleavage-independent separation of default RNA-HDO could also occur in early endosomes. A relatively low melting temperature of RNA-HDO compared to 2′-OMe-HDO might support this idea. The identification of binding and cleaving proteins of HDO and elucidation of its cleaving mechanism are needed, which would aid in designing more effective drugs, by balancing the extracellular RNase-resistance and intracellular cleavability.

Innate DNA/RNA biology

We showed that the exogenously administered short DNA/RNA heteroduplex had a distinctive trafficking pathway from early endosomes into the nucleus via cytosol. Regarding the exogenous origin, pathogen-derived RNA/DNA heteroduplex has been well investigated, and it induces interferon-related immune response via nucleotide sensor proteins like Toll-like receptor 9 (TLR9).[28,29] On the contrary, endogenous DNA/RNA heteroduplex has been mainly described in nuclear functions such as RNA primer of Okazaki fragments,[30,31] ribonucleotide incorporation[32] during DNA replication, R-loops,[33] and G-quadruplexes[34] during transcription. Recently, several studies proposed its extra-nuclear function. DNA/RNA heteroduplex of R-loop structure in the nucleus is cleaved and exported to cytosol,[35,36] where the heteroduplex binds to Ago2 and regulates miRNA,[35] or single-stranded DNA functions as natural antisense after RNA cleavage by RNase H and TREX1.[36] Formulation of DNA and RNA into extracellular vesicles was also reported in cancer cells.[37] Although we must be cautious not to mix our findings obtained using the modified oligonucleotides, with innate biology, our findings of intracellular trafficking of HDO might help to understand innate biology of the DNA/RNA heteroduplex.

Limitations

The results of our study are primarily based on lipid transfection experiments. We observed that gymnosis (free uptake) did not induce

an effective delivery in our experimental system (Figure S4). The observations of our study need to be generalized and applied to *in vivo* studies in the future for advancing to therapeutic application.

Conclusion

Here, we presented the intracellular trafficking of HDO. Understanding this unique mechanism will help us design more efficient drugs and also provide more insight into innate DNA/RNA cellular biology.

MATERIALS AND METHODS

Design and synthesis of oligonucleotides

A series of oligonucleotides were synthesized by Gene Design (Osaka, Japan). The sequences targeting intron *APOB* mRNA were as follows: ASO, 5′-c*a*t*c*c*c*a*c*c*a*c*a*t*a*g*c-3′; cRNA, 5′-G*C*U*AUGUGGUGGG*A*U*G-3′; full 2′-OMe CS, 5′-G*C*U*AUGUGGUGGG*A*U*G-3′. Lower case letters represent DNA, lower case underlined letters represent LNA (c denotes LNA 5-methylcytosine), upper case letters represent RNA, upper case underlined letters represent 2′-*O*-methyl sugar modification, and * indicates phosphorothioate linkage. The other sequences used were as follows: exon *APOB*: AS, 5′-g*c*a*t*t*g*g*t*a*t*t*c*a-3′; CS, 5′-U*G*A*AUACCAAU*G*C-3′.[38] *SNCA-1*: AS, 5′-c*c*a*t*t*c*c*c*a*a*g*a*g*a*c*c*c*a*g*a-3′; CS, 5′-U*C*U*G*G*GUCUCUUGGG*A*A*U*G*G-3′. *SNCA-2*: AS, 5′-a*g*a*a*g*a*a*t*c*a*a*t*t*g*c*t*t*t*a*c-3′; CS, 5′-G*U*A*A*A*GCAAUUGAUU*C*U*U*C*U-3′.[39] Scramble sequence (no target): AS, 5′-g*g*c*c*a*a*t*a*c*g*c*c*g*t*c*a-3′; CS, 5′-U*G*A* CGGCGUAUUG*G*C*C-3′.[40] siRNA targeting *GAPDH*: sense, GUAUGACAACAGCCUCAAGtt; antisense, CUUGAGGCU-GUUGUCAUACtt.[41] Full 2′-OMe CS targeting mouse *Malat*: G*C*A*UUCAGUGAAC*U*A*G. Alexa Fluor 488, Alexa Fluor 568, or Alexa Fluor 647 was covalently bound to the 5′ end of antisense or 3′ end of the CS, as mentioned in the text. Equimolar concentrations of antisense and complementary strands in nuclease-free water (Life Technologies, Carlsbad, CA, USA) were heated at 95°C for 5 min and annealed at room temperature (20°C–25°C) for over 1 h to generate HDO.

Cell Transfection

Huh-7 cells, cultured in DMEM containing 10% fetal bovine serum (FBS) and 1% penicillin/streptomycin (P/S) at 37°C and 5% CO_2, were seeded in 24-well plates at 25,000–50,000 cells/well (depending on the time frame of the assay) 12 h before transfection. Oligonucleotides in 100 μL Opti-MEM (Life Technologies) and 1 μL Lipofectamine RNAiMAX (Life Technologies) were mixed at room temperature for 20 min and then added to cells in 400 μL DMEM (FBS+ P/S−) and incubated for 4 h. Transfection medium was then replaced with fresh DMEM (FBS+ P/S+) for subsequent experiments.

In co-transfection experiments, two kinds of oligonucleotides were mixed initially in Opti-MEM, to which 1 μL Lipofectamine RNAiMAX was added. In gymnotic delivery experiments, oligonucleotides in 100 μL Opti-MEM were added to cells in 400 μL DMEM (FBS+ P/S−) and incubated for 24 h. We could not evaluate the gymnotic delivery longer than 24 h because cells died. Additionally, *ACTB* was used as the internal control in this experiment because we were afraid that a large number of oligos affected the expression of *GAPDH*. HEK293T cells were utilized only for the experiments presented in Figures S9 and S10.

Quantitative real-time PCR assay

Total RNA was extracted with Isogen II (Nippon Gene, Tokyo, Japan) and was reverse-transcribed with PrimeScript RT Master Mix (Takara Bio, Kusatsu, Japan). The cDNAs were amplified using the LightCycler 480 II (Roche Diagnostics, Rotkreuz, Switzerland). Gene expression values were calculated using the comparative delta *C*t method, normalized by the expression of the housekeeping gene, *GAPDH* or *ACTB*, as previously described.[5] TaqMan primers (Applied Biosystems, Life Technologies) used in this study were as follows: *APOB* (Hs00181142_m1), *GAPDH* (Hs99999905_m1), *SNCA* Hs01103383_m1), and *ACTB* (forward: 5′-CGGACTATGACTTAGTTGCGTTACA-3′; reverse: 5′-GCCATGCCAATCTCATCTTGT-3′; probe: 5′-FAM-CCTTTCTTGACAAAACCTAACTTGCGCAGA-TAMRA-3′).

Live-cell imaging

For live-cell imaging, Huh-7 cells were seeded in 35 mm Cell Imaging Dishes (Eppendorf, Hamburg, Germany) at 12,500 cells/well 12 h before transfection or transduction of labeled endosomes. In endosomal co-localization experiments, cells were transduced with GFP-labeled early or late endosomes 24 h before their transfection with oligonucleotides, using BacMam 2.0 CellLight Early endosome-GFP or Late endosome-GFP (Life Technologies), respectively, as previously described.[42] Cells were treated with LysoTracker Red DND-99 (Life Technologies) 1 h before the imaging, according to the manufacturer's instructions, to visualize lysosomes.

Transfection mixture containing dye-conjugated oligonucleotides and 1 μL Lipofectamine RNAiMAX in 100 μL Opti-MEM was prepared and stored at room temperature for 20 min. One minute after culture medium was replaced with the transfection mixture and 400 μL phenol red-free DMEM (FBS+ P/S−) (FluoroBrite, Life Technologies) on the stage of the microscope, time-lapse imaging was initiated. The medium was replaced with phenol red-free DMEM (FBS+ P/S+) 4 h after transfection, depending on the time frame of experiments.

Microscope settings

All images in this study were obtained using a live-cell imaging system (Nikon A1R laser scanning confocal microscopy, Nikon NIS elements AR software ver. 4.5, Tokyo, Japan) and stage top incubator (INUG2H-TIZSH, Tokai Hit, Fujinomiya, Japan). Instruments were warmed up, and cells and medium were incubated in the chamber for more than 1 h before imaging to stabilize the conditions in the chamber (37°C, 5% CO_2, and 100% humidity).

A preliminary experiment determined that the thickness of Huh-7 cells was less than 7 μm by *z*-section imaging at high magnitude. Therefore, the microscopic settings were adjusted accordingly to

enable the detection of all intracellular signals. In this setting (10× objective and 10 ocular lenses; pinhole 28 μm), the calculated thickness of optical sections was 11 μm, and time-lapse imaging was obtained at a single plane, which was focused using differential interference contrast (DIC) imaging at initiation and autoregulated by Nikon Perfect Focus System. Depending on each experiment, 3 × 3 to 4 × 4 tailing images of each fluoresce and DIC were acquired every 30–120 s for 120 min. Imaging parameters, like laser intensity and exposure time, were uniformly set in the same experiments.

Measuring imaging data

The fluorescence intensity of HDO in releasing vesicles, nucleus, and cytosol were analyzed using Nikon NIS elements AR software ver. 4.5 (Nikon). As mentioned earlier, HDO release was defined as the sudden increase of cytosolic AS signal, and a releasing vesicle was identified by a transient increase of AF647 signal in the vesicle (except AF488 signal in Figure S6C), which meant the cancellation of self-quenching.[24] We also defined the uniform and well-demarcated nuclear signals as nuclear transport and counted them accordingly.

Nuclear and plasma membranes were identified with DIC imaging and manually traced, blinded by other fluorescent signals. Lysosomes were identified when lysotracker signals localized. Intensity values were presented as absolute values, differences between actual signals and background levels, to compare the signals from the same dye.

In time-lapse imaging, the vesicles that released HDO first in individual cells were traced and mean signal intensity of each fluorophore was obtained. Relative values were calculated as previously described.[24] Namely, $t = 0$ was set just before the nuclear distribution started. Each fluorescence intensity was normalized to 0–1, with 0 being the background intensity and 1 being the highest intensity value for each object, in order to adjust various expression levels of each object. Here, we defined the background level as the mean intensity of an arbitrary no-cell area in the same image at the given time,[21,22] instead of the mean intensity of untransfected cells,[24] because we needed to eliminate the fluorescence caused by the dye-containing medium (note that the optical section was as thick as 11 μm in our settings). Exceptionally, in GFP-labeled early or late endosome experiments, cytosolic regions without the target protein expression were used as background.

RNase treatment assay

Various oligonucleotides (40 pmol), of which AS and CS were labeled with AF647 and AF488, respectively, were incubated with 1 ng of RNase A (QIAGEN, Hilden, Germany) or 10 units of RNase H (Takara Bio) at 37°C for 1 h and then loaded on 20% polyacrylamide gel. After electrophoresis in TBE, fluorescence of each dye was imaged using ChemiDoc Touch MP (Bio-Rad, Hercules, CA, USA).

Statistical Analysis

All experiments were performed at least three times. All data represent mean ± SEM, unless otherwise mentioned. Student's t tests were used to compare results obtained from different groups and/or conditions using Microsoft Excel. A p value of less than 0.05 was considered significantly significant.

SUPPLEMENTAL INFORMATION

Supplemental Information can be found online at https://doi.org/10.1016/j.omtn.2020.11.022.

ACKNOWLEDGMENTS

We thank Dr. Punit P. Seth (Ionis Pharmaceuticals) for helpful suggestions and meaningful comments. This research was supported by the Basic Science and Platform Technology Programs for Advanced Biological Medicine (20am0401006h0002) to T.Y. from the Japan Agency for Medical Research and Development (AMED; Tokyo, Japan); a JSPS KAKENHI Grant-in-Aid for Scientific Research (S) (17H06109) to T.Y. and T.N. and (A) (19H01016) to T.N. and T.Y. from the Ministry of Education, Culture, Sports, Science and Technology (MEXT) of Japan (Tokyo); and Takeda Pharmaceutical Company (2A308 to T.Y.).

AUTHOR CONTRIBUTIONS

D.O. conducted the experiments; D.O., K.A., F.S., K.Y., T.N., and T.Y. designed the experiments; D.O., K.A, and T.Y. wrote the paper.

DECLARATION OF INTERESTS

T.Y. collaborates with Daiichi Sankyo, Mitsubishi Tanabe Pharma, Ono Pharmaceutical, Rena Therapeutics, Takeda Pharmaceutical, Nanocarrier Pharmaceutical, and Toray Industries and serves as an academic adviser for Rena Therapeutics. All other authors declare no competing interests.

REFERENCES

1. Bennett, C.F., and Swayze, E.E. (2010). RNA targeting therapeutics: molecular mechanisms of antisense oligonucleotides as a therapeutic platform. Annu. Rev. Pharmacol. Toxicol. *50*, 259–293.

2. Kole, R., Krainer, A.R., and Altman, S. (2012). RNA therapeutics: beyond RNA interference and antisense oligonucleotides. Nat. Rev. Drug Discov. *11*, 125–140.

3. Raal, F.J., Santos, R.D., Blom, D.J., Marais, A.D., Charng, M.J., Cromwell, W.C., Lachmann, R.H., Gaudet, D., Tan, J.L., Chasan-Taber, S., et al. (2010). Mipomersen, an apolipoprotein B synthesis inhibitor, for lowering of LDL cholesterol concentrations in patients with homozygous familial hypercholesterolaemia: a randomised, double-blind, placebo-controlled trial. Lancet *375*, 998–1006.

4. Finkel, R.S., Chiriboga, C.A., Vajsar, J., Day, J.W., Montes, J., De Vivo, D.C., Yamashita, M., Rigo, F., Hung, G., Schneider, E., et al. (2016). Treatment of infantile-onset spinal muscular atrophy with nusinersen: a phase 2, open-label, dose-escalation study. Lancet *388*, 3017–3026.

5. Nishina, K., Piao, W., Yoshida-Tanaka, K., Sujino, Y., Nishina, T., Yamamoto, T., Nitta, K., Yoshioka, K., Kuwahara, H., Yasuhara, H., et al. (2015). DNA/RNA heteroduplex oligonucleotide for highly efficient gene silencing. Nat. Commun. *6*, 7969.

6. Yoshioka, K., Kunieda, T., Asami, Y., Guo, H., Miyata, H., Yoshida-Tanaka, K., Sujino, Y., Piao, W., Kuwahara, H., Nishina, K., et al. (2019). Highly efficient silencing of microRNA by heteroduplex oligonucleotides. Nucleic Acids Res. *47*, 7321–7332.

7. Kuwahara, H., Song, J., Shimoura, T., Yoshida-Tanaka, K., Mizuno, T., Mochizuki, T., Zeniya, S., Li, F., Nishina, K., Nagata, T., et al. (2018). Modulation of blood-brain barrier function by a heteroduplex oligonucleotide in vivo. Sci. Rep. *8*, 4377.

8. Crooke, S.T., Wang, S., Vickers, T.A., Shen, W., and Liang, X.H. (2017). Cellular uptake and trafficking of antisense oligonucleotides. Nat. Biotechnol. *35*, 230–237.

9. Juliano, R.L. (2016). The delivery of therapeutic oligonucleotides. Nucleic Acids Res. *44*, 6518–6548.

10. Juliano, R.L. (2018). Intracellular Trafficking and Endosomal Release of Oligonucleotides: What We Know and What We Don't. Nucleic Acid Ther. *28*, 166–177.

11. Crooke, S.T. (2017). Molecular Mechanisms of Antisense Oligonucleotides. Nucleic Acid Ther. *27*, 70–77.

12. Miller, C.M., Wan, W.B., Seth, P.P., and Harris, E.N. (2018). Endosomal Escape of ASOs Internalized by Stabilin Receptors Is Regulated by Rab5C and EEA1 During Endosomal Maturation. Nucleic Acid Ther. *28*, 86–96.

13. Linnane, E., Davey, P., Zhang, P., Puri, S., Edbrooke, M., Chiarparin, E., Revenko, A.S., Macleod, A.R., Norman, J.C., and Ross, S.J. (2019). Differential uptake, kinetics and mechanisms of intracellular trafficking of next-generation antisense oligonucleotides across human cancer cell lines. Nucleic Acids Res. *47*, 4375–4392.

14. Wang, S., Sun, H., Tanowitz, M., Liang, X.H., and Crooke, S.T. (2016). Annexin A2 facilitates endocytic trafficking of antisense oligonucleotides. Nucleic Acids Res. *44*, 7314–7330.

15. Liang, X.H., Sun, H., Nichols, J.G., and Crooke, S.T. (2017). RNase H1-Dependent Antisense Oligonucleotides Are Robustly Active in Directing RNA Cleavage in Both the Cytoplasm and the Nucleus. Mol. Ther. *25*, 2075–2092.

16. Zhao, M., Yang, H., Jiang, X., Zhou, W., Zhu, B., Zeng, Y., Yao, K., and Ren, C. (2008). Lipofectamine RNAiMAX: an efficient siRNA transfection reagent in human embryonic stem cells. Mol. Biotechnol. *40*, 19–26.

17. Nakamura, T., Kuroi, M., Fujiwara, Y., Warashina, S., Sato, Y., and Harashima, H. (2016). Small-sized, stable lipid nanoparticle for the efficient delivery of siRNA to human immune cell lines. Sci. Rep. *6*, 37849.

18. Wang, T., Larcher, L.M., Ma, L., and Veedu, R.N. (2018). Systematic screening of commonly used commercial transfection reagents towards efficient transfection of single-stranded oligonucleotides. Molecules *23*, 2564.

19. Liang, F., Pan, T., and Sevick-Muraca, E.M. (2005). Measurements of FRET in a Glucose-sensitive Affinity System with Frequency-domain Lifetime Spectroscopy. Photochem. Photobiol. *81*, 1386–1394.

20. Hirsch, M., Strand, D., and Helm, M. (2012). Dye selection for live cell imaging of intact siRNA. Biol. Chem. *393*, 23–35.

21. Hirsch, M., and Helm, M. (2015). Live cell imaging of duplex siRNA intracellular trafficking. Nucleic Acids Res. *43*, 4650–4660.

22. Järve, A., Müller, J., Kim, I.H., Rohr, K., MacLean, C., Fricker, G., Massing, U., Eberle, F., Dalpke, A., Fischer, R., et al. (2007). Surveillance of siRNA integrity by FRET imaging. Nucleic Acids Res. *35*, e124.

23. Raemdonck, K., Remaut, K., Lucas, B., Sanders, N.N., Demeester, J., and De Smedt, S.C. (2006). In situ analysis of single-stranded and duplex siRNA integrity in living cells. Biochemistry *45*, 10614–10623.

24. Wittrup, A., Ai, A., Liu, X., Hamar, P., Trifonova, R., Charisse, K., Manoharan, M., Kirchhausen, T., and Lieberman, J. (2015). Visualizing lipid-formulated siRNA release from endosomes and target gene knockdown. Nat. Biotechnol. *33*, 870–876.

25. Tansi, F.L., Rüger, R., Rabenhold, M., Steiniger, F., Fahr, A., Kaiser, W.A., and Hilger, I. (2013). Liposomal encapsulation of a near-infrared fluorophore enhances fluorescence quenching and reliable whole body optical imaging upon activation in vivo. Small *9*, 3659–3669.

26. ur Rehman, Z., Hoekstra, D., and Zuhorn, I.S. (2013). Mechanism of polyplex- and lipoplex-mediated delivery of nucleic acids: real-time visualization of transient membrane destabilization without endosomal lysis. ACS Nano *7*, 3767–3777.

27. Sahay, G., Querbes, W., Alabi, C., Eltoukhy, A., Sarkar, S., Zurenko, C., Karagiannis, E., Love, K., Chen, D., Zoncu, R., et al. (2013). Efficiency of siRNA delivery by lipid nanoparticles is limited by endocytic recycling. Nat. Biotechnol. *31*, 653–658.

28. Rigby, R.E., Webb, L.M., Mackenzie, K.J., Li, Y., Leitch, A., Reijns, M.A., Lundie, R.J., Revuelta, A., Davidson, D.J., Diebold, S., et al. (2014). RNA:DNA hybrids are a novel molecular pattern sensed by TLR9. EMBO J. *33*, 542–558.

29. Kailasan Vanaja, S., Rathinam, V.A.K., Atianand, M.K., Kalantari, P., Skehan, B., Fitzgerald, K.A., and Leong, J.M. (2014). Bacterial RNA:DNA hybrids are activators of the NLRP3 inflammasome. Proc. Natl. Acad. Sci. USA *111*, 7765–7770.

30. Liu, B., Hu, J., Wang, J., and Kong, D. (2017). Direct visualization of RNA-DNA primer removal from okazaki fragments provides support for flap cleavage and exonucleolytic pathways in eukaryotic cells. J. Biol. Chem. *292*, 4777–4788.

31. Neil, A.J., Liang, M.U., Khristich, A.N., Shah, K.A., and Mirkin, S.M. (2018). RNA-DNA hybrids promote the expansion of Friedreich's ataxia (GAA)n repeats via break-induced replication. Nucleic Acids Res. *46*, 3487–3497.

32. Nick McElhinny, S.A., Kumar, D., Clark, A.B., Watt, D.L., Watts, B.E., Lundström, E.B., Johansson, E., Chabes, A., and Kunkel, T.A. (2010). Genome instability due to ribonucleotide incorporation into DNA. Nat. Chem. Biol. *6*, 774–781.

33. Crossley, M.P., Bocek, M., and Cimprich, K.A. (2019). R-Loops as Cellular Regulators and Genomic Threats. Mol. Cell *73*, 398–411.

34. Maizels, N. (2006). Dynamic roles for G4 DNA in the biology of eukaryotic cells. Nat. Struct. Mol. Biol. *13*, 1055–1059.

35. Koo, C.X.G.E., Kobiyama, K., Shen, Y.J., LeBert, N., Ahmad, S., Khatoo, M., Aoshi, T., Gasser, S., and Ishii, K.J. (2015). RNA polymerase III regulates cytosolic RNA:DNA hybrids and intracellular microRNA expression. J. Biol. Chem. *290*, 7463–7473.

36. Asada, K., Ito, K., Yui, D., Tagaya, H., and Yokota, T. (2018). Cytosolic Genomic DNA functions as a Natural Antisense. Sci. Rep. *8*, 8551.

37. Balaj, L., Lessard, R., Dai, L., Cho, Y.J., Pomeroy, S.L., Breakefield, X.O., and Skog, J. (2011). Tumour microvesicles contain retrotransposon elements and amplified oncogene sequences. Nat. Commun. *2*, 180–189.

38. Straarup, E.M., Fisker, N., Hedtjärn, M., Lindholm, M.W., Rosenbohm, C., Aarup, V., Hansen, H.F., Ørum, H., Hansen, J.B.R., and Koch, T. (2010). Short locked nucleic acid antisense oligonucleotides potently reduce apolipoprotein B mRNA and serum cholesterol in mice and non-human primates. Nucleic Acids Res. *38*, 7100–7111.

39. Bennett, F., Freier, S., and Mallajosyula, J. (2012). MODULATION OF ALPHA SYNUCLEN EXPRESSION. International patent WO/2012/068405A2, filed 17 Nov 2011 / published 24 May 2012.

40. Murray, S.F., Seth, P.P., Mccaleb, M.L., Freier, S.M., and Singh, P. (2016). ALLELE SPECIFIC MODULATORS OF P23H RHODOPSIN. International patent WO/2016/138353A1, filed 26 Feb 2016 / published 1 Sep 2016.

41. Li, B.P., Liu, J.L., Chen, J.Q., Wang, Z., Mao, Y.T., and Chen, Y.Y. (2015). Effects of siRNA-mediated silencing of myeloid cell leukelia-1 on the biological behaviors and drug resistance of gastric cancer cells. Am. J. Transl. Res. *7*, 2397–2411.

42. Lazebnik, M., and Pack, D.W. (2017). Rapid and facile quantitation of polyplex endocytic trafficking. J. Control. Release *247*, 19–27.

OMTN, Volume *23*

Supplemental information

Separation-related rapid nuclear transport of DNA/RNA heteroduplex oligonucleotide: unveiling distinctive intracellular trafficking

Daisuke Ono, Ken Asada, Daishi Yui, Fumika Sakaue, Kotaro Yoshioka, Tetsuya Nagata, and Takanori Yokota

Supplemental figures and legends

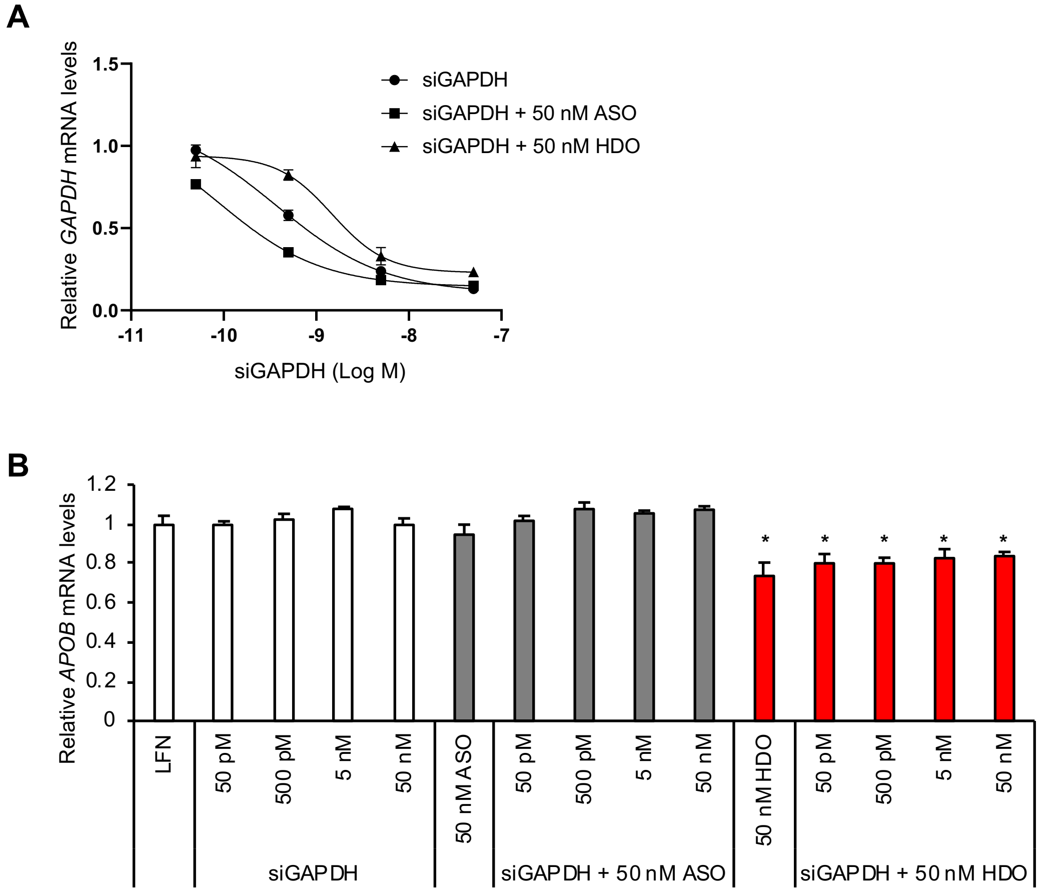

Figure S1. Co-Transfection of ASO/HDO with siGAPDH to Control the Transfection Efficiency of ASO and HDO
To evaluate and control transfection efficiency of different oligonucleotides, 50 nM ASO or HDO targeting intron *APOB* were co-transfected with various doses of control siRNA targeting a housekeeping gene; *GAPDH*. 24 h after transfection, *APOB* and *GAPDH* mRNA levels were quantified using RT-qPCR, normalized to those of *ACTB* and "no siGAPDH" control in each group. In the experiments, we aimed to calculate 50% inhibitory concentration (IC_{50}) of siRNA, at which, we could compare the intracellular efficacy of co-transfected ASO and HDO by equalizing co-transfected siGAPDH activity. (A) The inhibitory dose-responsive curve was fitted with the *GAPDH* mRNA levels at various doses of siGAPDH. IC_{50} of single transfection with siGAPDH, co-transfection of siGAPDH with 50 nM ASO, and 50 nM HDO were calculated as 370 pM, 88 pM, and 1.4 nM, respectively. (B) RT-qPCR analysis of *APOB* levels 24 h after co-transfection. At around IC_{50} of siGAPDH (50 pM ~ 5 nM), comparable doses of HDO significantly downregulated target *APOB* mRNA, whereas same doses of ASO did not. Note that Figures S1A and S1B were based on data from the simultaneous experiment (* $p < 0.05$, vs lipofectamine (LFN) control; n = 3; mean ± SEM).

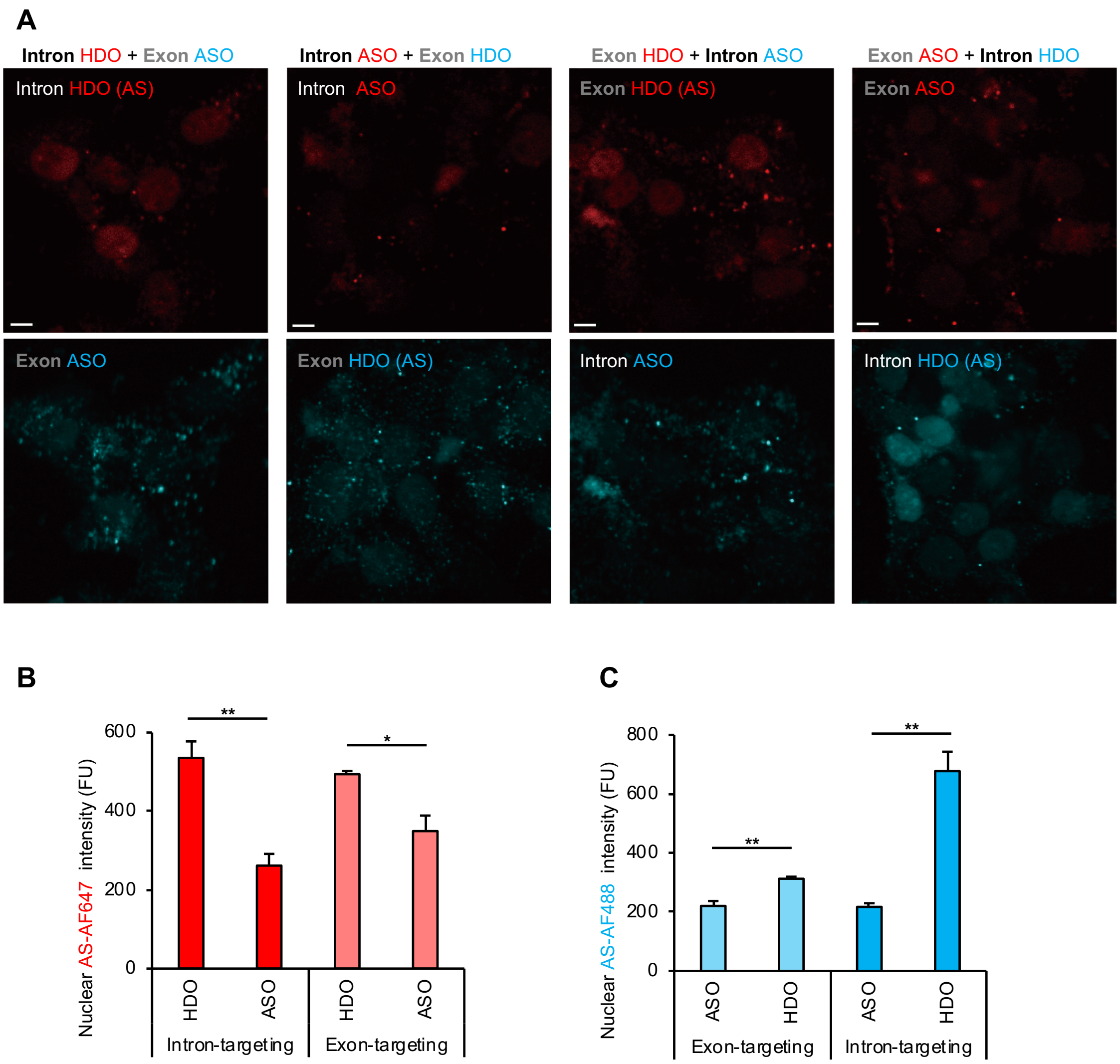

Figure S2. Co-Transfection with ASO and HDO

To compare the nuclear distribution equalizing transfection amount, we performed co-transfection with dye-labeled ASO and HDO, and imaged them 24 h after transfection, where we used two antisense sequence (16-mer targeting intron *APOB* and 13-mer targeting exon *APOB*) so that complementary strand (CS) of HDO did not hybridize with the other ASO. We labeled antisense strand (AS) of HDO and ASO with AF647 and AF488. These two sequences for HDO or ASO, and their labels were switched to control the effects of sequences and dyes. (A) Representative images 24 h after co-transfection of 50 nM ASO and HDO. AF-647 signals (red) were excited by a 646 nm laser and detected through a 700 (663-738) nm filter. AF-488 signals (cyan) were excited by a 488 nm laser and detected through a 525 (500-550) nm filter. Bar = 10 µm. (B) Mean nuclear intensities presented as absolute values normalized to background levels. More signals of HDO were detected in nuclei than those of ASO after controlling effects of sequences and dyes (* $p < 0.05$, ** $p < 0.01$; n = 3 images for each 50 cells; mean ± SEM).

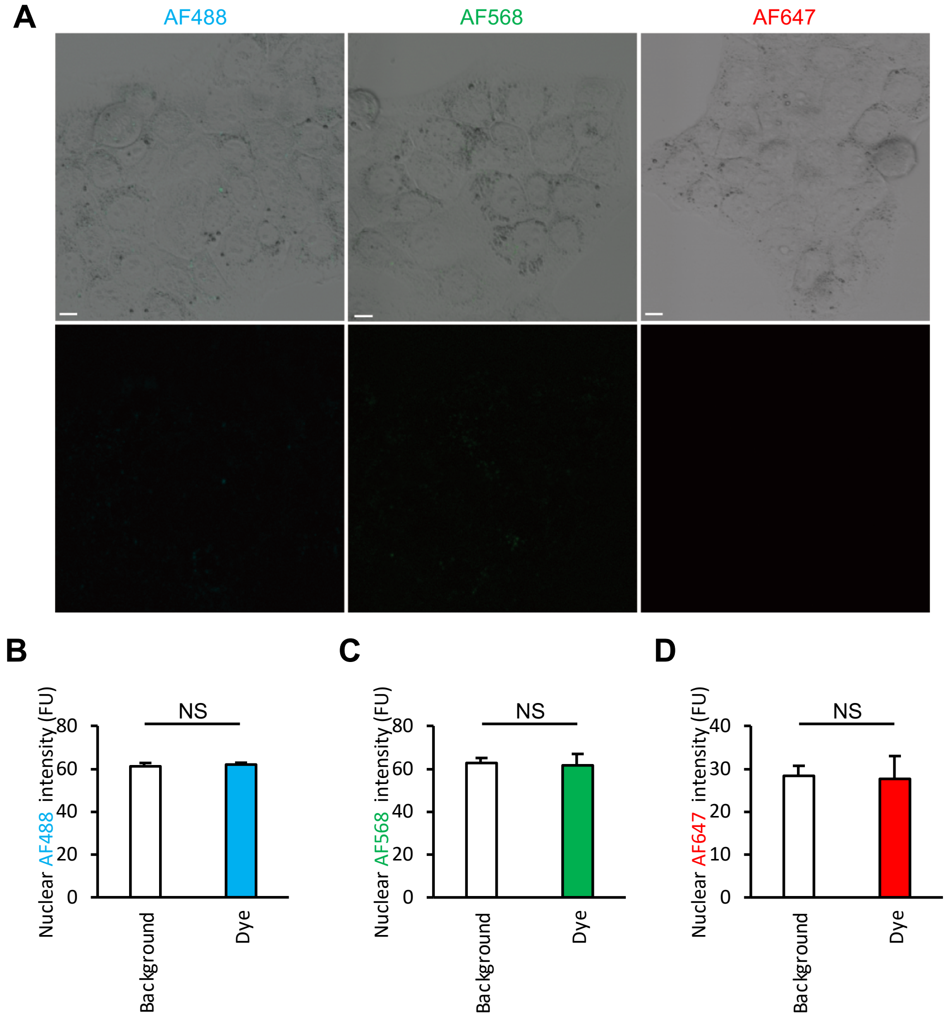

Figure S3. Transfection of Dyes

(A) Representative images 24 h after transfection with 50 nM of each dye. The upper row shows merged images of differential interference contrast (DIC) and each fluorescent signal (left, AF488; center, AF568; right, AF647). The lower row shows each fluorescence image. AF488 signals (cyan) were excited by a 488 nm laser and detected through a 525 (500-550) nm filter. AF568 signals (green) were excited by a 560 nm laser and detected through a 595 (570-620) nm filter. AF647 signals (red) were excited by a 646 nm laser and detected through a 700 (663-738) nm filter. Bar = 10 µm. (B) Mean nuclear intensities of dyes and background levels presented as absolute values (NS, not significant; n = 3 images for every 50 cells; mean ± SEM).

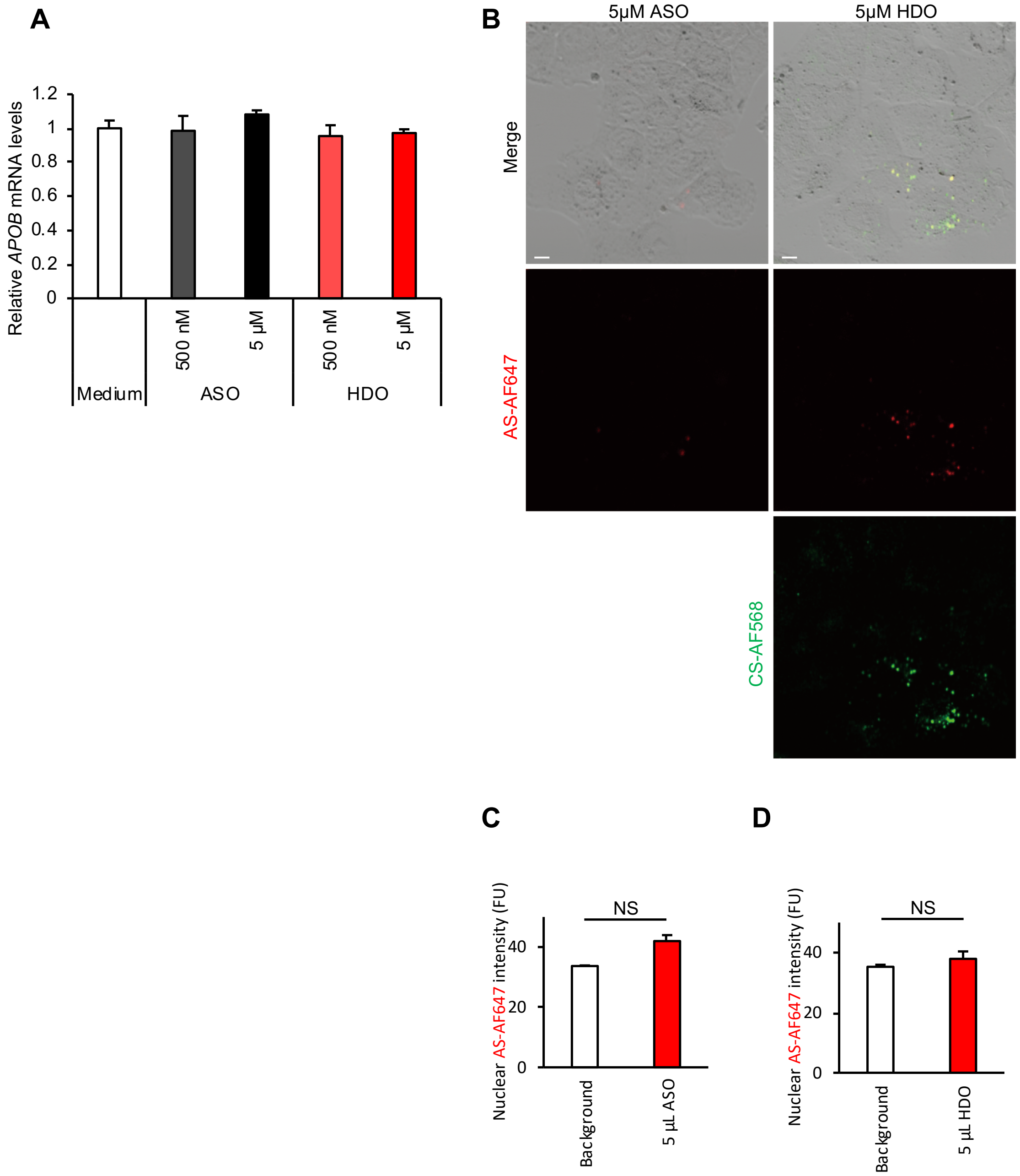

Figure S4. Gymnotic Delivery of ASO and HDO

To evaluate other delivery method than lipid transfection, we performed gymnotic (free uptake) delivery. (A) RT-qPCR analysis of *APOB* levels, normalized to *ACTB* levels 24 h after gymnotic delivery with ASO or HDO targeting intron *APOB* (no significance was found vs control medium; n = 3; mean ± SEM). (B) Representative images 24 h after transfection with 5 µM of ASO or HDO targeting intron *APOB*. Only 1% of oligos were labeled to avoid the non-specific hyperintensity of the background signal. AF647 signals (red) were excited by a 646 nm laser and detected through a 700 (663-738) nm filter. AF568 signals (green) were excited by a 560 nm laser and detected through a 595 (570-620) nm filter. Bar = 10 µm. (C, D) Mean nuclear intensities of AS-AF647 with 5 µM of ASO (C) and HDO (D) presented as absolute values (no significance (NS) was found vs background levels; n = 3 images for every 50 cells; mean ± SEM).

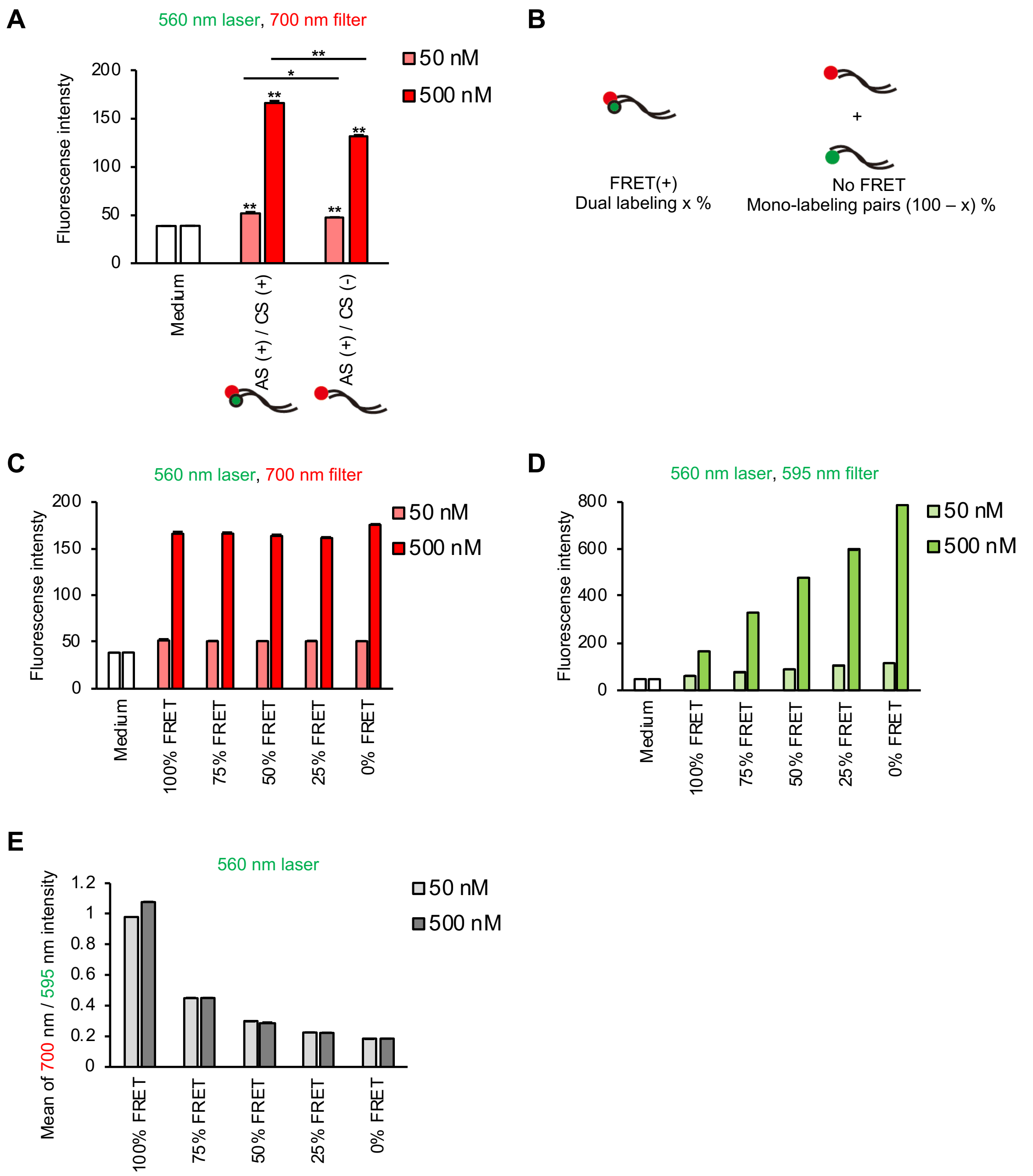

Figure S5. Confirmation of FRET System

To confirm the FRET system which we utilized in the experiments, we measured the fluorescence intensity of dye-conjugated HDO in solution without cell or transfection reagent. (A) 50 nM or 500 nM HDO targeting intron *APOB*, composed of AF647-labeled antisense strand (AS)/AF568-labeled complementary strand (CS) [AS (+) / CS(+)] or AF647-labeled AS/CS without dye [AS (+) / CS(-)], were excited by a 560 nm laser, detected through a 700 (663-738) nm filter (* $p < 0.05$, ** $p < 0.01$ vs medium control if not indicated by bars; $n = 3$; mean absolute value ± SEM). In this setting, the difference between AS (+)/CS(+) and AS (+)/CS(-) means FRET signal. The difference between AS (+)/CS(-) and medium means non-specific AF-647 signal excited by a 560 nm laser, which was more intense than FRET signals, and made it difficult to isolate FRET signals. (B-E) In the HDO separation site, where winding HDO and separated AS-AF647 exist densely, we could not directly identify acceptor signal of FRET from non-specific signals of isolated AF647 excited by 560 nm laser. To solve this problem, we mixed FRET (+) dual labeled HDO and no FRET single-labeled pairs of HDO at various rates, and measured solution intensity, which models the winding and/or separated form of double-stranded oligos[21] ($n = 3$; mean absolute value ± SEM). Signals excited by a 560 nm laser, through a 700 (663-738) nm filter were not remarkably different between FRET (+) model and no FRET model (C). On the contrary, a remarkable increase of the signal excited by a 560 nm laser and detected through a 595 (570-620) nm filter was observed with an increase of no FRET mono-labeling pairs, which corresponded to the dequenching signal of the donor dyes (D). To control the difference in concentration, we also calculated the ratio[21]—the signal intensity from the 700 nm filter divided by that from 595 nm filter—both of which were excited by a 560 nm laser and normalized to medium intensity (E). This ratio was correlated to FRET regardless of HDO concentration.

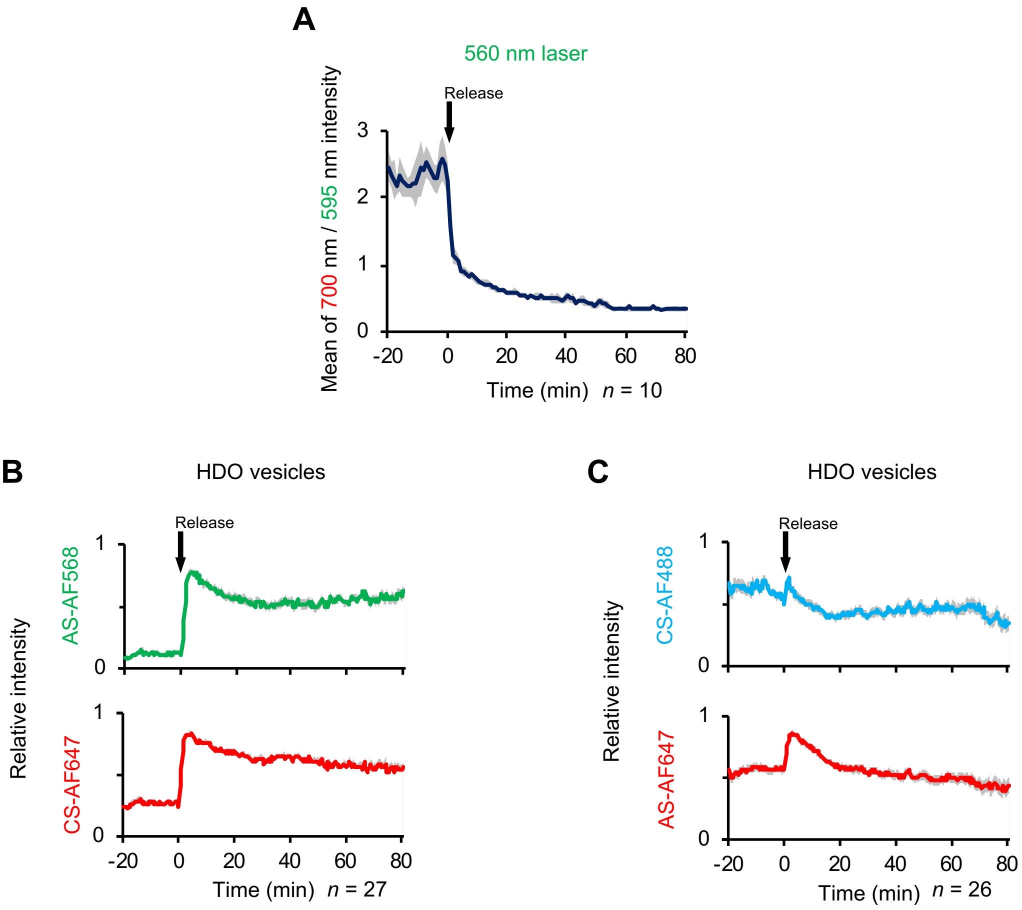

Figure S6. Confirmation of HDO Separation in HDO-Releasing Vesicles
(A) To detect FRET, signals through 595 (570-620) nm and 700 (663-738) nm filters excited by a 560 nm laser in HDO-releasing vesicles were measured after transfection with 50 nM HDO targeting intron *APOB*, composed of antisense strand (AS)-AF647 and complementary strand (CS)-AF568. The ratio—the mean signal intensity from the 700 nm filter divided by that from 595 nm filter—was sequentially calculated, as presented in Figure S5. Sudden decrease of the ratio at the time of the release was confirmed to be the cancellation of the FRET signals. (B) To exclude the possibility that these signal changes were not as a result of a particular dye-nucleotide interaction, AS was reversely labeled with AF568 and CS with AF647. AF647 signals (red) were excited by a 646 nm laser and detected through a 700 (663-738) nm filter. AF568 signals (green) were excited by a 560 nm laser and detected through a 595 (570-620) nm filter. Dequenching signals of AS-AF568 and cancellation of self-quenching of CS-AF647 were observed just after the time of the release. (C) To confirm that FRET does not occur in AF647 and AF488 pairs and evaluate cancellation of self-quenching, AS was labeled with AF647 and CS with AF488. AF647 signals (red) were excited by a 646 nm laser and detected through a 700 (663-738) nm filter. AF488 signals (in cyan) were excited by a 488 nm laser and detected through a 525 (500-550) nm filter. Sequential signal changes of HDO-releasing vesicles are presented. Images were taken just after transfection with 50 nM HDO, every 1 min (A), or 30 sec (B, C). $t = 0$ is set just before the release started. (n = 10 (B), 27 (B), 26 (C); ± SEM, shaded areas). Results were pooled from three experiments per condition (B, C).

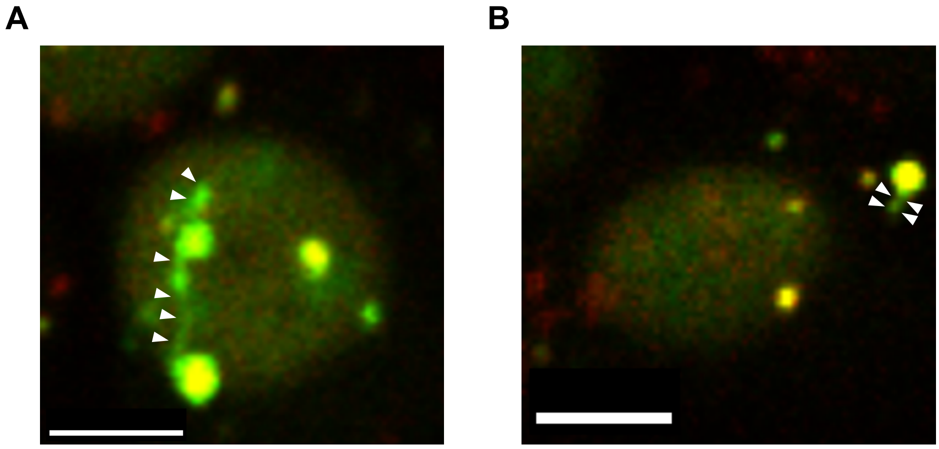

Figure S7. Inflow Signals from HDO-Releasing Vesicles

(A, B) Representative figures of inflow signals (arrowheads) from HDO-releasing vesicles in Movie 2A, B, respectively. Images were taken in a high sensitivity setting compared to the experiments in Figure 2, just after transfection with 50 nM HDO targeting intron *APOB*, composed of antisense strand-AF647 (red) and complementary strand-AF568 (green). AF647 signals were excited by a 646 nm laser and detected through a 700 (663-738) nm filter. AF568 signals were excited by a 560 nm laser and detected through a 595 (570-620) nm filter. Bar = 10 µm.

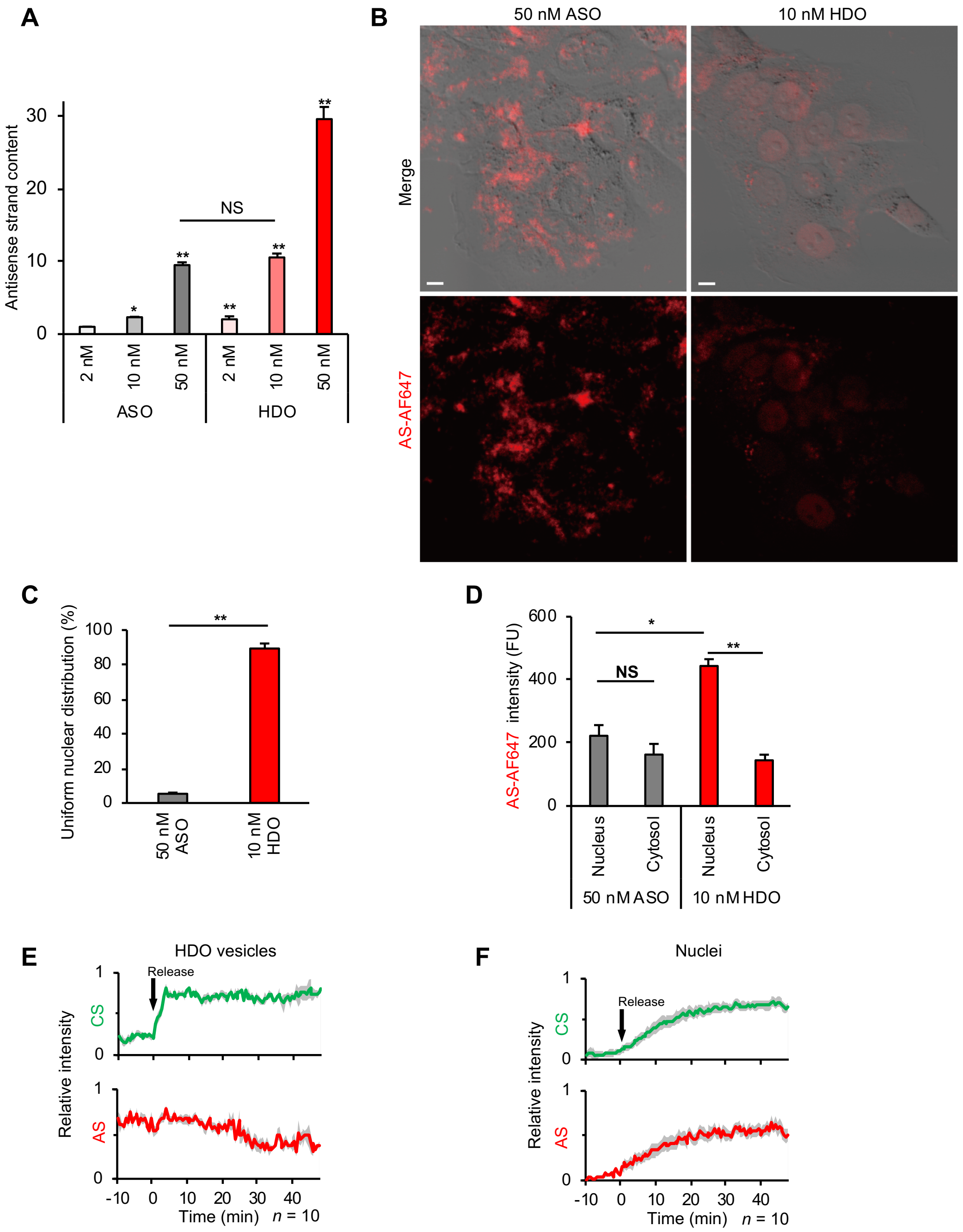

Figure S8. Evaluation and Equalization of Transfection Efficiency of HDO and ASO

To evaluate transfection efficiency of ASO and HDO, we measured antisense strand levels in cells, 24 h after transfection with various doses of ASO or HDO targeting intron *APOB*, and then determined the comparable dose of HDO to that of ASO as shown in Figures 1, 2. (A) RT-qPCR analysis of antisense strand content normalized to the levels of *U6* RNA 24 h after transfection with various doses of ASO or HDO, targeting intron *APOB* mRNA (* $p < 0.05$, ** $p < 0.01$ vs 2nM ASO; n = 3; mean ± SEM). The results showed that 10 nM HDO was comparable to 50 nM ASO. At these doses, 10 nM HDO significantly downregulated *APOB* mRNA but 50 nM ASO did not (Figure 1B). (B) Representative images 24 h after transfection with 50 nM ASO and 10 nM HDO. The upper row shows merged images of differential interference contrast (DIC) and antisense strand (AS)-AF647 (red). The lower row shows AS-AF64. AF647 signals were excited by a 646 nm laser and detected through a 700 (663-738) nm filter. Bar = 10 µm. (C) Percentage of cells with uniform nuclear distribution 24 h after transfection with 50 nM ASO or 10 nM HDO (** $p < 0.01$; n = 3 images for every 50 cells). (D) Mean intensities of AS-AF647 in the nucleus or cytosol 24 h after transfection with 50 nM ASO or 10 nM HDO, presented as absolute values normalized to the background signal. (* $p < 0.05$, ** $p < 0.01$; NS, not significant; n = 3 images for every 50 cells). (E, F) Time-lapse images were taken every 30 seconds after transfection with 10 nM HDO, and sequential signal changes of AS-AF647 (red) and complementary strand (CS)-AF568 (green) in HDO-releasing vesicles (E) and nuclei (F) were measured. Separation-related nuclear distribution of both strands just after cytosolic release was observed in 10 nM HDO similar to 50 nM. AF647 signals were excited by a 646 nm laser and detected through a 700 (663-738) nm filter. AF568 signals were excited by a 560 nm laser and detected through a 595 (570-620) nm filter. $t = 0$ is set just before the releases started. Mean intensities of each region were presented as relative values (0-1), with 0 being the background intensity, and 1 being the highest intensity value for each object (n = 10; ± SEM, shaded areas).

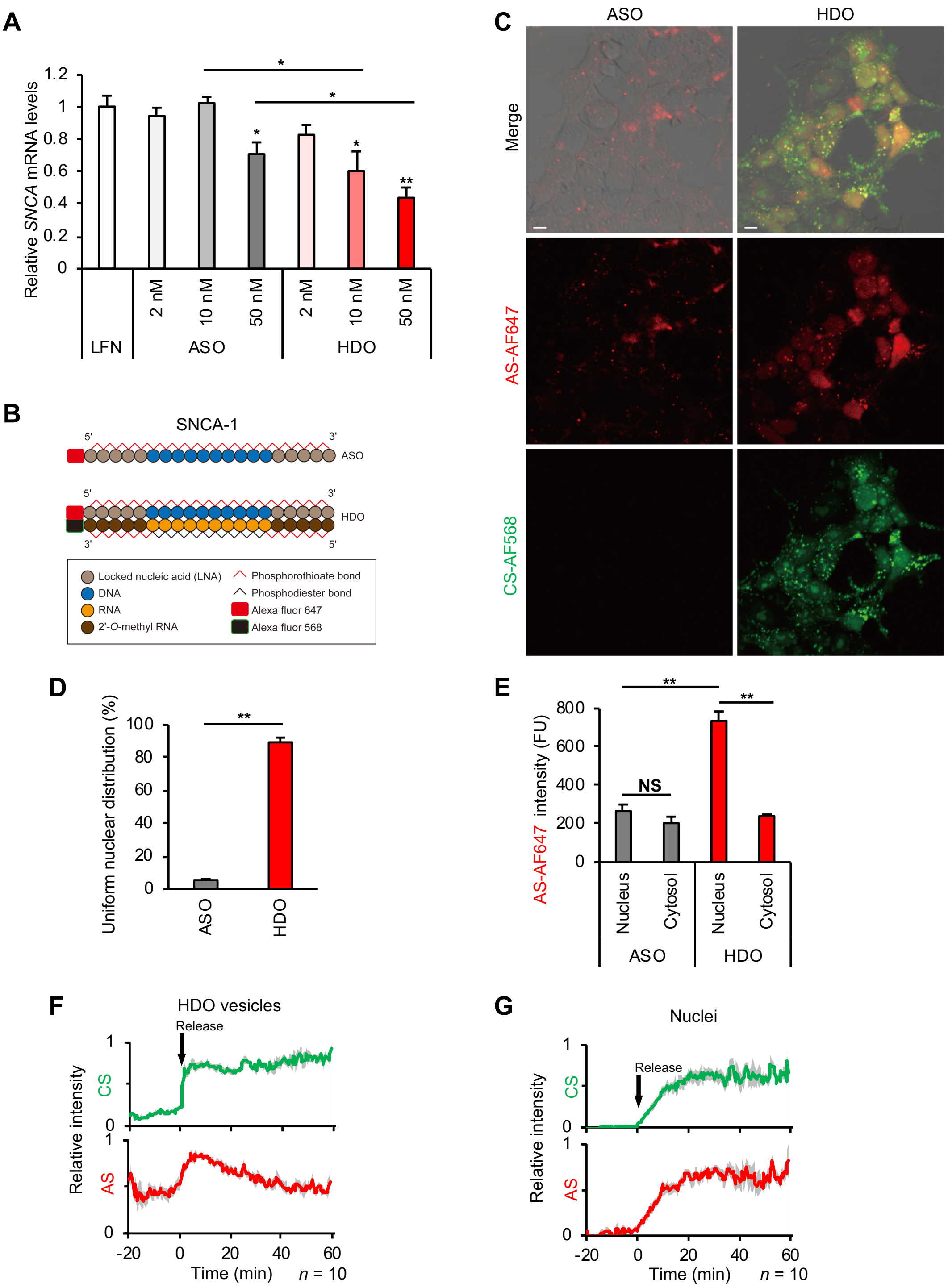

Figure S9. Separation-Related Nuclear Distribution of HDO by SNCA-1 Sequence in HEK 293T Cells
To confirm the separation-related nuclear distribution of HDO by another target, we performed the same experiments as shown in Figures 1, 2, by transfecting with ASO or HDO targeting intron region of *SNCA* (Alpha-Synuclein). Because *SNCA* was not expressed in Huh 7 cells, HEK 293T cells were transfected with Lipofectamine RNAiMAX. (A) RT-qPCR analysis of relative *SNCA* mRNA levels 24 h after transfection, normalized to *GAPDH* (* $p < 0.05$, ** $p < 0.01$ vs. lipofectamine (LFN) control; n = 3; mean ± SEM). (B) Designs of dye-conjugated ASO and HDO, targeting intron region of *SNCA* (SNCA-1 sequence), where complementary strand (CS) was labeled with AF568, and antisense strand (AS) was labeled with AF647. (C) Representative images 24 h after transfection with 50 nM ASO and HDO. The upper row shows merged images of differential interference contrast (DIC), AS-AF647 (red) and CS-AF568 (green). AF647 signals were excited by a 646 nm laser and detected through a 700 (663-738) nm filter. AF568 signals were excited by a 560 nm laser and detected through a 595 (570-620) nm filter. Bar = 10 µm. (D) Percentage of cells with uniform nuclear distribution 24 h after transfection with 50 nM ASO or HDO (** $p < 0.01$; n = 3 images for every 50 cells). (E) Mean intensities of AS-AF647 in the nucleus or cytosol 24 h after transfection with 50 nM ASO or HDO, presented as absolute values normalized to the background signal. (** $p < 0.01$; NS, not significant; n = 3 images for every 50 cells). (F, G) Time-lapse images were taken every 30 seconds after transfection with 50 nM HDO, and sequential changes of AS-AF647 and CS-AF568 signals in HDO-releasing vesicles (F) and nuclei (G) were measured. Separation-related nuclear distribution of both strands just after cytosolic release was observed. AF647 signals were excited by a 646 nm laser and detected through a 700 (663-738) nm filter. AF568 signals were excited by a 560 nm laser and detected through a 595 (570-620) nm filter. *t* = 0 is set just before the releases started. Mean intensities of each region were presented as relative values (0-1), with 0 being the background intensity, and 1 being the highest intensity value for each object (n = 10; ± SEM, shaded areas).

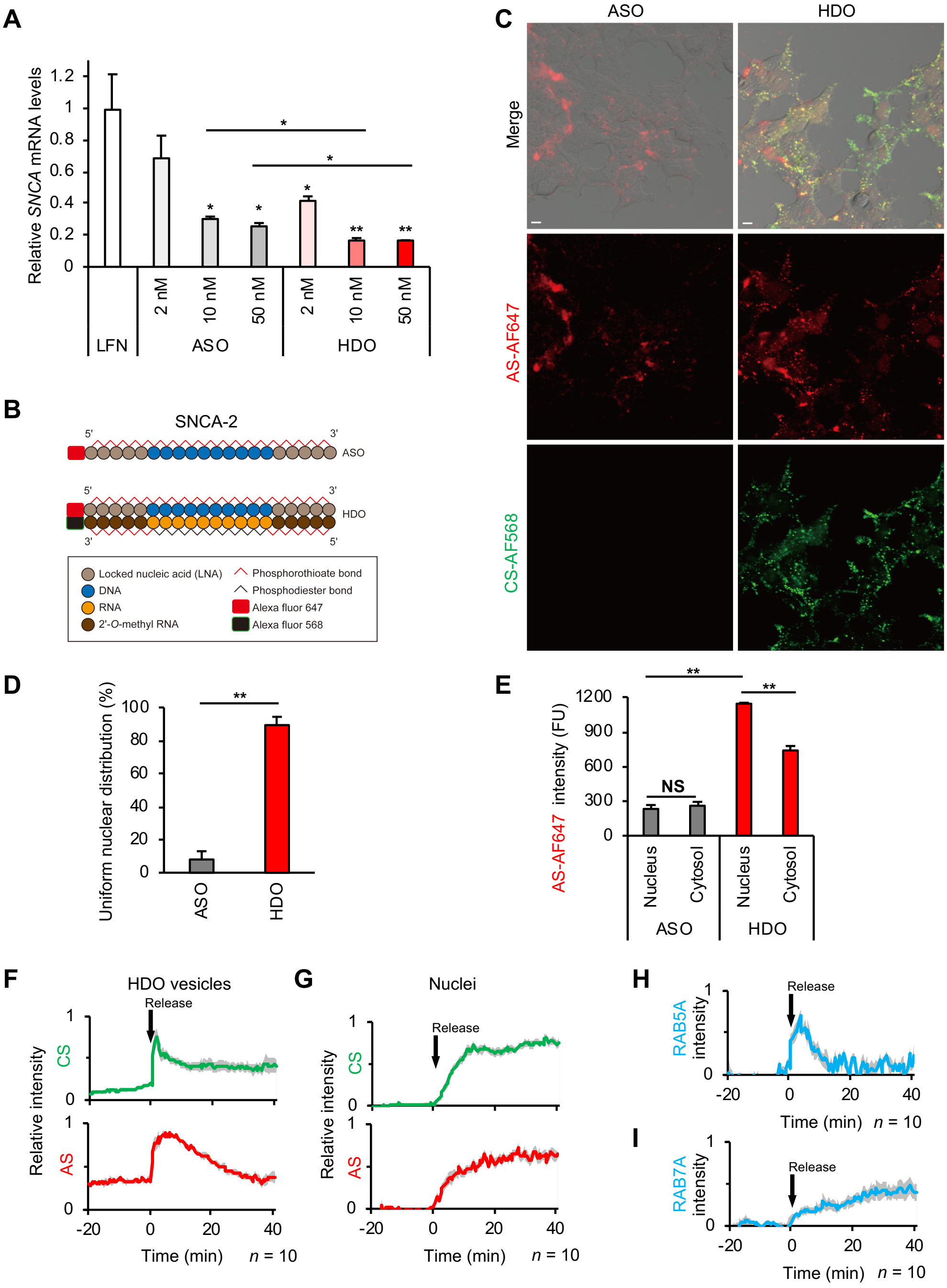

Figure S10. Intracellular Mechanism of HDO by Highly Efficient SNCA-2 Sequence

To evaluate the intracellular mechanism of HDO by a highly efficient sequence, we designed another SNCA-2 sequence targeting intron region of *SNCA* (Alpha-Synuclein). HEK 293T cells were transfected with Lipofectamine RNAiMAX. (A) RT-qPCR analysis of relative *SNCA* mRNA levels 24 h after transfection, normalized to *GAPDH* (* $p < 0.05$, ** $p < 0.01$ vs. lipofectamine (LFN) control; n = 3; mean ± SEM). 50% inhibitory concentration (IC_{50}) of ASO and HDO of SNCA-2 sequence were calculated as 3.7 nM and 2.2 nM, respectively. (B) Designs of dye-conjugated ASO and HDO, targeting intron region of *SNCA* , where complementary strand (CS) was labeled with AF568, and antisense strand (AS) was labeled with AF647. (C) Representative images 24 h after transfection with 50 nM ASO and HDO. The upper row shows merged images of differential interference contrast (DIC), AS-AF647 (red) and CS-AF568 (green). AF647 signals were excited by a 646 nm laser and detected through a 700 (663-738) nm filter. AF568 signals were excited by a 560 nm laser and detected through a 595 (570-620) nm filter. Bar = 10 µm. (D) Percentage of cells with uniform nuclear distribution 24 h after transfection with 50 nM ASO or HDO (** $p < 0.01$; n = 3 images for every 50 cells). (E) Mean intensities of AS-AF647 in the nucleus or cytosol 24 h after transfection with 50 nM ASO or HDO, presented as absolute values normalized to the background signal. (** $p < 0.01$; NS, not significant; n = 3 images for every 50 cells). (F, G) Time-lapse images were taken every 30 seconds after transfection with 50 nM HDO, and sequential changes of AS-AF647 and CS-AF568 signals in HDO-releasing vesicles (F) and nuclei (G) were measured. Separation-related nuclear distribution of both strands just after cytosolic release was observed. (H, I) Cells expressing GFP-labeled RAB5A (H) or RAB7A (I) were transfected with 50 nM HDO, and imaged every 30 seconds. Sequential changes of RAB5A (H) or RAB7A (I) signals in the HDO-releasing vesicles were measured. GFP signals (cyan)were excited by a 488 nm laser and detected through a 525 (500-550) nm filter. AF647 signals (red) were excited by a 646 nm laser and detected through a 700 (663-738) nm filter. AF568 signals (green) were excited by a 560 nm laser and detected through a 595 (570-620) nm filter. $t = 0$ is set just before the releases started. Mean intensities of each region were presented as relative values (0-1), with 0 being the background intensity, and 1 being the highest intensity value for each object (n = 10; ± SEM, shaded areas).

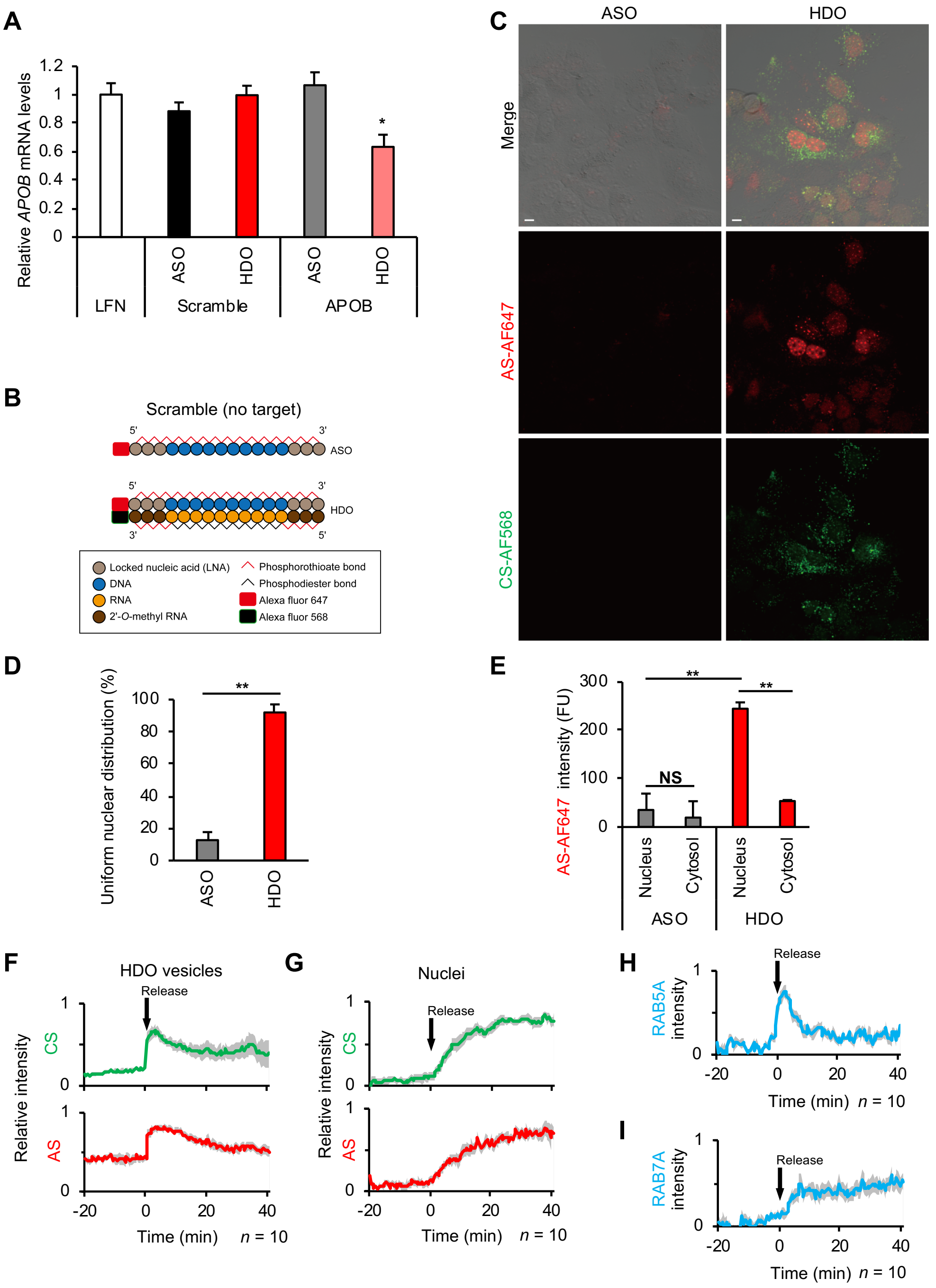

Figure S11. Intracellular Mechanism of HDO by a Scramble Sequence without any Target Gene

To confirm that our observation about intracellular mechanism of HDO are applicable indifferently to efficiency of certain sequence, we designed a scramble sequence without any target gene. Huh7 cells were transfected with Lipofectamine RNAiMAX. (A) RT-qPCR analysis of relative *APOB* mRNA levels 24 h after transfection, normalized to *GAPDH* (* $p < 0.05$ vs. lipofectamine (LFN) control; n = 3; mean ± SEM). (B) Designs of dye-conjugated ASO and HDO without any target gene, where complementary strand (CS) was labeled with AF568, and antisense strand (AS) was labeled with AF647. (C) Representative images 24 h after transfection with 50 nM ASO and HDO. The upper row shows merged images of differential interference contrast (DIC), AS-AF647 (red) and CS-AF568 (green). AF647 signals were excited by a 646 nm laser and detected through a 700 (663-738) nm filter. AF568 signals were excited by a 560 nm laser and detected through a 595 (570-620) nm filter. Bar = 10 µm. (D) Percentage of cells with uniform nuclear distribution 24 h after transfection with 50 nM ASO or HDO (** $p < 0.01$; n = 3 images for every 50 cells). (E) Mean intensities of AS-AF647 in the nucleus or cytosol 24 h after transfection with 50 nM ASO or HDO, presented as absolute values normalized to the background signal. (** $p < 0.01$; NS, not significant; n = 3 images for every 50 cells). (F, G) Time-lapse images were taken every 30 seconds after transfection with 50 nM HDO, and sequential changes of AS-AF647 and CS-AF568 signals in HDO-releasing vesicles (F) and nuclei (G) were measured. Separation-related nuclear distribution of both strands just after cytosolic release was observed. (H, I) Cells expressing GFP-labeled RAB5A (H) or RAB7A (I) were transfected with 50 nM HDO, and imaged every 30 seconds. Sequential changes of RAB5A (H) or RAB7A (I) signals in the HDO-releasing vesicles were measured. GFP signals (cyan)were excited by a 488 nm laser and detected through a 525 (500-550) nm filter. AF647 signals (red) were excited by a 646 nm laser and detected through a 700 (663-738) nm filter. AF568 signals (green) were excited by a 560 nm laser and detected through a 595 (570-620) nm filter. $t = 0$ is set just before the releases started. Mean intensities of each region were presented as relative values (0-1), with 0 being the background intensity, and 1 being the highest intensity value for each object (n = 10; ± SEM, shaded areas).

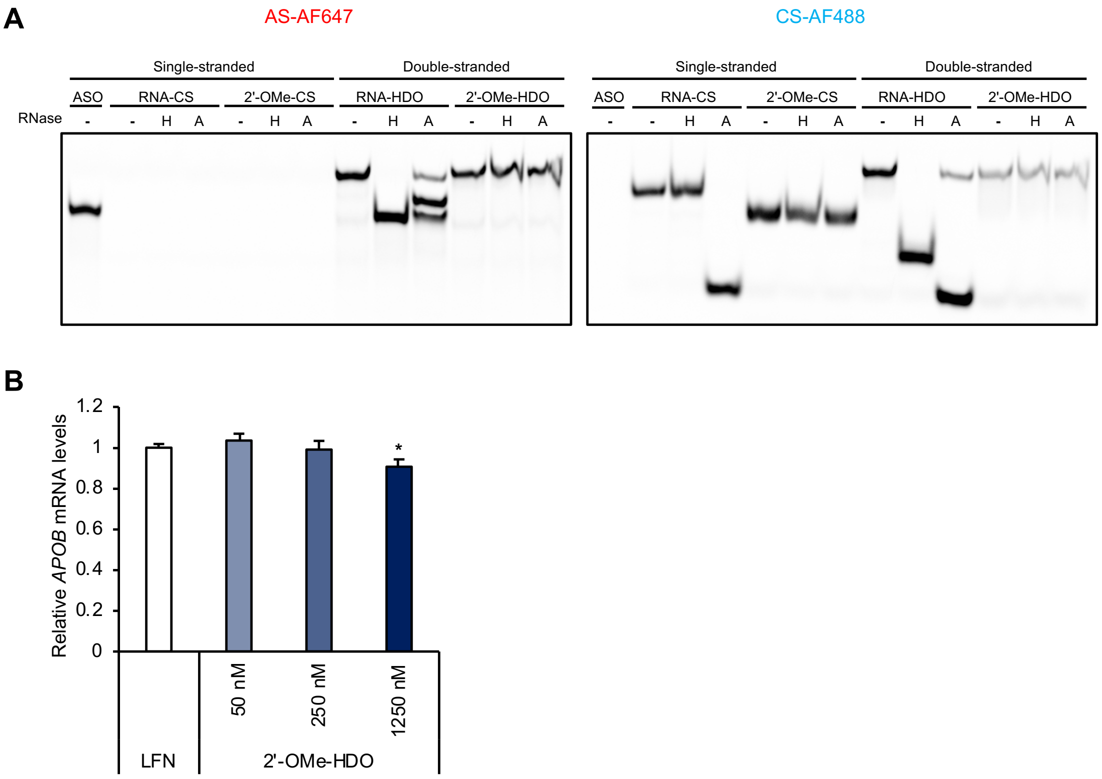

Figure S12. Cleavage-Independent Separation of 2'-OMe-HDO

(A) To evaluate the resistance of 2‘OMe-RNA for RNases, oligonucleotides targeting intron region of *APOB* mRNA, where antisense strand (AS) and complementary strand (CS) were labeled with AF647 and with AF488, respectively, were treated with RNase A or H. After electrophoresis in 20% polyacrylamide gel, fluorescence of each dye was imaged. The result showed that 2'-OMe-HDO was resistant to RNase H and A. (B) To see if cleavage-independent separation of 2'-OMe-HDO could induce antisense activity, dose-escalation study was performed. RT-qPCR analysis 24 h after transfection with 1250 nM 2'-OMe-HDO targeting intron region of *APOB* showed slight significant nuclear activity (normalized to *GAPDH*; * $p < 0.05$ vs lipofectamine (LFN) control; n = 3; mean ± SEM).

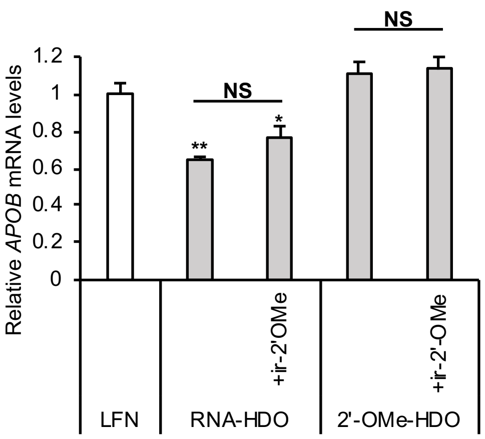

Figure S13. Co-Transfection with Irrelevant 2'-OMe Strand

To exclude a possibility that non-cleavable 2'-OMe strand competed with antisense strand (AS) or inhibited its activity in a sequence-independent manner, 50 nM RNA-HDO or 2'-OMe-HDO targeting intron *APOB* was co-transfected with 50 nM irrelevant full 2'-OMe strands targeting mouse *Malat* (ir-2'-OMe). RT-qPCR analysis 24 h after transfection showed no significant change between single transfection and co-transfection groups (normalized to *GAPDH*; * $p < 0.05$, ** $p < 0.01$ vs lipofectamine (LFN) control; NS, not significant; n = 3; mean ± SEM).

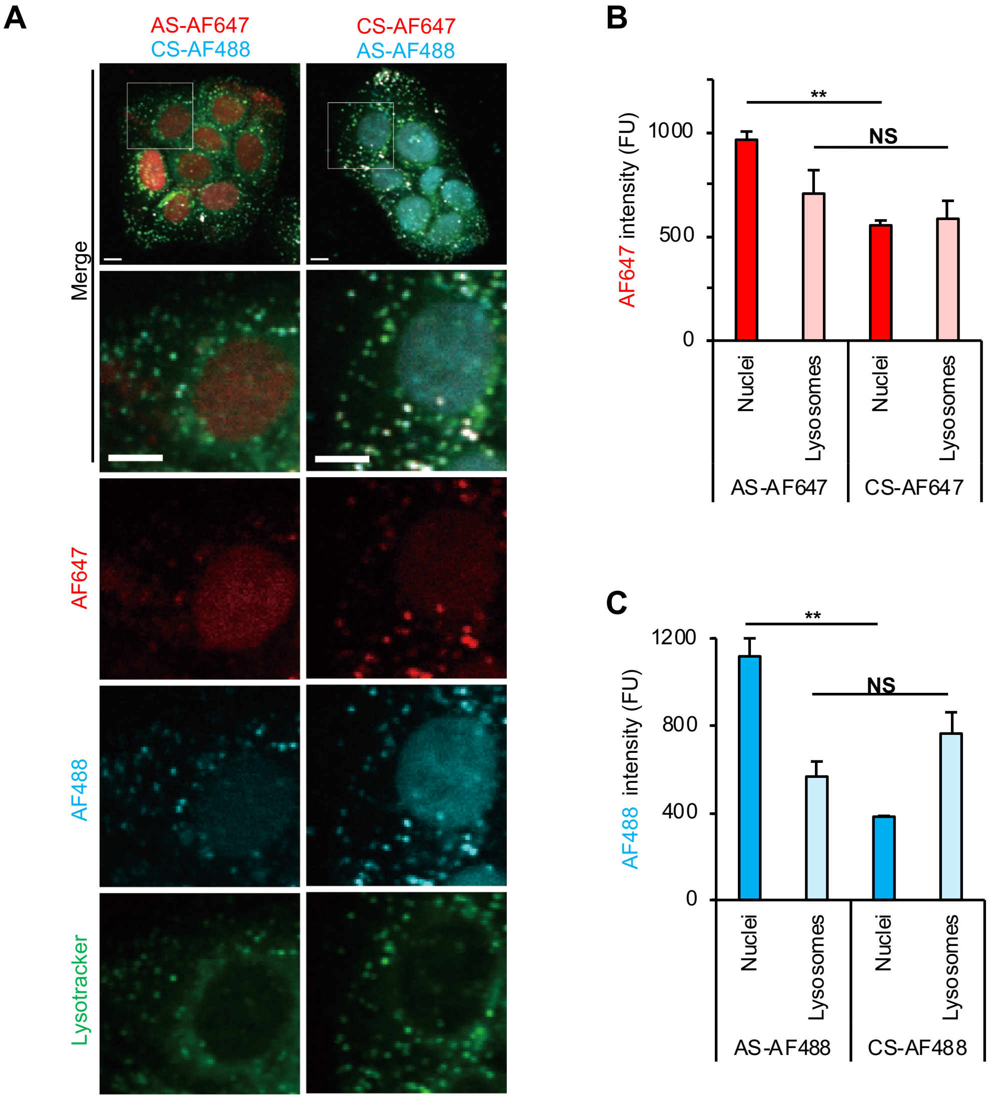

Figure S14. Accumulation of 2'-OMe-RNA Complementary Strand in Nuclei and Lysosomes

To evaluate localization of non-cleavable 2'-OMe-RNA complementary strand (CS), we imaged and evaluated its co-localization with nuclei or lysosomes. (A) Representative images 24 h after transfection with 50 nM 2'-OMe -HDO targeting intron *APOB*, composed of antisense strand (AS)-AF647 and CS-AF488 (left), or CS-AF647 and AS-AF488 (right). Lysosomes were labeled with lysotracker-RFP (green) , which was excited by a 560 nm laser and detected through a 595 (570-620) nm filter. AF647 signals (red) were excited by a 646 nm laser and detected through a 700 (663-738) nm filter. AF488 signals (cyan) were excited by a 488 nm laser and detected through a 525 (500-550) nm filter. Bar = 10 µm. (B, C) Mean signal intensities of AF647 (B), or AF488 (C) in nuclei and in lysosomes. Measurements from the experiment (A) presented as absolute values normalized to background levels. (** $p < 0.01$; NS, not significant; n = 3 images for every 50 cells, or 150 lysosomes; mean ± SEM).

Movie Legends

Movie 1. Separation, Cytosolic Release, and Rapid Nuclear Transport of HDO

Live cell time-lapse images of the HDO-releasing vesicle presented in Figure 2D, E. Images were taken, every 30 sec, just after transfection with 50 nM HDO targeting intron *APOB*, composed of antisense strand-AF647 (red) and complementary strand-AF568 (green). AF647 signals were excited by a 646 nm laser and detected through a 700 (663-738) nm filter. AF568 signals were excited by a 560 nm laser and detected through a 595 (570-620) nm filter. Bar = 10 µm.

Movie 2. Inflow Signals from HDO-Releasing Vesicles

(A, B) Live cell time-lapse images of cells which showed inflow signals from HDO-releasing vesicles, Images were taken in a high sensitivity setting compared to experiments in Figure 2, every 30 sec, just after transfection with 50 nM HDO targeting intron *APOB*, composed of antisense strand-AF647 (red) and complementary strand-AF568 (green). AF647 signals were excited by a 646 nm laser and detected through a 700 (663-738) nm filter. AF568 signals were excited by a 560 nm laser and detected through a 595 (570-620) nm filter. Bar = 10 µm.

Movie 3. Co-Localization of HDO Vesicles Foci with Early Endosomes

Live cell time-lapse images of the HDO releasing vesicle presented in Figures 3A and 3B. Cells expressing GFP-labeled *RAB5A* (cyan) were transfected with 50 nM HDO targeting intron *APOB* (antisense strand-AF647 (red), complementary strand-AF568 (green)). GFP signals were excited by a 488 nm laser and detected through a 525 (500-550) nm filter. AF647 signals were excited by a 646 nm laser and detected through a 700 (663-738) nm filter. AF568 signals were excited by a 560 nm laser and detected through a 595 (570-620) nm filter. Images were taken just after transfection, every 1 min. Bar = 10 µm.

Movie 4. Co-Localization of HDO Vesicles Foci with Late Endosomes

Live cell time-lapse images of the HDO releasing vesicle presented in Figure 3D and 3E. Cells expressing GFP-labeled *RAB7A* (cyan) were transfected with 50 nM HDO targeting intron *APOB* (antisense strand-AF647 (red), complementary strand-AF568 (green)). GFP signals were excited by a 488 nm laser and detected through a 525 (500-550) nm filter. AF647 signals were excited by a 646 nm laser and detected through a 700 (663-738) nm filter. AF568 signals were excited by a 560 nm laser and detected through a 595 (570-620) nm filter. Images were taken just after transfection, every 2 min. Bar = 10 µm.
